# A chromosome-level genome assembly of Hong Kong catfish (*Clarias fuscus*) uncovers a sex-determining region

**DOI:** 10.1186/s12864-023-09394-2

**Published:** 2023-05-30

**Authors:** Chang-Xu Tian, Xing-Hua Lin, Da-Yan Zhou, Yu Chen, Yi-Jun Shen, Ming-Hui Ye, Cun-Yu Duan, Yu-Lei Zhang, Bin-Lan Yang, Si-Ping Deng, Chun-Hua Zhu, Guang-Li Li

**Affiliations:** 1 Fisheries College, Guangdong Ocean University, Zhanjiang, 524088 China; 2 Guangdong Research Center on Reproductive Control and Breeding Technology of Indigenous Valuable Fish Species, Guangdong Provincial Engineering Laboratory for Mariculture Organism Breeding, Guangdong Provincial Key Laboratory of Aquatic Animal Disease Control and Healthy culture, Zhanjiang, 524088 China; 3 Guangxi Introduction and Breeding Center of Aquaculture, Nanning, 530001 China; 4 Southern Marine Science and Engineering Guangdong Laboratory (Zhanjiang), Zhanjiang, 524088 China

**Keywords:** *Clarias fuscus*, Genome, Phylogenetic analysis, Sex chromosome, Gene

## Abstract

**Background:**

Hong Kong catfish (*Clarias fuscus*) is an ecologically and economically important species that is widely distributed in freshwater regions of southern China. Hong Kong catfish has significant sexual growth dimorphism. The genome assembly of the Hong Kong catfish would facilitate study of the sex determination and evolution mechanism of the species.

**Results:**

The first high-quality chromosome-level genome of the Hong Kong catfish was constructed. The total genome was 933.4 Mb, with 416 contigs and a contig N50 length of 8.52 Mb. Using high-throughput chromosome conformation capture (Hi-C) data, the genome assembly was divided into 28 chromosomes with a scaffold N50 length of 36.68 Mb. A total of 23,345 protein-coding genes were predicted in the genome, and 94.28% of the genes were functionally annotated in public databases. Phylogenetic analysis indicated that *C. fuscus* and *Clarias magur* diverged approximately 63.7 million years ago. The comparative genome results showed that a total of 60 unique, 353 expanded and 851 contracted gene families were identified in Hong Kong catfish. A sex-linked quantitative trait locus identified in a previous study was located in a sex-determining region of 30.26 Mb (0.02 to 30.28 Mb) on chromosome 13 (Chr13), the predicted Y chromosome. This QTL region contained 785 genes, of which 18 were identified as sex-related genes.

**Conclusions:**

This study is the first to report the chromosome-level genome assembly of Hong Kong catfish. The study provides an excellent genetic resource that will facilitate future studies of sex determination mechanisms and evolution in fish.

**Supplementary Information:**

The online version contains supplementary material available at 10.1186/s12864-023-09394-2.

## Background

Sex determination in fish is diverse and shows high plasticity, with different underlying mechanisms among species. Sex determination mechanisms in fish include genetic sex determination and environmental sex determination as well as joint regulation by genetic and environmental factors [1]. Fishes have almost all types of sex determination systems found in vertebrates. The two main types of sex determination systems in fish are XX/XY and ZZ/ZW. Most fish have homozygous sex chromosomes that cannot be distinguished by shape [2]. With recent advances in high-throughput sequencing technologies and bioinformatics methods, it has become possible to identify and characterize sex chromosomes with low divergence. Male and female chromosomes in genome assemblies have been obtained in a variety of fish.

The family Clariidae (airbreathing catfishes) in the class Actinopteri and order Siluriformes is widely distributed in freshwater regions of Africa and Asia. Clariidae consists of 117 species in 16 genera (https://www.fishbase.in/search.php). The genus *Clarias* comprising 61 species is the largest genus in Clariidae. *Clarias* is an excellent model for understanding the evolution of sex determination because the genus includes species with an unusual diversity of sex chromosome systems. For example, *Clarias anguillaris* and *Clarias ebriensis* have the ZZ/ZW sex determination system [3], *Clarias macrocephalus* and *Clarias fuscus* have the XX/XY sex determination system [4, 5], and *Clarias gariepinus* have XX/XY or ZZ/ZW sex determination system in different populations [6]. *Clarias batrachus*, *C. fuscus*, *C. macrocephalus*, and *C. gariepinus* are economically important species in Asian aquaculture (Food and Agriculture Organization of the United Nations, 10.4060/ca9229en). Furthermore, *C. fuscus* and *C. gariepinus* are sexually dimorphic, with differences in growth rates between males and females [7, 8]. Therefore, it is necessary to assemble chromosome-level genomes of these species to provide a basis for understanding the evolution of sex chromosomes of catfishes.

Hong Kong catfish (*Clarias fuscus*), characterized by high adaptability, high nutritional value, and tender flesh, is an economically valuable freshwater fish species and is widely cultivated in southern China [9, 10]. It shows asynchronous gonadal development as well as multiple spawning, with a high reproductive capacity, age of sexual maturity of 10–12 months, and the ability to reproduce 3–4 times a year [11, 12]. Hong Kong catfish exhibit sexual dimorphism, particularly in body size. Under the same breeding conditions, the growth rate of Hong Kong catfish is significantly higher in males than in females [8]. When farmed Hong Kong catfish reach market size, females are in the gonadal development stage. Female abdomens are enlarged, and their gonads account for a much higher proportion of body weight than males, resulting in lower prices for females than for equally sized males. The monosex fish culture of Hong Kong catfish has important economic significance. The lack of genomic resources for this species is a serious impediment to monosex breeding. Thus, it is necessary to obtain the chromosome-level genome of the Hong Kong catfish as a resource for molecular breeding.

The genomes of Clariidae have been published for *Clarias batrachus* [13], *Clarias macrocephalus* [14] and *Clarias magur* [15]. The genomes of *Clarias fuscus*, a common catfish in Southeast Asia, have not been reported. Moreover, large-scale genomic analyses at the chromosome level were not well characterized in *Clarias* due to the fragmented assembly of *C. batrachus, C. macrocephalus* and *C. magur* genomes. In this study, the first high-quality chromosome-level genome of Hong Kong catfish was constructed by PacBio sequencing and high-throughput chromosome conformation capture (Hi-C) technology, and sex-determining candidate regions and sex- related genes were identified by sex-linked QTL.

## Results

### Genome sequencing and assembly

A total of 179.58 Gb of subreads base was obtained from the PacBio sequencing library, including 10,269,296 subreads with an average length of 17,488 bp and subread N50 of 25,523 bp. A total of 107.18 Gb of clean data was obtained from the HI-C sequencing library, including 725,685,598 clean reads with a Q20 of 96.7% and Q30 of 88.3%. A total of 56.85 Gb of clean data was obtained from the small-fragment genomic library, including 379,011,716 clean reads, with a Q20 of 95.8% and Q30 of 87% (Supplementary Table S1).

PacBio data were used to construct the preliminary genome assembly. The size of the preliminary genome assembly was 966.34 Mb, including 897 contigs, and the contig N50 was 8.18 Mb. Interrupting erroneous contigs in the preliminary genome based on HI-C data (957 contigs formed in total). The interrupted contigs were sorted to obtain chromosomal level genes. Finally, the Hong Kong catfish chromosome-level genome assembly was obtained with a length of 933.40 Mb, 28 chromosomes (containing 416 contigs), contig N50 = 8.52 Mb, and scaffold N50 = 35.68 Mb (Table 1, Supplementary Table S2). According to the BUSCO results, the genome contained 2,493 (96.40%) complete BUSCOs, including 2,405 single-copy BUSCOs and 88 duplicate BUSCOs (Supplementary Table S3). The results indicate that the genome assembly has high coverage and completeness.

**Table 1:** Genome assembly statistics for the *Clarias fuscus* genome

	Primary assembled genome	Chromosome-level genome assembly
Assembly size (bp)	966,348,140	933,400,497
Number of scaffolds	-	28
Scaffold N50 (bp)	-	35,683,429
Longest scaffold (bp)	-	48,393,616
Number of contigs	897	416
Contig N50 (bp)	8,179,350	8,522,588
Longest Contig max (bp)	26,641,720	26,641,720

### Genome annotation

**Fig. 1:**
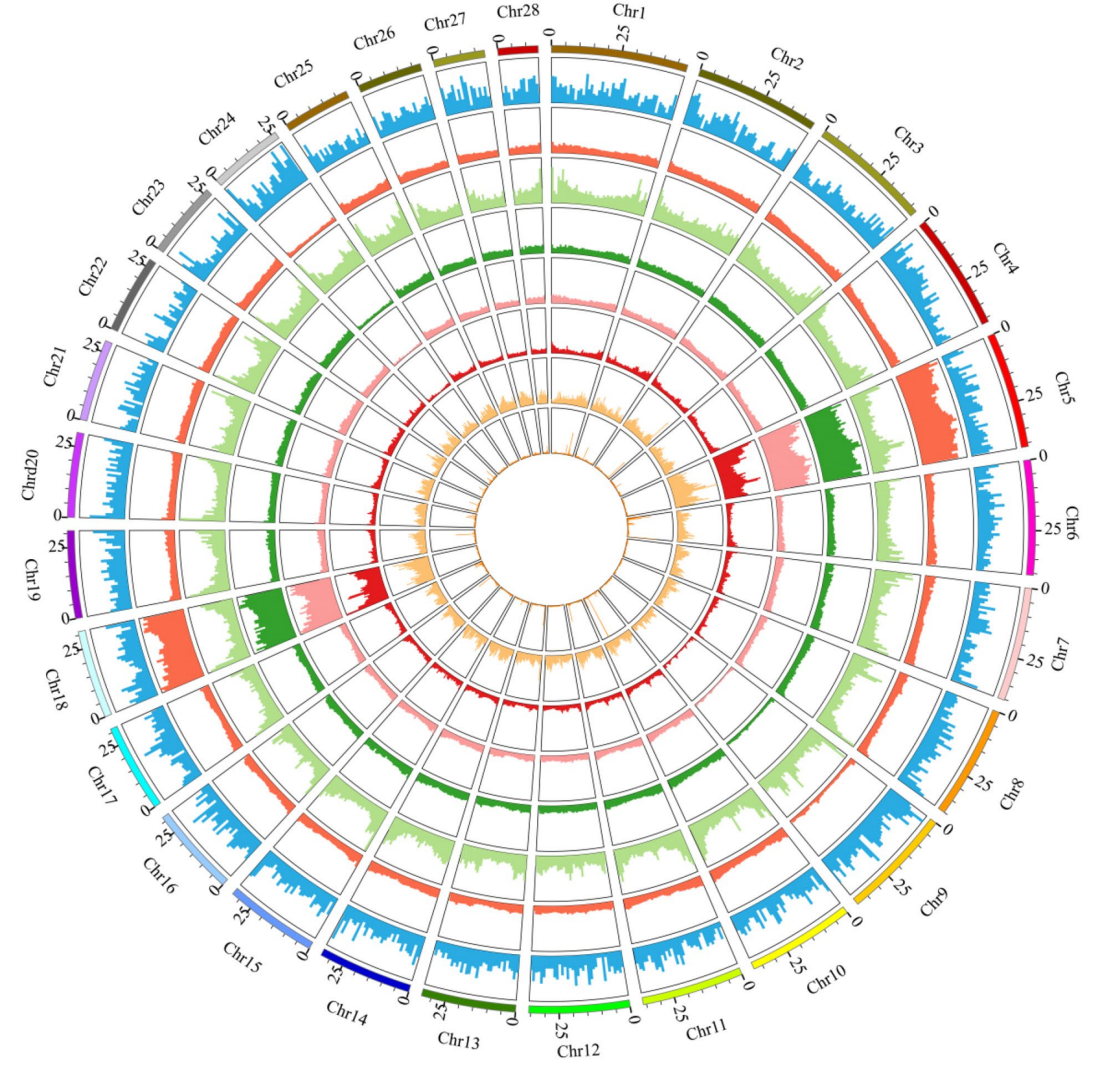
Genome characteristics of *C. fuscus*. From the outer to inner circle: gene distribution, repeat sequence distribution, tandem repeat sequence distribution, DNA transposon distribution, long terminal repeated (LTE) distribution, long interspersed nuclear element (LINE) distribution, short interspersed nuclear element (SINE) distribution and non-coding RNA (ncRNA) distribution. Bar height is proportional to number of items mapped to each genomic position

A total of 23,345 protein-coding genes were predicted in the Hong Kong catfish genome (Table 2; Fig. 1), with an average length of 19,425.94 bp. Of these, 18,493 genes encoded single transcripts and 4,852 genes encoded multiple transcripts, for a total of 32,216 transcripts. The characteristics of functional genes (length distribution of gene, coding sequence, exon and intron) were compared with those of other catadromous fishes, and the gene characteristics of Hong Kong catfish was similar to that of other Siluriformes (Supplementary Figure S1). The genes were annotated using the NR, TrEMBL, SwissProt, InterPro, KEGG, and GO databases. A total of 22,009 genes were annotated, representing 94.28% of all genes (Supplementary Table S4).

**Table 2:** Statistical summary of protein-coding gene prediction in the *Clarias fuscus* genome

Method	Software	Gene Number	Average gene length(bp)	Average CDS length(bp)	Average exon num per gene	Average exon length(bp)	Average intron length(bp)
De novo	AUGUSTUS	25,743	15,772.79	1,358.48	7.45	182.24	2,233.27
	Genscan	28,141	22,919.11	1,415.87	7.6	186.33	3,258.74
Homolog	*C.magur*	53,835	8,570.34	829.55	4.47	185.52	2,229.84
	*P.hypophthalmus*	38,935	11,969.30	1,086.77	6.13	177.27	2,121.17
	* S.meridionalis*	47,573	12,630.62	996.49	5.32	187.29	2,692.70
	*P.fulvidraco*	45,884	11,990.36	1,029.57	5.39	190.9	2,494.95
	*I.punctatus*	42,103	12,146.44	1,074.25	5.77	186.03	2,318.95
RNA-seq	-	14,265	17,907.57	1,443.98	9.26	310.59	1,819.36
Integration	MAKER	23,611	18,444.91	1,601.94	9.43	210.99	1,951.79
	PASA	23,345	19,425.94	1,601.47	9.44	248.83	2,024.33

The duplicated sequences accounted for 53.50% of the Hong Kong catfish genome (Fig. 1). DNA transposons (30.23%) were the most common TE type in the Hong Kong catfish genomic, followed by long terminal repeats (LTR, 20.27%) and long interspersed nuclear elements (LINEs, 10.14%) (Supplementary Table S5, Supplementary Table S6).

tRNAscan-SE, BLASTN, and Rfam were used to predict noncoding RNA, and a total of 12,110 tRNAs, 1,672 rRNAs, 506 snRNAs, and 278 miRNAs were predicted in the Hong Kong catfish genome (Fig. 1, Supplementary Table S7).

### Genomic evolution analysis

Gene family clustering was performed based on protein sequences of Hong Kong catfish. A total of 15,977 gene families and 3,185 single-copy genes were identified (Supplementary Figure S2, Supplementary Table S8). The gene families of 16 fishes (*Clarias fuscus, Latimeria chalumnae, Lepisosteus oculatus, Danio rerio, Electrophorus electricus, Astyanax mexicanus, C. magur, Silurus meridionalis, Pangasianodon hypophthalmus, Ictalurus punctatus, Pelteobagrus fulvidraco, Esox lucius, Gadus morhua, Oryzias latipes, Oreochromis niloticus*, and *Takifugu rubripes*) were compared. A total of 60 unique, 353 expanded and 851 contracted gene families were identified in Hong Kong catfish (Supplementary Table S9).

**Fig. 2:**
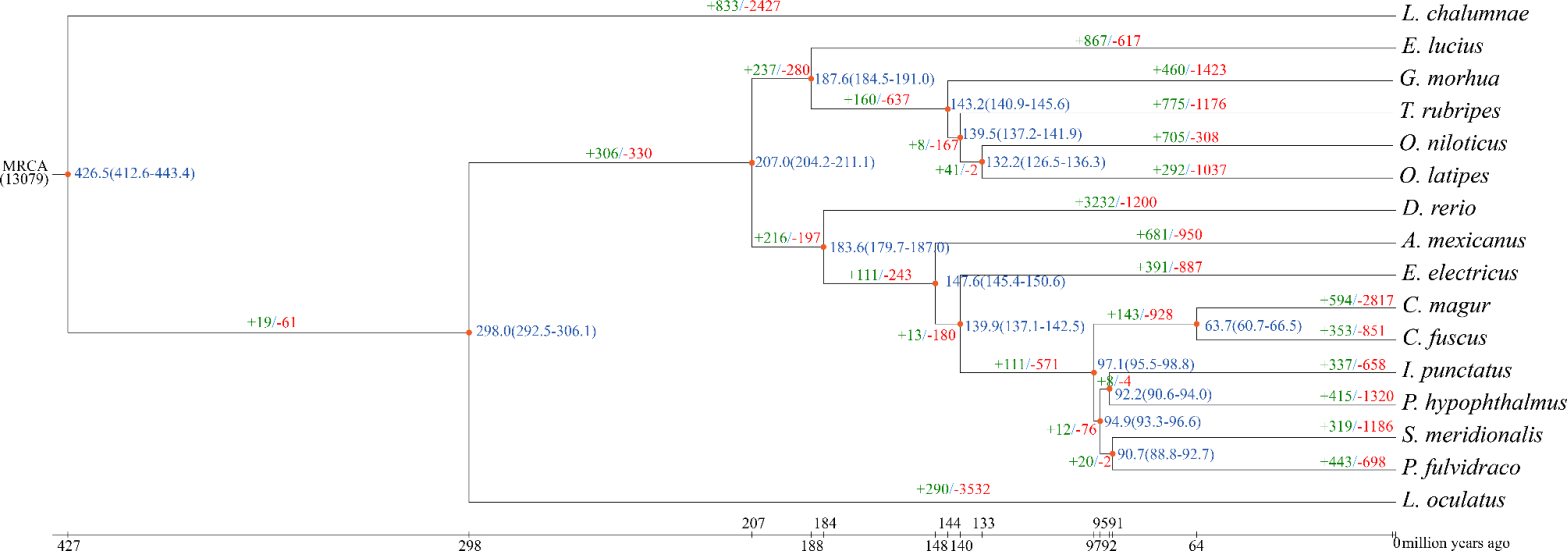
Phylogenetic analysis of 16 fishes. At each node, the predicted divergence time (million years ago) is marked. The green number on each branch represents the number of expanded gene families, and red indicates the number of contracted gene families

Phylogenetic analyses showed that Hong Kong catfish and *C. magur* are clustered in a single branch. In addition, the Hong Kong catfish is closely related to S. *meridionalis, P. hypophthalmus, I. punctatus*, and *P. fulvidraco*, which belong to the order Siluriformes. Furthermore, Hong Kong catfish diverged from its most closely related species, *C. magur*, about 63.7 (60.7–66.5) million years ago (Fig. 2).

**Fig. 3:**
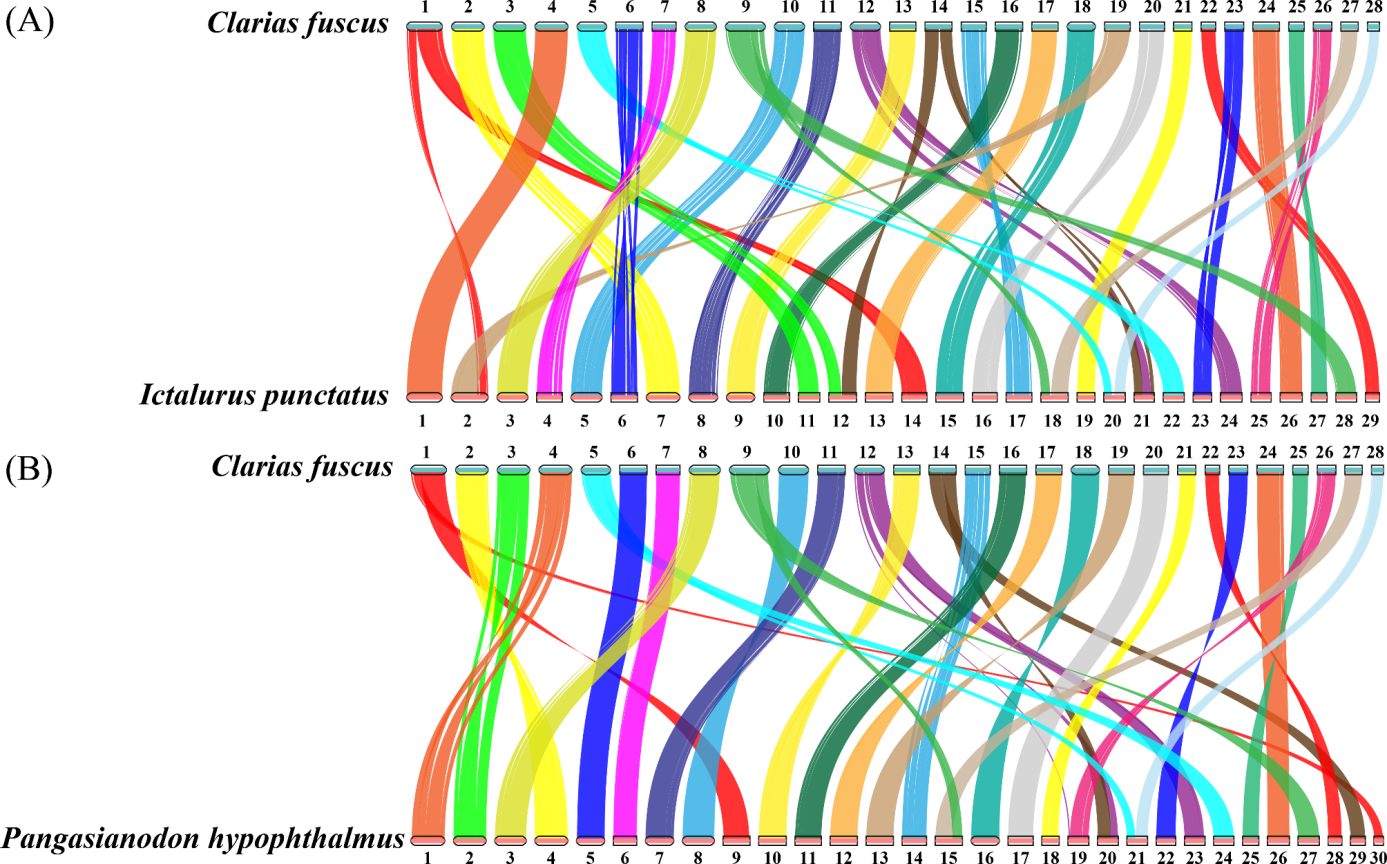
Collinearity analysis of *C. fuscus* and other teleost genome. (A) Collinearity analysis of *C. fuscus* and *I. punctatus* genomes. Blue and orange lines represent the chromosomes of *C. fuscus* and *I. punctatus*, respectively. (B) Collinearity analysis of *C. fuscus* and *P. hypophthalmus* genomes. Blue and orange lines represent the chromosomes of *C. fuscus* and *P. hypophthalmus*, respectively

All 28 chromosomes of Hong Kong catfish were smaller than the 29 chromosomes of *I. punctatus* and 30 chromosomes of *P. hypophthalmus* (Fig. 3). In comparison with the two species of catfish, Hong Kong catfish Chr1, 9, 12, and 14 underwent chromosomal breakage and recombination events during evolution. In addition, chromosome breakage and recombination events were found on Chr3 and Chr5 in comparison with the *I. punctatus* chromosomes.

### Identification of the sex-determination region

**Fig. 4:**
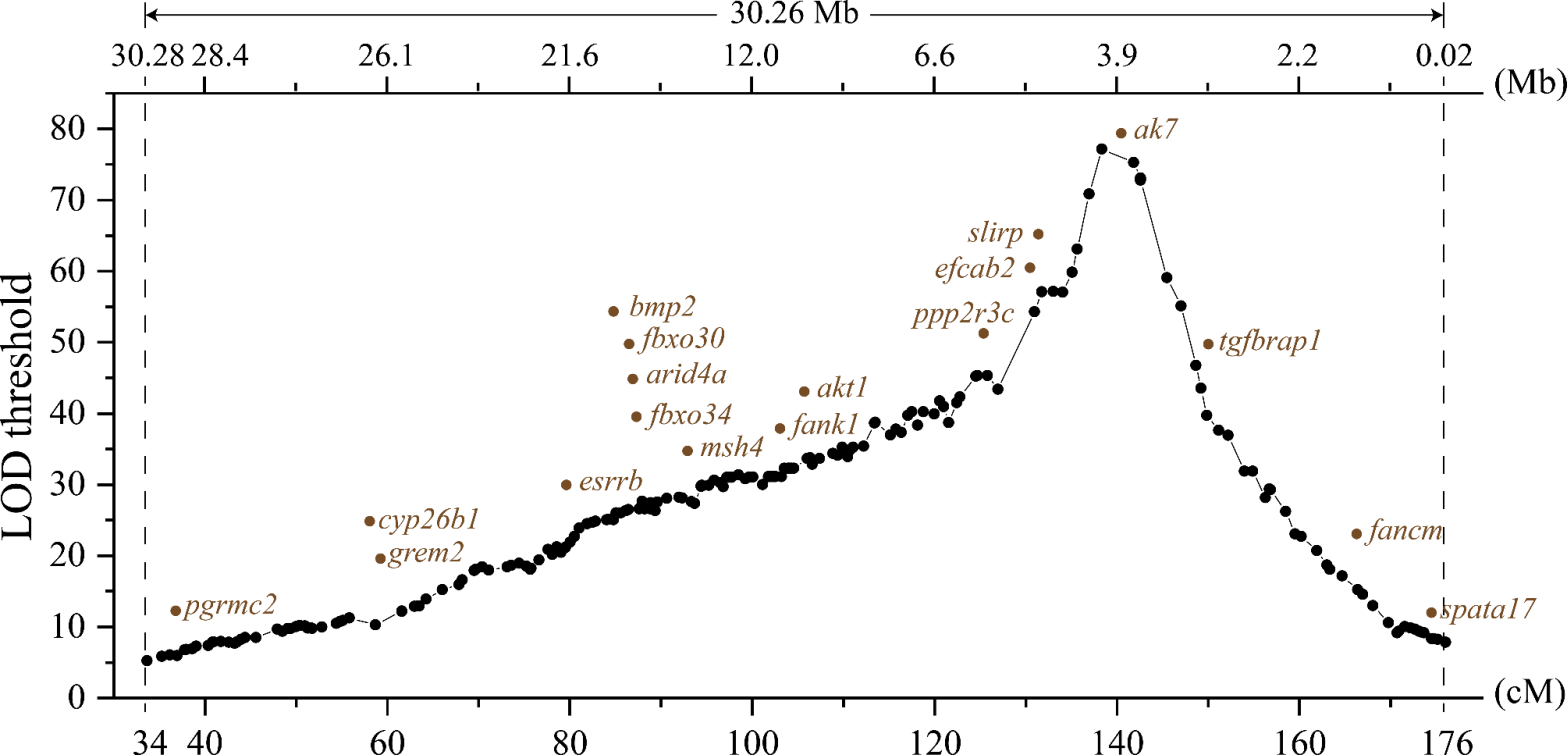
Location of sex-related genes in the QTL interval of *Clarias fuscus.* The lower abscissa is the genetic distance, in centimorgans (cM); the upper abscissa is the genomic position, in megabases (Mb); the ordinate is the LOD threshold; black dots are SNP markers; brown dots are sex-related genes

Each LG of the genetic linkage map corresponded to one chromosome of the genome assembly (Table 3). According to the mapping results, the sex-linked QTL was located on Chr13 of the genome assembly. The QTL (33.64–176.02 cM) corresponded to a 30.26 Mb region (from 0.02 to 30.28 Mb) on the genome (Fig. 4). Based on the gene annotation results for chr13, 785 genes were identified in the sex-linked QTL (Supplementary Table S10). Among the 785 genes in the sex-linked QTL, 18 sex related genes were identified, including fanconi anemia group m protein (*fancm*), EF-hand calcium-binding domain-containing protein 2 (*efcab2*), SRA stem-loop-interacting RNA-binding protein (*slirp*), F-box protein 30 (*fbxo30*), F-box protein 34 (*fbxo34*), bone morphogenetic protein 2 (*bmp2*), and AT-rich interactive domain-containing protein 4a (*arid4a*), transforming growth factor-beta receptor-associated protein 1 (*tgfbrap1*), adenylate kinase 7 (*ak7*), protein phosphatase 2 regulatory subunit B’’gamma (*ppp2r3c*), fibronectin type III and ankyrin repeat domains 1 (*fank1*), akt serine/threonine kinase 1 (*akt1*), muts homolog 4 (*msh4*), estrogen-related receptor b (*esrrb*), progesterone receptor membrane component 2 (*pgrmc2*), cytochrome P450 26B1 (*cyp26b1*), gremlin 2 (*grem2*), and spermatogenesis associated 17 (*spata17*) (Table 4).

**Table 3:** Correspondence between the physical genome and genetic linkage map of *Clarias fuscus*

Chromosome	Linkage group	Physical length (Mb)	Genetic size (cM)	No. of genes	Gene density (genes/Mb)
Chr1	LG1	48.39	224.23	1166	24.09
Chr2	LG2	44.23	220.99	1075	24.31
Chr3	LG3	42.57	237.72	989	23.23
Chr4	LG4	41.62	218.9	1055	25.35
Chr5	LG5	41.37	195.5	958	23.16
Chr6	LG6	40.66	203.89	870	21.39
Chr7	LG7	40.31	160.8	779	19.33
Chr8	LG8	40.03	200.74	966	24.13
Chr9	LG9	39.55	194.64	1357	34.31
Chr10	LG10	38.21	180.62	947	24.78
Chr11	LG11	36.13	183.01	853	23.61
Chr12	LG12	35.68	182.52	936	26.23
Chr13	LG13	33.45	176.02	841	25.15
Chr14	LG14	33.34	183.41	865	25.95
Chr15	LG15	33.02	176.52	817	24.74
Chr16	LG16	31.58	176.18	849	26.89
Chr17	LG17	31.36	158.04	815	25.99
Chr18	LG18	31.29	166.9	852	27.23
Chr19	LG19	31.02	189.28	810	26.12
Chr20	LG20	29.97	183.35	777	25.93
Chr21	LG21	29.01	137.13	583	20.10
Chr22	LG22	27.81	133.68	501	18.02
Chr23	LG23	26.80	148.16	625	23.32
Chr24	LG24	26.60	157.16	932	35.04
Chr25	LG25	24.07	149.38	511	21.23
Chr26	LG26	23.00	133.82	582	25.31
Chr27	LG27	18.28	110.14	540	29.55
Chr28	LG28	14.27	87.42	392	27.46
Total	-	933.40	4870.15	23,243	24.90

**Table 4:** Sex-related genes in the sex-linked QTL region of *Clarias fuscus*

Gene ID	Gene symbol	NR Annotation
gene-Cfus03917	*spata17*	spermatogenesis-associated protein 17
gene-Cfus03930	*fancm*	Fanconi anemia group M protein
gene-Cfus03992	*tgfbrap1*	transforming growth factor-beta receptor-associated protein 1
gene-Cfus04009	*ak7*	Adenylate kinase 7
gene-Cfus04022	*slirp*	SRA stem-loop-interacting RNA-binding protein
gene-Cfus04028	*efcab2*	EF-hand calcium-binding domain-containing protein 2
gene-Cfus04046	*ppp2r3c*	Protein phosphatase 2 regulatory subunit B’’gamma
gene-Cfus04126	*akt1*	AKT serine/threonine kinase 1
gene-Cfus04155	*fank1*	Fibronectin type III and ankyrin repeat domains 1
gene-Cfus04263	*msh4*	MutS homolog 4
gene-Cfus04356	*fbxo34*	F-box only protein 34
gene-Cfus04361	*arid4a*	AT-rich interactive domain-containing protein 4 A
gene-Cfus04377	*fbxo30*	F-box only protein 30
gene-Cfus04417	*bmp2*	bone morphogenetic protein 2
gene-Cfus04472	*esrrb*	steroid hormone receptor ERR2
gene-Cfus04599	*grem2*	gremlin 2
gene-Cfus04602	*cyp26b1*	cytochrome P450 26B1
gene-Cfus04663	*pgrmc2*	membrane-associated progesterone receptor component 2

## Discussion

Some fish species exhibit sexual growth dimorphism, including significant differences in body size and growth rate between males and females. Thus, monosex culture of the sex with dominant growth will greatly improve the economic value of fish species. For example, in *Cyprinus carpio*, *Oncorhynchus mykiss*, and *Cynoglossus semilaevis*, female fish grow faster than males [16–18]. In contrast, some fish males grow faster than females, such as *C. fuscus, P. fulvidraco*, *Oreochromis aureus*, and *I. punctatus* [19–21]. Therefore, the assembly of a high-level reference genome and screening of sex-related genes are of great significance for the development of the single-sex aquaculture industry for catfish and clarifies the sex determination mechanism in the species.

In the present study, the high-quality chromosome-level genome of Hong Kong catfish (*C. fuscus*) was assembled. The Hong Kong catfish chromosome-level genome assembly length was 933.40 Mb and scaffold N50 = 35.68 Mb. In a comparative analysis of Siluriformes [13–15, 22–25], the genome size was smaller than that of *C. magur* (941 Mb) and larger than those of *C. macrocephalus* (883 Mb), *C. batrachus* (821.8 Mb), *I. punctatus* (783 Mb), and *P. fulvidraco* (732.8 Mb). The scaffold N50 values (35.68 Mb) were higher than those of *C. magur* (1.3 Mb), *C. macrocephalus* (80.8 kb), *C. batrachus* (361.2 kb), *I. punctatus* (7.73 Mb), and *P. fulvidraco* (25.8 Mb). These results show that the construction of a high-quality Hong Kong catfish genome assembly in this study. The Hong Kong catfish genome assembly was anchored to 28 chromosomes, which is consistent with previously reported karyotype data [26]. A total of 23,345 functional genes and 53.50% repetitive sequences were identified by annotation of the genome of Hong Kong catfish. In addition, comparing the repeated sequences of Hong Kong catfish with other Siluriformes, which was higher than estimates for *C. magur* (43.72%) [15], *P. fulvidraco* (43.31%) [27], *I. punctatus* (41.1%) [28], *C. macrocephalus* (38.28%) [14] and *C. batrachus* (30.3%) [13].

Previous studies of sex-linked QTL mapping have identified a sex-linked QTL in Hong Kong catfish in LG13 (Lin et al., 2022). By comparing the SNP markers of the genetic map with the Hong Kong catfish genome, LG13 was found to correspond to Chr13 in this study. This QTL interval occupied a 30.26 Mb region of Chr13 (ranging from 0.02 to 30.28 Mb) and contained 785 genes. The total length of Chr13 was 33.45 Mb, and the QTL region occupied 90.33% of the chromosome. It is speculated that Chr13 is the sex chromosome of Hong Kong catfish. Sex chromosomes have been identified in some Siluriformes, such as *I. punctatus*, *P. fulvidraco, S. meridionalis* and *P. hypophthalmus* [29–32]. Based on the identification of sex chromosomes, candidate sex-determining genes have been identified, such as breast cancer anti-estrogen resistance protein 1 (*bcar1*) in *I. punctatus*, PDZ domain-containing gene (*pfpdz1*) in *P. fulvidraco*, anti-Müllerian hormone receptor type 2 on the Y chromosome (*amhr2y*) in *S. meridionalis* and male-specific duplication of *amhr2* (*amhr2by*) in *P. hypophthalmus* [29–32]. Based on the genome comparison results of the Hong Kong catfish and other fishes (Fig. 3), the *bcar1* of *I. punctatus* is located on chromosome 4 [29], which compared to chromosome 7 of the Hong Kong catfish. The *amhr2by* of *P. hypophthalmus* is located on chromosome 4 [32], which compared to chromosome 2 of Hong Kong catfish. The sex-determining regions and genes of the above two Siluriformes fishes were not mapped to Chr13 of the Hong Kong catfish. This may be due to the fact that the genomes of Siluriformes fishes are generally large and multiple genome duplication events have occurred. During the replication process, the sex-determination-related mechanism of Siluriformes fishes also changed, thus presenting a variety of inconsistent sex-determination genes in Siluriformes fishes. In summary, the identification of sex chromosomes in Hong Kong catfish provides a basis for exploring the sex determination mechanism and sex determination genes.

Based on the gene annotation results for chr13, 18 sex related genes were identified among 785 genes in the sex-linked QTL. Among the 18 sex related genes, five infertility-related genes were identified, including *fancm, slirp*, *arid4a*, *ak7*, and *akt1*. Hypogonadism have been noted in both male and female mice with a *fancm* deficiency [33], and a homozygous *fancm* frameshift pathogenic variant causes male infertility in humans [34]. The *slirp* gene regulates male fertility, and its loss of function will alter the sperm structure and mitochondrial morphology [35]. *arid4a* regulates male fertility. Mice lacking *arid4a* and *arid4b* (Arid4a^(−/−)^ and Arid4b^(+/−)^) exhibit the progressive loss of male fertility with hypogonadism and spermatophore hypoplasia [36]. The homozygous missense mutation L673P in *ak7* leads to primary male infertility and multiple morphological anomalies of the flagella [37]. Female akt1(-/-) mice display reduced fertility and abnormal estrous cyclicity [72].

In addition, the remaining 13 sex-related genes were associated with gametogenesis, including *efcab2, ppp2r3c, fank1, cyp26b1, spata17, bmp2, tgfbrap1, pgrmc2, grem2, fbxo30, fbxo34* and *esrrb.* The *efcab2* gene was detected only in mouse tissues of the testis and may be involved in the control of sperm flagellar motility. In addition, it was specifically localized in spermatogenic cells from primary spermatocytes to elongate spermatids within the seminiferous epithelium; however, neither spermatogonia nor somatic cells were expressed [38]. The PPP2R3C protein is involved in the ontogeny of multiple organs and is especially critical for testis development and spermatogenesis [68]. PPP2R3C acts as a regulator of the PP2A and PP5 phosphatase holoenzymes and may be critical in the early signaling cascade controlling human sex determination [69]. *fank1* is specifically expressed in the testis from the meiotic to haploid stages of spermatogenesis and may play a key role in spermatogenesis by functioning as a transcription factor [39]. Additionally, *fank1*-knockout mice have reduced sperm counts and increased apoptotic spermatocytes, which are mainly spermatogonia and spermatocytes [40]. *cyp26b1* maintains low levels of retinoic acid in the developing testis, thereby preventing entry into meiosis and acting as a survival factor to prevent apoptosis in male germ cells [41]. The overexpression of SPATA17 leads to accelerated apoptosis in a zebrafish spermatogonial cell line [42]. *bmp2* is a member of the TGF-β superfamily and is expressed in an ovary-specific manner during early gonadal development [43]. Overexpression of the *tgfbrap1* gene suppresses granulosa cell E2 and P4 secretion, while *tgfbrap1* knockdown enhances E2 and P4 secretion, suggesting that *tgfbrap1* regulates apoptosis in goose follicle granulosa cells [44]. *pgrmc2* may regulate the maturation of zebrafish oocytes by regulating receptors and steroids in the ovary [45]. Female g*rem2*-knockout mice have irregular fertility and estrous cycles accompanied by a significant reduction in ovarian anti-Mullerian hormone production by growing follicles [46]. The *fbxo30* gene regulates chromosome segregation during oocyte meiosis [47]. *fbxo34* regulates the G2/M transition and late entry of meiotic oocytes [48]. *msh4* is an important gene involved in meiosis, and mutations in this gene may be associated with female infertility and male non-obstructive azoospermia [49]. *esrrb* functions upstream of *bmp4* (bone morphogenetic protein 4) in the extraembryonic ectoderm, regulating primordial germ cell development in mice [50]. The discovery of the above sex-related genes provides methodological guidance and an important tool for breeding techniques aimed at Hong Kong catfish sex control and lays the foundation for subsequent studies of mechanisms underlying sex determination.

In this study, the chromosome-level genome and sex-linked loci of Hong Kong catfish provide a research basis for subsequent developmental sex-linkage markers.Subsequently, the genetic male juvenile Hong Kong catfish were identified by sex-linked markers, and the biological sex of the genetic male Hong Kong catfish was changed by artificial sex reversal. So as to obtain the Hong Kong catfish of genetic male and physiological female. Then, sex-reversed Hong Kong catfish and normal male Hong Kong catfish were bred as parents, and YY Hong Kong catfish was sex-linked markers by sex-linked markers. Finally, based on YY Hong Kong catfish, the family groups of single-males Hong Kong catfish can be produced. In addition, sex-linked loci also provide candidate regions for identifying sex-determining genes. Subsequently, the sex-determining gene of Hong Kong catfish was knocked out by gene knockout technology, and a single-sex Hong Kong catfish population could be produced. In summary, the chromosome-level genome and sex-linked loci of the Hong Kong catfish can promote the development of the monosex culture industry of the Hong Kong catfish.

## Conclusions

The chromosomal-level genome assembly of Hong Kong catfish was determined. The continuity and completeness of the Hong Kong catfish genome were consistent with those of other high-quality teleost fish genomes. Accordingly, these data provide a useful reference for systems biology and comparative genome evolution analyses. A sex-linked QTL was focused on Chr13, which was predicted as the Y chromosome, and eighteen genes in this region were considered as sex-related genes. The newly established reference genome provides an important bases for aquaculture and artificial breeding of Hong Kong catfish.

## Methods

### Sample preparation

Hong Kong catfish were obtained from the Guangxi Introduction and Breeding Center of Aquaculture (Nanning, China). One male (body weight 180.53 g, body length 26.8 cm) and one female (body weight 167.18 g, body length 25.5 cm) Hong Kong catfish of similar sizes were collected. After anesthesia in a bath of eugenol (1:10,000), dissection was performed after decapitation, and the mid-section of gonadal tissue was cut from each individual and stored at 4 °C in Bouin’s Fixative Solution. The head kidney, muscle, brain, liver, gonads (testis and ovaries), kidney, heart, stomach, spleen, and skin tissue were collected, snap-frozen in liquid nitrogen, and stored at -80 °C. Genomic DNA was extracted from muscle tissue of male fish using the CTAB method. The concentration and quality were examined using a NanoDrop 2000 spectrophotometer (NanoDrop Technologies, Wilmington, DE, USA) and 0.8% agarose gel electrophoresis. Total RNA was extracted from each tissue type of male and female Hong Kong catfish using the TRIzol method. The RNA purity and integrity were examined using a NanoDrop 2000 spectrophotometer (NanoDrop Technologies) and a Bioanalyzer 2100 system (Agilent Technologies, Santa Clara, CA, USA). The extent of RNA degradation was examined by 1.5% agarose gel electrophoresis.

### Library construction and sequencing

Genomic PacBio sequencing libraries were constructed using the SMRTbell Express Template Prep Kit 2.0 (Pacific Biosciences, Menlo Park, CA, USA) according to the manufacturer’s instructions. Sequencing libraries were used to determine the concentration and fragment size distribution of samples using the FEMTO Pulse System (Agilent) and Qubit 3.0 fluorometer (Life Technologies, Carlsbad, CA, USA). Fragment size selection was performed using the BluePippin system (Sage Science, Beverly, MA, USA) to remove sequenced fragments below 25 kb. After the libraries were tested, sequencing was performed on the PacBio Sequel II platform (Pacific Biosciences). For the construction of the Hi-C library, 1 g of muscle tissue was used to prepare a library according to previously established protocols [51]. The Hi-C library was sequenced on the MGI-SEQ 2000 platform (BGI, China). Clean data were obtained using HTQC (v1.92.310) [52] for quality control.

Small-fragment genomic libraries were constructed using the VAHTS Universal DNA Library Prep Kit for MGI (Vazyme, Nanjing, China) according to the manufacturer’s recommendations. After library construction, the concentration and fragment size distribution of samples were determined using a Qubit 3.0 fluorometer (Life Technologies) and Bioanalyzer 2100 system (Agilent Technologies). After the libraries were tested, sequencing was performed on the MGI-SEQ 2000 platform. HTQC (v1.92.310) [52] was used to remove adapters, duplicate reads, N-containing sequences (≥ 10%), and low-quality (Qphred ≤ 5) reads from the raw data.

Whole tissue mRNAs from female and male fish were used to construct transcriptome libraries for male and female fish, respectively. The libraries were constructed using the VAHTS Universal V6 RNA-seq Library Kit for MGI (Vazyme) according to the manufacturer’s instructions. Library quality and size were determined using a Qubit 3.0 Fluorometer (Life Technologies) and a Bioanalyzer 2100 system (Agilent Technologies). Sequencing was performed on the MGI-SEQ 2000 platform. SOAPnuke (v2.1.0) [53] was used to remove adapters, N-containing sequences (≥ 0.5%), and low-quality (Qphred ≤ 20) reads.

### Genome assembly

A preliminary genome assembly was obtained using Mecat (v2.0) [54] for PacBio sequencing. After the preliminary assembly was completed, error correction was performed using SMRT Link (v8.0). Then, the small-fragment data were used for genome polishing using pilon (v1.22) [55]. Finally, the contig-level genome assembly was obtained. The genomic HI-C sequencing data were compared using BWA-mem (v.0.7.16a-r1181) [56], and single-end reads and sequences beyond 500 bp from the enzyme cut site were removed. Filtered Hi-C data were used for chromosome construction. The contigs were clustered, sorted, and oriented using 3D-DNA [57] to obtain chromosome-level genomes. The chromosome-level genome was visualized and error-corrected using JuiceBox [58]. The completeness of the Hong Kong catfish genome assembly was assessed using BUSCO (v3.0.2) [59] based on the single-copy homologous gene set (vertebrata_odb9) in OrthoDB.

### Genome prediction and annotation

Homology-based annotation and *de novo* annotation methods were used to identify repetitive sequences in the genome. First, transposable element (TE) sequences were searched from the Repbase (v.21.01) [60] database based on homology using RepeatMasker (v.4.09) and RepeatProteinMask (v.4.09) [61]. Next, RepeatModeler (v.1.0.11) [62] and LTRfinder (v1.0.5) [63] were used to construct the Hong Kong catfish repeat sequence database *de novo*. Repeat sequences were then identified from the constructed database using RepeatMasker (v.4.09). In addition, TRF [64] was used to identify tandem repeat sequences. Finally, the results obtained by homology-based annotation and *de novo* annotation were integrated, and the final annotation results of duplicate sequences were obtained by removing the non-redundant parts after overlapping.

The homology annotation, de novo annotation, and transcriptome annotation were used to predict the structure and function of protein-coding genes. The coding-gene annotation information of *C. magur, P. hypophthalmus, S. meridionalis, P. fulvidraco*, and *I. punctatus* were selected for homology-based annotation. Genomes of these species were aligned to the Hong Kong catfish genome using TblastN [65]. Augustus (v3.3) [66] and Genscan (v3.0.4) [67] were used for *de novo* annotation. TopHat [68] was used to match the transcriptome data to the Hong Kong catfish genome. MAKER (v3.00) [69] was used to integrate the gene sets obtained by various methods into a non-redundant gene set. Annotation information for non-coding regions and variable shears was added using PASA. Finally, the proteins in the gene set were functionally annotated with protein databases (SwissProt, TrEMBL, KEGG, InterPro, GO, and NR).

tRNAscan-SE (v1.3.1) [70] was used to find tRNA sequences in the genome. BLASTN (v2.6.0) [71] was used to find rRNAs in the genome. In addition, INFERNAL [72] in Rfam (v14.1) was used to predict miRNA and snRNA sequences in the genome.

### Genome evolution analysis

Gene family clustering was performed based on protein sequences of Hong Kong catfish and others 15 fish species, including *L. chalumnae, L. oculatus, D. rerio, E. electricus, A. mexicanus, C. magur, S. meridionalis, P. hypophthalmus, I. punctatus, P. fulvidraco, E. lucius, G. morhua, O. latipes, O. niloticus*, and *T. rubripes*. The protein sequences for each species were clustered based on sequence similarity using OrthoMCL (v14-137) [73]. Phylogenetic trees were constructed based on the shared single-copy direct homologous genes obtained by gene family clustering. Phylogenetic trees were constructed by the maximum likelihood method using RAxML (v8.2.11) [74]. The divergence time was predicted using MCMCtree in PAML (v4.9e) [75] and TimeTree for calibration. Using CAFÉ [76], random birth and death processes were used to simulate gene family expansion and contraction events for each lineage on the phylogenetic tree. The chromosomal genomes in Hong Kong catfish, *I. punctatus*, and *P. hypophthalmus* were included in a collinearity analysis using Mummer (v4.0.0beta2) [77].

### Identification of the sex-determination region and potential sex-related genes

In a previous study, a high-density linkage map of Hong Kong catfish with 6453 SNP markers was constructed, and a sex-linked QTL was identified with a total map distance of 142.38 cM and 225 SNP markers [5]. SNP markers from the genetic linkage map were mapped to the chromosome-level genome using bwa (version 0.7.17) to determine the correspondence between the linkage groups (LGs) of the genetic linkage map and the chromosomes (chrs) of the genome assembly. The genomic region corresponding to the sex-linked QTL was obtained based on a comparison of the 225 SNP markers. The number of genes in the sex-linked QTL and annotation information were obtained. Gene functions were obtained using Swiss-Prot, PubMed, and NCBI databases.

## Electronic supplementary material

Below is the link to the electronic supplementary material.

Supplementary Material 1

## Data Availability

The PacBio sequencing data, Hi-C sequencing data, small-fragment genomic sequencing data and transcriptome data of Hong Kong catfish has been submitted the Genome Sequence Archive in National Genomics Data Center (https://ngdc.cncb.ac.cn/gsa/) under accession number CRA007461. The final chromosome assembly and gene annotation of *Clarias fuscus* has been submitted the Genome Warehouse in National Genomics Data Center (https://bigd.big.ac.cn/gwh) under accession number GWHBKKP00000000.

## References

[1] Mei J, Gui JF (2015). Genetic basis and biotechnological manipulation of sexual dimorphism and sex determination in fish. Sci China-Life Sci.

[2] Bachtrog D, Mank JE, Peichel CL, Kirkpatrick M, Otto SP, Ashman TL, Hahn MW, Kitano J, Mayrose I, Ming R (2014). Sex determination: why so many Ways of doing it?. PLoS Biol.

[3] Pandey N, Lakra WS (1997). Evidence of female heterogamety, B-chromosome and natural tetraploidy in the asian catfish, Clarias batrachus, used in aquaculture. Aquaculture.

[4] Nguyen DHN, Ponjarat J, Laopichienpong N, Kraichak E, Panthum T, Singchat W, Ahmad SF, Muangmai N, Duengkae P, Peyachoknagul S (2021). Genome-wide SNP analysis suggests male heterogamety in bighead catfish (Clarias macrocephalus, Gunther, 1864). Aquaculture.

[5] Lin X, Tan J, Shen Y, Yang B, Zhang Y, Liao Y, Wang B, Zhou D, Li G, Tian C. A high-density genetic linkage map and QTL mapping for sex in *Clarias fuscus*. Aquaculture; 2022. p. 561.

[6] Nguyen DHM, Panthum T, Ponjarat J, Laopichienpong N, Kraichak E, Singchat W, Ahmad SF, Muangmai N, Peyachoknagul S, Na-Nakorn U (2021). An investigation of ZZ/ZW and XX/XY sex determination Systems in North African Catfish (*Clarias gariepinus*, Burchell, 1822). Front Genet.

[7] Fazazi AOT, Abayomi JA, Olabode GA, Adejumoke AL (2019). Sexual dimorphism in body weight, morphometric measures and indices of african catfish (Clarias gariepinus). Aquaculture.

[8] Aii H, Szyper JP, Tamaru CS, Howerton RD, Hopkins KD, Fast AW, Weidenbach RP. Maturation, hatchery, and nursery techniques for chinese catfish, *Clarias fuscus*, in Hawaii. Aquaculture Extension Bulletin 2001; Summer 2001:1–8.

[9] Li G, Deng S, Wang W, Sun J, Wu T, Shi S, Zhu C (2013). Effects of sex steroid hormones on sex differentiation of *Clarias fuscus*. Acta Hydrobiol Sin.

[10] Na-Nakorn U, Brummett RE (2009). Use and exchange of aquatic genetic resources for food and aquaculture: *Clarias* catfish. Rev Aquac.

[11] Young M, Fast AW (1990). Temperature and photoperiod effects on ovarian maturation in the chinese catfish (*Clarias fuscus*: Lacepede). J Aquaculture Tropics.

[12] Young MJA, Fast AW, Olin PG (2010). Induced maturation and spawning of the chinese catfish *Clarias fuscus*. J World Aquacult Soc.

[13] Li N, Bao L, Zhou T, Yuan Z, Liu S, Dunham R, Li Y, Wang K, Xu X, Jin Y (2018). Genome sequence of walking catfish (*Clarias batrachus*) provides insights into terrestrial adaptation. BMC Genomics.

[14] Na-Nakorn U, Kamonrat W, Ngamsiri T. Genetic diversity of walking catfish, *Clarias macrocephalus*, in Thailand and evidence of genetic introgression from introduced farmed *C. gariepinus*. Aquaculture 2004; 240(1–4):145–163.

[15] Kushwaha B, Pandey M, Das P, Joshi CG, Nagpure NS, Kumar R, Kumar D, Agarwal S, Srivastava S, Singh M (2021). The genome of walking catfish *Clarias magur* (Hamilton, 1822) unveils the genetic basis that may have facilitated the development of environmental and terrestrial adaptation systems in air-breathing catfishes. DNA Res.

[16] Chen J, Wang Y, Yue Y, Xia X, Du Q, Chang Z (2009). A novel male-specific DNA sequence in the common carp, *Cyprinus carpio*. Mol Cell Probes.

[17] Karl H, Kuhlmann H, Oetjen K (2002). Transfer of toxaphene and chlordane into farmed rainbow trout, *Oncorhynchus mykiss* (Walbaum) via feed. Aquac Res.

[18] Chen S-L, Li J, Deng S-P, Tian Y-S, Wang Q-Y, Zhuang Z-M, Sha Z-X, Xu J-Y (2007). Isolation of female-specific AFLP markers and molecular identification of genetic sex in half-smooth Tongue Sole (*Cynoglossus semilaevis*). Mar Biotechnol.

[19] Dan C, Mei J, Wang D, Gui J-F (2013). Genetic differentiation and efficient sex-specific marker development of a pair of Y- and X-linked markers in Yellow Catfish. Int J Biol Sci.

[20] Desprez D, MClard C, Philippart JC (1995). Production of a high percentage of male offspring with 17α-ethynylestradiol sex-reversed *Oreochromis aureus* II. Comparative reproductive biology of females and F2 pseudofemales and largescale production of male progeny. Aquaculture.

[21] Goudie CA, Liu Q, Simco BA, Davis KB (1995). Genetic relationship of growth, sex and glucosephosphate isomerase-B phenotypes channel catfish (*Ictalurus punctatus*). Aquaculture.

[22] Liu ZJ, Liu SK, Yao J, Bao LS, Zhang JR, Li Y, Jiang C, Sun LY, Wang RJ, Zhang Y (2016). The channel catfish genome sequence provides insights into the evolution of scale formation in teleosts. Nat Commun.

[23] Gong GR, Dan C, Xiao SJ, Guo WJ, Huang PP, Xiong Y, Wu JJ, He Y, Zhang JC, Li XH et al. Chromosomal-level assembly of yellow catfish genome using third-generation DNA sequencing and Hi-C analysis. Gigascience 2018; 7(11). 10.1093/gigascience/giy120 PMC6228179 30256939

[24] Gao ZJ, You XX, Zhang XH, Chen JM, Xu TF, Huang Y, Lin XQ, Xu JM, Bian C, Shi Q (2021). A chromosome-level genome assembly of the striped catfish (*Pangasianodon hypophthalmus*). Genomics.

[25] Zheng SQ, Shao F, Tao WJ, Liu ZL, Long J, Wang XS, Zhang S, Zhao QY, Carleton KL, Kocher TD (2021). Chromosome-level assembly of southern catfish (*Silurus meridionalis*) provides insights into visual adaptation to nocturnal and benthic lifestyles. Mol Ecol Resour.

[26] Luo J, Wang Z, Lin Z, Yang J (1986). Studies of the karyotypr of *Claris fuscus*. J Fisheries China.

[27] Gong G, Dan C, Xiao S, Guo W, Huang P, Xiong Y, Wu J, He Y, Zhang J, Li X et al. Chromosomal-level assembly of yellow catfish genome using third-generation DNA sequencing and Hi-C analysis. GigaScience 2018; 7:giy120. 10.1093/gigascience/giy120 PMC6228179 30256939

[28] Chen XH, Zhong LQ, Bian C, Xu P, Qiu Y, You XX, Zhang SY, Huang Y, Li J, Wang MH (2016). High-quality genome assembly of channel catfish, *Ictalurus punctatus*. Gigascience.

[29] Bao LS, Tian CX, Liu SK, Zhang Y, Elaswad A, Yuan ZH, Khalil K, Sun FY, Yang YJ, Zhou T (2019). The Y chromosome sequence of the channel catfish suggests novel sex determination mechanisms in teleost fish. BMC Biol.

[30] Dan C, Lin QH, Gong GR, Yang TY, Xiong ST, Xiong Y, Huang PP, Gui JF, Mei J (2018). A novel PDZ domain-containing gene is essential for male sex differentiation and maintenance in yellow catfish (*Pelteobagrus fulvidraco*). Sci Bull.

[31] Zheng SQ, Tao WJ, Yang HW, Kocher TD, Wang ZJ, Peng ZG, Jin L, Pu DY, Zhang YG, Wang DS: Identification of sex chromosome and sex-determining gene of southern catfish (*Silurus meridionalis*) based on XX, XY and YY genome sequencing. Proceedings of the Royal Society B-Biological Sciences 2022; 290(1980): 20222381. 10.1098/rspb.2021.2645 PMC8924754 35291838

[32] Wen M, Pan QW, Jouanno E, Montfort J, Zahm M, Cabau C, Klopp C, Iampietro C, Roques C, Bouchez O *et al*: An ancient truncated duplication of the anti-Mullerian hormone receptor type 2 gene is a potential conserved master sex determinant in the Pangasiidae catfish family. Molecular Ecology Resources 2022; 6: 2411–2428. 10.1111/1755-0998.13620 PMC9555307 35429227

[33] Bakker ST, van de Vrugt HJ, Rooimans MA, Oostra AB, Steltenpool J, Delzenne-Goette E, van der Wal A, van der Valk M, Joenje H, te Riele H (2009). Fancm-deficient mice reveal unique features of fanconi anemia complementation group M. Hum Mol Genet.

[34] Yin H, Ma H, Hussain S, Zhang H, Xie XF, Jiang L, Jiang XH, Iqbal F, Bukhari I, Jiang HW (2019). A homozygous FANCM frameshift pathogenic variant causes male infertility. Genet Sci.

[35] Shan D, Arhin SK, Zhao JZ, Xi HT, Zhang F, Zhu CF, Hu YY. Effects of SLIRP on Sperm Motility and Oxidative Stress. Biomed Research International 2020; 2020:9060356. 10.1155/2020/9060356 PMC7603556 33150185

[36] Wu RC, Jiang M, Beaudet AL, Wu MY (2013). ARID4A and ARID4B regulate male fertility, a functional link to the AR and RB pathways. Proc Natl Acad Sci USA.

[37] Lores P, Coutton C, El Khouri E, Stouvenel L, Givelet M, Thomas L, Rode B, Schmitt A, Louis B, Sakheli Z (2019). Homozygous missense mutation L673P in adenylate kinase 7 (AK7) leads to primary male infertility and multiple morphological anomalies of the flagella but not to primary ciliary dyskinesia. Hum Mol Genet.

[38] Shawki HH, Ishikawa-Yamauchi Y, Kawashima A, Katoh Y, Matsuda M, Al-Soudy A, Minisy FM, Kuno A, Gulibaikelamu X, Hirokawa T (2019). EFCAB2 is a novel calcium-binding protein in mouse testis and sperm. PLoS ONE.

[39] Zheng Z, Zheng H, Yan W (2007). Fank1 is a testis-specific gene encoding a nuclear protein exclusively expressed during the transition from the melotic to the haploid phase of spermatogenesis. Gene Expr Patterns.

[40] Zhang JT, Zhang X, Zhang Y, Zeng WT, Zhao SQ, Liu MX (2019). Normal spermatogenesis in Fank1 (fibronectin type 3 and ankyrin repeat domains 1) mutant mice. Peerj.

[41] MacLean G, Li H, Metzger D, Chambon P, Petkovich M (2007). Apoptotic extinction of germ cells in testes of Cyp26b1 knockout mice. Endocrinology.

[42] Nie DS, Liu Y, Xiang Y (2011). Overexpression a novel zebra fish spermatogenesis-associated gene 17 (SPATA17) induces apoptosis in GC-1 cells. Mol Biol Rep.

[43] Kashimada K, Pelosi E, Chen H, Schlessinger D, Wilhelm D, Koopman P (2011). FOXL2 and BMP2 act cooperatively to regulate Follistatin Gene expression during Ovarian Development. Endocrinology.

[44] Wang Z, Lu L, Gu T, Hou L, Du L, Zhang Y, Zhang Y, Xu Q, Chen G (2021). The effects of FAR1 and TGFBRAP1 on the proliferation and apoptosis of follicular granulosa cells in goose (Anser cygnoides). Gene.

[45] Wu XJ, Williams MJ, Patel PR, Kew KA, Zhu Y. Subfertility and reduced progestin synthesis in Pgrmc2 knockout zebrafish. General and Comparative Endocrinology 2019; 282. 10.1016/j.ygcen.2019.113218 PMC6718323 31301284

[46] Rydze RT, Patton BK, Briley SM, Torralba HS, Gipson G, James R, Rajkovic A, Thompson T, Pangas SA (2021). Deletion of Gremlin-2 alters estrous cyclicity and disrupts female fertility in mice. Biol Reprod.

[47] Jin Y, Yang M, Gao C, Yue W, Liang X, Xie B, Zhu X, Fan S, Li R, Li M (2019). Fbxo30 regulates chromosome segregation of oocyte meiosis. Cell Mol Life Sci.

[48] Zhao BW, Sun SM, Xu K, Li YY, Lei WL, Li L, Liu SL, Ouyang YC, Sun QY, Wang ZB (2021). FBXO34 regulates the G2/M transition and anaphase entry in meiotic oocytes. Front Cell Dev Biology.

[49] Tang DD, Xu C, Geng H, Gao Y, Cheng HR, Ni XQ, He XJ, Cao YX (2020). A novel homozygous mutation in the meiotic gene MSH4 leading to male infertility due to non-obstructive azoospermia. Am J Translational Res.

[50] Okamura E, Tam OH, Posfai E, Li L, Cockburn K, Lee CQE, Garner J, Rossant J (2019). Esrrb function is required for proper primordial germ cell development in presomite stage mouse embryos. Dev Biol.

[51] Rao SSP, Huntley MH, Durand NC, Stamenova EK, Bochkov ID, Robinson JT, Sanborn AL, Machol I, Omer AD, Lander ES (2014). A 3D map of the Human Genome at Kilobase Resolution reveals principles of chromatin looping. Cell.

[52] Yang X, Liu D, Liu F, Wu J, Zou J, Xiao X, Zhao F, Zhu B (2013). HTQC: a fast quality control toolkit for Illumina sequencing data. BMC Bioinformatics.

[53] Chen Y, Chen Y, Shi C, Huang Z, Zhang Y, Li S, Li Y, Ye J, Yu C, Li Z (2017). SOAPnuke: a MapReduce acceleration-supported software for integrated quality control and preprocessing of high-throughput sequencing data. Gigascience.

[54] Xiao C-L, Chen Y, Xie S-Q, Chen K-N, Wang Y, Han Y, Luo F, Xie Z (2017). MECAT: fast mapping, error correction, and de novo assembly for single-molecule sequencing reads. Nat Methods.

[55] Walker BJ, Abeel T, Shea T, Priest M, Abouelliel A, Sakthikumar S, Cuomo CA, Zeng Q, Wortman J, Young SK (2014). Pilon: an Integrated Tool for Comprehensive Microbial variant detection and genome Assembly Improvement. PLoS ONE.

[56] Li H. Aligning sequence reads, clone sequences and assembly contigs with BWA-MEM. arxiv 2013; 00:1–3.

[57] Dudchenko O, Batra SS, Omer AD, Nyquist SK, Hoeger M, Durand NC, Shamim MS, Machol I, Lander ES, Aiden AP (2017). De novo assembly of the Aedes aegypti genome using Hi-C yields chromosome-length scaffolds. Science.

[58] Durand NC, Robinson JT, Shamim MS, Machol I, Mesirov JP, Lander ES, Aiden EL (2016). Juicebox provides a visualization system for Hi-C contact maps with unlimited zoom. Cell Syst.

[59] Simao FA, Waterhouse RM, Ioannidis P, Kriventseva EV, Zdobnov EM (2015). BUSCO: assessing genome assembly and annotation completeness with single-copy orthologs. Bioinformatics.

[60] Jurka J, Kapitonov VV, Pavlicek A, Klonowski P, Kohany O, Walichiewicz J (2005). Repbase update, a database of eukaryotic repetitive elements. Cytogenet Genome Res.

[61] Chen N. Using RepeatMasker to identify repetitive elements in genomic sequences. Current protocols in bioinformatics 2004; Supplement 5:4.10.11–14.10.14. 10.1002/0471250953.bi0410s05 18428725

[62] Abrusan G, Grundmann N, DeMester L, Makalowski W (2009). TEclass-a tool for automated classification of unknown eukaryotic transposable elements. Bioinformatics.

[63] Ou S, Jiang N (2019). LTR_FINDER_parallel: parallelization of LTR_FINDER enabling rapid identification of long terminal repeat retrotransposons. Mob DNA.

[64] Benson G (1999). Tandem repeats finder: a program to analyze DNA sequences. Nucleic Acids Res.

[65] Gertz EM, Yu Y-K, Agarwala R, Schaffer AA, Altschul SF (2006). Composition-based statistics and translated nucleotide searches: improving the TBLASTN module of BLAST. BMC Biol.

[66] Stanke M, Keller O, Gunduz I, Hayes A, Waack S, Morgenstern B (2006). AUGUSTUS: ab initio prediction of alternative transcripts. Nucleic Acids Res.

[67] Stifanic M, Batel R (2007). Genscan for Arabidopsis is a valuable tool for predicting sponge coding sequences. Biologia.

[68] Brueffer C, Saal LH (2016). TopHat-Recondition: a post-processor for TopHat unmapped reads. BMC Bioinformatics.

[69] Campbell MS, Holt C, Moore B, Yandell M. Genome Annotation and Curation Using MAKER and MAKER-P. Current protocols in bioinformatics 2014; Supplement 48:4.11.11–14.11.39. 10.1002/0471250953.bi0411s48 PMC4286374 25501943

[70] Lowe TM, Eddy SR (1997). tRNAscan-SE: a program for improved detection of transfer RNA genes in genomic sequence. Nucleic Acids Res.

[71] Altschul SF, Gish W, Miller W, Myers EW, Lipman DJ (1990). Basic local alignment search tool. J Mol Biol.

[72] Nawrocki EP, Eddy SR (2013). Infernal 1.1: 100-fold faster RNA homology searches. Bioinformatics.

[73] Li L, Stoeckert CJ, Roos DS (2003). OrthoMCL: identification of ortholog groups for eukaryotic genomes. Genome Res.

[74] Stamatakis A (2014). RAxML version 8: a tool for phylogenetic analysis and post-analysis of large phylogenies. Bioinformatics.

[75] Yang Z (2007). PAML 4: phylogenetic analysis by maximum likelihood. Mol Biol Evol.

[76] De Bie T, Cristianini N, Demuth JP, Hahn MW (2006). CAFE: a computational tool for the study of gene family evolution. Bioinformatics.

[77] Delcher AL, Salzberg SL, Phillippy AM. Using MUMmer to identify similar regions in large sequence sets. Current protocols in bioinformatics 2003; Chap. 10:10.13.11–10.13.18. 10.1002/0471250953.bi1003s00 18428693

