# Supplementary material for: A chromosome-level genome assembly of Hong Kong catfish (Clarias fuscus) uncovers a sex-determining region
Source: BMC Genomics. 2023 May 30;24:291. doi: 10.1186/s12864-023-09394-2 (PMC10230808; doi:10.1186/s12864-023-09394-2)
Supplement: Supplementary file 1 — Supplementary Material 1 [file 12864_2023_9394_MOESM1_ESM.docx]

**Table legends**

Supplementary Table S1 Sequencing data for *Clarias fuscus* genome assembly

Supplementary Table S2 Statistics of assembled chromosomes in *Clarias fuscus* genome.

Supplementary Table S3 The BUSCO of *Clarias fuscus* genome assembly and annotation.

Supplementary Table S4 Gene annotations of protein-coding gene in *Clarias fuscus* genome

Supplementary Table S5 Statistical of Repetitive sequences in *Clarias fuscus* genome

Supplementary Table S6 Statistical of transposable element sequences in *Clarias fuscus* genome

Supplementary Table S7 Statistical of non-coding protein genes in *Clarias fuscus* genome

Supplementary Table S8 Statistics of gene family clustering in *Clarias fuscus* genome

Supplementary Table S9 Statistics of unique, expanded and contracted gene family clustering in *Clarias fuscus* genome

Supplementary Table S10 Genes annotation in the sex-linked QTL region of *Clarias fuscus.*

Supplementary Table S1 Sequencing data for *Clarias fuscus* genome assembly

|  | Sequencing libraries | Clean data (Gb) | Clean read (Gb) | Q20 (%) | Q30 (%) | Sequence coverage (×) |
| --- | --- | --- | --- | --- | --- | --- |
| DNA | PacBio | 179.58 | 10,269,296 | - | - | 192.48 |
|  | Hi-C | 107.18 | 725,685,598 | 96.7 | 88.3 | 114.88 |
|  | BGI | 56.85 | 379,011,716 | 95.8 | 87.0 | 60.93 |
|  | Total | 343.61 | - | - | - | 368.29 |

Supplementary Table S2 Statistics of assembled chromosomes in *Clarias fuscus* genome.

| Chromosome | Length(bp) | Contig number | N base (bp) | Gap number | Max contig length (bp) | Min contig length (bp) | Contig N50 (bp) |
| --- | --- | --- | --- | --- | --- | --- | --- |
| Chr1 | 48,393,616 | 20 | 9,500 | 19 | 14,759,191 | 36,234 | 4,950,269 |
| Chr2 | 44,226,111 | 28 | 13,500 | 27 | 10,920,837 | 71,960 | 5,360,070 |
| Chr3 | 42,567,670 | 10 | 4,500 | 9 | 20,987,718 | 40,622 | 11,717,036 |
| Chr4 | 41,622,386 | 10 | 4,500 | 9 | 13,190,150 | 46,594 | 12,411,607 |
| Chr5 | 41,367,455 | 51 | 25,000 | 50 | 13,842,943 | 16,935 | 4,101,265 |
| Chr6 | 40,664,353 | 28 | 13,500 | 27 | 11,196,476 | 27,550 | 5,757,005 |
| Chr7 | 40,308,529 | 8 | 3,500 | 7 | 18,234,350 | 181,347 | 11,862,670 |
| Chr8 | 40,033,569 | 6 | 2,500 | 5 | 26,641,720 | 197,160 | 26,641,720 |
| Chr9 | 39,546,671 | 16 | 7,500 | 15 | 14,624,856 | 36,141 | 13,720,862 |
| Chr10 | 38,214,682 | 32 | 15,500 | 31 | 10,789,849 | 44,797 | 8,386,172 |
| Chr11 | 36,128,006 | 12 | 5,500 | 11 | 14,062,109 | 38,059 | 7,071,909 |
| Chr12 | 35,683,429 | 4 | 1,500 | 3 | 23,069,255 | 96,778 | 23,069,255 |
| Chr13 | 33,445,112 | 7 | 3,000 | 6 | 21,832,678 | 37,518 | 21,832,678 |
| Chr14 | 33,335,127 | 17 | 8,000 | 16 | 8,956,784 | 51,881 | 4,964,331 |
| Chr15 | 33,019,954 | 11 | 5,000 | 10 | 17,819,591 | 46,602 | 17,819,591 |
| Chr16 | 31,575,271 | 14 | 6,500 | 13 | 13,435,711 | 34,259 | 5,144,140 |
| Chr17 | 31,356,693 | 17 | 8,000 | 16 | 10,409,257 | 36,452 | 8,632,194 |
| Chr18 | 31,288,214 | 7 | 3,000 | 6 | 19,627,711 | 50,464 | 19,627,711 |
| Chr19 | 31,016,185 | 9 | 4,000 | 8 | 11,275,328 | 38,889 | 10,383,761 |
| Chr20 | 29,968,065 | 6 | 2,500 | 5 | 14,066,826 | 192,135 | 7,734,019 |
| Chr21 | 29,007,156 | 32 | 15,500 | 31 | 7,089,797 | 23,845 | 3,673,169 |
| Chr22 | 27,809,300 | 27 | 13,000 | 26 | 6,065,294 | 17,098 | 3,550,059 |
| Chr23 | 26,801,903 | 15 | 7,000 | 14 | 10,169,128 | 35,146 | 6,056,520 |
| Chr24 | 26,597,435 | 7 | 3,000 | 6 | 14,102,111 | 68,712 | 14,102,111 |
| Chr25 | 24,068,958 | 9 | 4,000 | 8 | 10,119,204 | 42,368 | 6,404,585 |
| Chr26 | 22,997,812 | 6 | 2,500 | 5 | 18,511,595 | 153,750 | 18,511,595 |
| Chr27 | 18,276,042 | 3 | 1,000 | 2 | 8,148,650 | 3,122,014 | 7,004,378 |
| Chr28 | 14,274,793 | 4 | 1,500 | 3 | 7,487,321 | 61,867 | 7,487,321 |

Supplementary Table S3 The BUSCO of *Clarias fuscus* genome assembly and annotation.

|  | Assembly | Annotation |
| --- | --- | --- |
| Complete BUSCO | 4,270 (93.15 %) | 4,255 (92.82 %) |
| Single-copy BUSCO | 4,104 (89.53 %) | 2,976 (64.92 %) |
| Duplicated BUSCO | 166 (3.62 %) | 1,279 (27.90 %) |
| Fragmented BUSCO | 133 (2.90 %) | 133 (2.90 %) |
| Missing BUSCO | 181 (3.95 %) | 196 (4.28 %) |
| Total BUSCO | 4,584 (100.00 %) | 4,584 (100.00 %) |

Supplementary Table S4 Gene annotations of protein-coding gene in *Clarias fuscus* genome

| Database | Gene annotations (% of all gene) | Transcript annotations (% of all transcript) |
| --- | --- | --- |
| InterPro | 20,083 (86.03%) | 28,555 (88.64 %) |
| GO | 15,269 (65.41%) | 21,738 (67.48 %) |
| KEGG | 21,857 (93.63%) | 30,693 (95.27 %) |
| Swissprot | 20,373 (87.27%) | 28,980 (89.96 %) |
| TrEMBL | 21,768 (93.24%) | 30,569 (94.89 %) |
| NR | 21,973 (94.12%) | 30,819 (95.66 %) |
| Annotated | 22,009 (94.28%) | 30,857 (95.78 %) |
| Unannotated | 1,336 (5.72%) | 1,359 (4.22 %) |
| Total | 23,345 (100%) | 32,216 (100%) |

Supplementary Table S5 Statistical of Repetitive sequences in *Clarias fuscus* genome

| Type | Method | Software | Repeat Size | % of genome |
| --- | --- | --- | --- | --- |
| Tandem repeat | - | Trf | 42,226,621 | 4.52 |
| Transposable element | Homolog | Repeatmasker | 172,110,340 | 18.44 |
|  |  | Proteinmask | 52,960,418 | 5.67 |
|  | De novo | RepeatModeler | 418,410,889 | 44.83 |
|  |  | LTR-FINDER |  |  |
|  |  | RepeatMasker |  |  |
| Total | - | - | 499,350,468 | 53.50 |

Supplementary Table S6 Statistical of transposable element sequences in *Clarias fuscus* genome

|  | Repetitive sequences (% of genome) |
| --- | --- |
| SINE (bp) | 7,472,277 (0.80 %) |
| LINE (bp) | 94,673,725 (10.14 %) |
| LTR (bp) | 189,237,996 (20.27 %) |
| DNA (bp) | 282,143,782 (30.23 %) |
| Unclassified (bp) | 34,396,259 (3.69 %) |
| Total (bp) | 499,350,468 (51.50 %) |

Supplementary Table S7 Statistical of non-coding protein genes in *Clarias fuscus* genome

| Type | | Munber | Total length(bp) | % of genome |
| --- | --- | --- | --- | --- |
| miRNA | | 278 | 23,443 | 0.0025 |
| tRNA | | 12,110 | 904,139 | 0.0957 |
| rRNA | rRNA | 1,672 | 193,082 | 0.0204 |
|  | 18S | 4 | 3,802 | 0.0004 |
|  | 28S | 0 | 0 | 0 |
|  | 5.8S | 2 | 294 | 0 |
|  | 5S | 1,666 | 188,986 | 0.02 |
|  | 8S | 0 | 0 | 0 |
| snRNA | snRNA | 506 | 70,342 | 0.0074 |
|  | CD-box | 120 | 19,389 | 0.0021 |
|  | HACA-box | 14 | 2,548 | 0.0003 |
|  | splicing | 369 | 48,087 | 0.0051 |
|  | scaRNA | 2 | 262 | 0 |

Supplementary Table S8 Statistics of gene family clustering in *Clarias fuscus* genome

| Species | Total genes | Single-copy gene number | Multi-copy gene number | Unique gene number | Other gene number | Cluster gene number | UnCluster gene number | Total family number | Unique family number | Ave. genes per family |
| --- | --- | --- | --- | --- | --- | --- | --- | --- | --- | --- |
| *C. fuscus* | 23,345 | 4,638 | 5,414 | 338 | 11,755 | 22,145 | 1,200 | 15,929 | 60 | 1.39 |
| *L. chalumnae* | 17,953 | 5,534 | 3,085 | 520 | 7,897 | 17,036 | 917 | 13,192 | 113 | 1.29 |
| *L. oculatus* | 18,060 | 5,734 | 2,649 | 222 | 8,984 | 17,589 | 471 | 14,727 | 54 | 1.19 |
| *D. rerio* | 30,980 | 3,528 | 9,501 | 1,739 | 15,668 | 30,436 | 544 | 15,850 | 244 | 1.92 |
| *E. electricus* | 21,836 | 4,283 | 6,322 | 154 | 10,865 | 21,624 | 212 | 15,540 | 34 | 1.39 |
| *A. mexicanus* | 24,265 | 4,236 | 6,675 | 329 | 12,585 | 23,825 | 440 | 16,004 | 80 | 1.49 |
| *C. magur* | 23,712 | 4,826 | 5,242 | 447 | 10,186 | 20,701 | 3,011 | 13,967 | 144 | 1.48 |
| *S. meridionalis* | 21,592 | 4,503 | 5,732 | 260 | 10,706 | 21,201 | 391 | 15,379 | 63 | 1.38 |
| *P. hypophthalmus* | 20,997 | 4,473 | 5,774 | 31 | 9,976 | 20,254 | 743 | 15,121 | 13 | 1.34 |
| *I. punctatus* | 22,027 | 4,433 | 6,056 | 121 | 11,285 | 21,895 | 132 | 15,668 | 30 | 1.40 |
| *P. fulvidraco* | 23,098 | 4,410 | 6,321 | 184 | 11,943 | 22,858 | 240 | 15,623 | 49 | 1.46 |
| *E. lucius* | 24,385 | 4,024 | 7,166 | 542 | 12,094 | 23,826 | 559 | 15,899 | 107 | 1.50 |
| *G. morhua* | 22,863 | 4,562 | 5,834 | 523 | 11,501 | 22,420 | 443 | 15,172 | 107 | 1.48 |
| *O. latipes* | 21,894 | 4,523 | 5,727 | 445 | 10,958 | 21,653 | 241 | 15,252 | 68 | 1.42 |
| *O. niloticus* | 29,092 | 4,263 | 7,098 | 1,664 | 15,642 | 28,667 | 425 | 16,206 | 257 | 1.77 |
| *T. rubripes* | 21,826 | 4,365 | 6,117 | 459 | 10,605 | 21,546 | 280 | 15,074 | 103 | 1.43 |

Supplementary Table S9 Statistics of unique, expanded and contracted gene family clustering in *Clarias fuscus* genome

| No. of gene family clustering | Number of gene | PFAM annotation |
| --- | --- | --- |
| **Unique gene family** | | |
| Cluster332 | 59 | - |
| Cluster1112 | 35 | - |
| Cluster3882 | 24 | DDE_3, HTH_Tnp_Tc3_2 |
| Cluster10686 | 16 | - |
| Cluster15236 | 12 | - |
| Cluster15544 | 11 | - |
| Cluster15556 | 11 | - |
| Cluster15875 | 10 | - |
| Cluster16109 | 9 | CENP-B_N, DDE_1, HTH_Tnp_Tc5 |
| Cluster16122 | 9 | - |
| Cluster16141 | 9 | - |
| Cluster16687 | 7 | - |
| Cluster17246 | 5 | - |
| Cluster17253 | 5 | FYVE, Myotub-related |
| Cluster17262 | 5 | - |
| Cluster17272 | 5 | - |
| Cluster17676 | 4 | - |
| Cluster17693 | 4 | - |
| Cluster17702 | 4 | ANF_receptor, Lig_chan, Lig_chan-Glu_bd |
| Cluster17729 | 4 | RBP_receptor |
| Cluster17750 | 4 | HD, SAM_1, SAM_2 |
| Cluster17756 | 4 | - |
| Cluster18280 | 3 | - |
| Cluster18324 | 3 | - |
| Cluster18330 | 3 | - |
| Cluster18338 | 3 | - |
| Cluster18353 | 3 | PMP22_Claudin |
| Cluster18389 | 3 | - |
| Cluster19018 | 2 | PRY, SPRY, zf-B_box, zf-C3HC4_4 |
| Cluster19019 | 2 | - |
| Cluster19020 | 2 | I-set, Ig_3 |
| Cluster19040 | 2 | - |
| Cluster19044 | 2 | - |
| Cluster19051 | 2 | - |
| Cluster19052 | 2 | - |
| Cluster19057 | 2 | - |
| Cluster19071 | 2 | MBOAT |
| Cluster19072 | 2 | MBOAT |
| Cluster19075 | 2 | EGF_2, RHS_repeat, Ten_N, Tox-GHH |
| Cluster19101 | 2 | Ank_2, FERM_f0, PDZ, SAM_1, SH3_2 |
| Cluster19118 | 2 | - |
| Cluster19149 | 2 | - |
| Cluster19165 | 2 | - |
| Cluster19205 | 2 | SCA7 |
| Cluster19206 | 2 | - |
| Cluster19279 | 2 | Sulfotransfer_1 |
| Cluster19358 | 2 | - |
| Cluster19384 | 2 | - |
| Cluster19386 | 2 | - |
| Cluster19389 | 2 | Dynamin_M, Dynamin_N, GED, PH |
| Cluster19447 | 2 | I-set, Ig_3, Y_phosphatase, fn3 |
| Cluster19480 | 2 | - |
| Cluster19484 | 2 | - |
| Cluster19506 | 2 | - |
| Cluster19535 | 2 | Serpin |
| Cluster19561 | 2 | - |
| Cluster19600 | 2 | - |
| Cluster19712 | 2 | Rhodanese, UCH, USP8_dimer |
| Cluster19794 | 2 | Neur_chan_LBD, Neur_chan_memb |
| Cluster19810 | 2 | - |
| **expanded gene family** | | |
| Cluster0 | 37 | PRY, SPRY, zf-B_box, zf-C3HC4_4 |
| Cluster1 | 35 | FISNA, LRR_6, NACHT, PRY, SPRY |
| Cluster12 | 38 | C2-set_2, I-set, Ig_2, Ig_3, V-set |
| Cluster17 | 17 | 7tm_1 |
| Cluster19 | 18 | Histone, Histone_H2A_C |
| Cluster21 | 13 | Histone |
| Cluster22 | 14 | Histone |
| Cluster35 | 11 | Tubulin, Tubulin_C |
| Cluster43 | 12 | CENP-T_C, Histone |
| Cluster46 | 15 | IL8 |
| Cluster48 | 20 | Linker_histone |
| Cluster50 | 3 | 7tm_4 |
| Cluster51 | 22 | C1-set, V-set |
| Cluster58 | 17 | Endonuclease_NS |
| Cluster67 | 18 | 7tm_4 |
| Cluster82 | 12 | HSP70 |
| Cluster85 | 16 | Glyco_transf_6 |
| Cluster89 | 13 | V-set |
| Cluster96 | 7 | Dynamin_N, RHD3 |
| Cluster108 | 5 | DDE_Tnp_1_7, Tnp_zf-ribbon_2 |
| Cluster110 | 9 | Ferritin |
| Cluster113 | 12 | ENTH |
| Cluster116 | 5 | IIGP |
| Cluster123 | 12 | UPAR_LY6 |
| Cluster124 | 32 | C1-set, V-set |
| Cluster130 | 7 | Cation_ATPase, Cation_ATPase_C, Cation_ATPase_N, E1-E2_ATPase, HAD, Hydrolase, Hydrolase_3 |
| Cluster139 | 7 | Dimer_Tnp_hAT |
| Cluster140 | 8 | EF-hand_7, EF-hand_8, Ins145_P3_rec, Ion_trans, MIR, RIH_assoc, RR_TM4-6, RYDR_ITPR, RyR, SPRY |
| Cluster149 | 3 | DUF4371, Dimer_Tnp_hAT |
| Cluster155 | 6 | AA_permease, SLC12 |
| Cluster163 | 8 | Astacin, CUB |
| Cluster168 | 9 | UDPGT |
| Cluster171 | 6 | G-alpha |
| Cluster175 | 14 | 7tm_1 |
| Cluster176 | 6 | IL8 |
| Cluster188 | 6 | XK-related |
| Cluster193 | 6 | C8, F5_F8_type_C, Mucin2_WxxW, TIL, VWC, VWD |
| Cluster200 | 5 | Neur_chan_LBD, Neur_chan_memb |
| Cluster203 | 27 | V-set |
| Cluster210 | 5 | C1_1, C2, DUF1041, Membr_traf_MHD |
| Cluster214 | 38 | DDE_3, DUF4817 |
| Cluster237 | 5 | zf-B_box, zf-RING_UBOX |
| Cluster242 | 6 | RVT_1, rve |
| Cluster244 | 5 | AIG1, MMR_HSR1, PRY, SPRY, Septin, fn3 |
| Cluster265 | 5 | CH, EFhand_Ca_insen, Spectrin |
| Cluster266 | 5 | 7tm_1 |
| Cluster277 | 9 | Neurexophilin |
| Cluster303 | 5 | CRAL_TRIO_2, I-set, PH, Pkinase, RhoGEF, SH3-RhoG_link, SH3_1, Spectrin, fn3 |
| Cluster305 | 25 | Globin |
| Cluster306 | 4 | Homeobox, Pou |
| Cluster321 | 6 | Hemopexin, PG_binding_1, Peptidase_M10 |
| Cluster341 | 21 | - |
| Cluster368 | 4 | zf-C2H2 |
| Cluster419 | 15 | RVT_1 |
| Cluster423 | 4 | AAA_5, AAA_6, AAA_7, AAA_8, AAA_9, DHC_N1, DHC_N2, Dynein_heavy, MT |
| Cluster426 | 4 | GDI |
| Cluster430 | 4 | PH_9, Sec7 |
| Cluster437 | 5 | adh_short |
| Cluster446 | 4 | rve |
| Cluster463 | 4 | Pentaxin |
| Cluster473 | 7 | p450 |
| Cluster478 | 4 | Collagen |
| Cluster484 | 5 | Ank_2, Pkinase, Ribonuc_2-5A |
| Cluster486 | 7 | Pentaxin |
| Cluster487 | 4 | ubiquitin |
| Cluster492 | 4 | HMG_box |
| Cluster505 | 4 | CLZ, Ion_trans, cNMP_binding |
| Cluster523 | 4 | IRK |
| Cluster526 | 4 | - |
| Cluster560 | 4 | Drf_DAD, Drf_FH3, Drf_GBD, FH2 |
| Cluster570 | 4 | Pyridoxal_deC |
| Cluster574 | 4 | RAMP4 |
| Cluster623 | 4 | ABC2_membrane, ABC_tran |
| Cluster626 | 4 | Ig_3 |
| Cluster688 | 4 | Fumble |
| Cluster704 | 2 | Hepcidin |
| Cluster719 | 5 | I-set, Ig_2, Ig_3, TIR, ig |
| Cluster727 | 4 | PMP22_Claudin |
| Cluster735 | 3 | BAAT_C, Bile_Hydr_Trans |
| Cluster742 | 4 | 7tm_1 |
| Cluster764 | 3 | Methyltransf_11 |
| Cluster782 | 3 | 7tm_1 |
| Cluster790 | 4 | TAFA |
| Cluster801 | 4 | Transglut_C, Transglut_N, Transglut_core |
| Cluster812 | 6 | 7tm_1 |
| Cluster848 | 7 | Pkinase |
| Cluster853 | 3 | ANF_receptor, Guanylate_cyc, HNOBA, Pkinase_Tyr |
| Cluster872 | 4 | Acylphosphatase |
| Cluster932 | 3 | EGF, FXa_inhibition, Gla, Trypsin |
| Cluster935 | 3 | 7tm_1 |
| Cluster976 | 4 | Furin-like, GF_recep_IV, Pkinase_Tyr, Recep_L_domain |
| Cluster978 | 3 | EF-hand_1, EF-hand_5, EF-hand_6, EF-hand_8, Mito_carr |
| Cluster979 | 5 | Ependymin |
| Cluster1016 | 4 | PKI |
| Cluster1038 | 4 | DUF3736 |
| Cluster1040 | 4 | ADK, Dpy-30 |
| Cluster1054 | 3 | CD225 |
| Cluster1075 | 3 | DUF4749, PDZ |
| Cluster1134 | 3 | Ig_2 |
| Cluster1140 | 3 | EGF_CA, G2F, I-set, Ig_3, TSP_1 |
| Cluster1141 | 3 | G_glu_transpept |
| Cluster1145 | 11 | CDI |
| Cluster1154 | 3 | DEP, RasGEF, RasGEF_N |
| Cluster1163 | 4 | EGF, I-set, Neuregulin |
| Cluster1166 | 8 | Crystall |
| Cluster1169 | 3 | Na_H_Exchanger |
| Cluster1176 | 3 | DDE_Tnp_4 |
| Cluster1178 | 4 | ERAP1_C, Peptidase_M1 |
| Cluster1179 | 3 | Sulfotransfer_2 |
| Cluster1198 | 3 | KH_1 |
| Cluster1210 | 4 | MYT1, zf-C2HC |
| Cluster1222 | 4 | GPS, Gal_Lectin, Lectin_C, PKD_channel, PLAT, REJ |
| Cluster1240 | 2 | RRM_1 |
| Cluster1272 | 2 | An_peroxidase |
| Cluster1275 | 11 | V1R |
| Cluster1276 | 3 | WD40 |
| Cluster1306 | 3 | ACT_7 |
| Cluster1316 | 5 | SBF |
| Cluster1319 | 3 | DUF4149 |
| Cluster1342 | 3 | RabGAP-TBC |
| Cluster1343 | 3 | wnt |
| Cluster1379 | 3 | ATP-synt_S1 |
| Cluster1428 | 2 | PI-PLC-X |
| Cluster1430 | 3 | AstE_AspA |
| Cluster1432 | 12 | Tnp_22_dsRBD, Transposase_22 |
| Cluster1485 | 3 | Cnn_1N, DUF1220 |
| Cluster1513 | 3 | DUF4685, PDZ |
| Cluster1526 | 4 | PH, SH2 |
| Cluster1533 | 3 | ARID |
| Cluster1557 | 4 | Ax_dynein_light |
| Cluster1592 | 3 | Arrestin_C, Arrestin_N |
| Cluster1659 | 3 | Shisa |
| Cluster1694 | 3 | L_HMGIC_fpl |
| Cluster1859 | 3 | LSR, V-set |
| Cluster1866 | 3 | Bromodomain, DDT, MBD, PHD, WHIM1, WSD |
| Cluster1884 | 3 | Tetraspannin |
| Cluster2029 | 3 | LRR_9, Ubiquitin_2 |
| Cluster2053 | 2 | BTG |
| Cluster2055 | 3 | zf-C2H2_jaz |
| Cluster2064 | 3 | Lipocalin |
| Cluster2183 | 3 | Kunitz_BPTI |
| Cluster2220 | 3 | TNF |
| Cluster2306 | 3 | Amidase |
| Cluster2337 | 8 | 7tm_1 |
| Cluster2377 | 3 | UPF0220 |
| Cluster2431 | 3 | ANF_receptor, Lig_chan, Lig_chan-Glu_bd, SBP_bac_3 |
| Cluster2443 | 3 | Gal-bind_lectin |
| Cluster2444 | 4 | CRAL_TRIO_2, RasGAP |
| Cluster2577 | 4 | PARP, WWE |
| Cluster2776 | 3 | ASH, Hydin_ADK, Motile_Sperm, PapD-like |
| Cluster2845 | 2 | I-set |
| Cluster3056 | 3 | TLD |
| Cluster3123 | 3 | ACBP, GOLD_2 |
| Cluster3208 | 2 | 7tm_1 |
| Cluster3241 | 2 | LRR_8 |
| Cluster3275 | 2 | EF-hand_8 |
| Cluster3358 | 5 | GSHPx |
| Cluster3491 | 2 | Jun, bZIP_1 |
| Cluster3498 | 3 | Ca_hom_mod |
| Cluster3513 | 2 | - |
| Cluster3611 | 2 | 7tm_1 |
| Cluster3664 | 2 | Asp-B-Hydro_N |
| Cluster3740 | 4 | Ca_hom_mod |
| Cluster3760 | 3 | Phospholip_A2_1 |
| Cluster3761 | 2 | CNRIP1 |
| Cluster3845 | 2 | Collagen |
| Cluster3846 | 12 | BTG |
| Cluster3847 | 2 | VWA, fn3 |
| Cluster3898 | 2 | Peptidase_M16, Peptidase_M16_C |
| Cluster3908 | 2 | RRM_1, zf-CCHC |
| Cluster4008 | 2 | Steroid_dh |
| Cluster4016 | 2 | PRY, SPRY, zf-B_box, zf-C3HC4, zf-C3HC4_2, zf-C3HC4_4, zf-RING_UBOX |
| Cluster4020 | 3 | HMG_box, SOXp |
| Cluster4066 | 2 | Phospholip_A2_1 |
| Cluster4096 | 7 | - |
| Cluster4109 | 2 | Aminotran_1_2 |
| Cluster4114 | 2 | PID, WW |
| Cluster4148 | 2 | SecE |
| Cluster4164 | 2 | ATAD4 |
| Cluster4186 | 3 | IDO |
| Cluster4227 | 4 | SEA |
| Cluster4248 | 2 | FHA, Kinesin, PX |
| Cluster4268 | 2 | Enkurin |
| Cluster4289 | 2 | SHIPPO-rpt |
| Cluster4311 | 3 | MORN |
| Cluster4337 | 2 | LRR_8, PDZ |
| Cluster4338 | 3 | C1-set, V-set |
| Cluster4368 | 2 | DUF4553 |
| Cluster4397 | 2 | HLH |
| Cluster4409 | 2 | DAGAT |
| Cluster4422 | 5 | MFS_1 |
| Cluster4425 | 2 | IBR |
| Cluster4436 | 2 | TMEM100 |
| Cluster4443 | 2 | FAM163 |
| Cluster4486 | 2 | zf-C2H2_jaz |
| Cluster4488 | 2 | Orn_Arg_deC_N, Orn_DAP_Arg_deC |
| Cluster4583 | 2 | MAP2_projctn, Tubulin-binding |
| Cluster4611 | 2 | Hormone_recep |
| Cluster4630 | 2 | WD40 |
| Cluster4631 | 2 | Sod_Cu |
| Cluster4684 | 2 | Asp_protease_2, PNMA, RVT_1, rve |
| Cluster4724 | 2 | ADK, FAP206 |
| Cluster4782 | 2 | Tubulin-binding |
| Cluster4788 | 2 | Nucleoporin_C, Nucleoporin_N |
| Cluster4814 | 2 | Methyltransf_11, Methyltransf_25 |
| Cluster4822 | 3 | Peptidase_M28 |
| Cluster4836 | 2 | Scs3p |
| Cluster4853 | 3 | EGF, F5_F8_type_C, Laminin_G_2 |
| Cluster4858 | 3 | DSL, EGF, MNNL, hEGF |
| Cluster4877 | 3 | Carb_anhydrase |
| Cluster4907 | 2 | PH |
| Cluster4917 | 3 | Cadherin |
| Cluster4918 | 3 | Cation_efflux |
| Cluster4940 | 2 | RabGAP-TBC |
| Cluster4965 | 2 | DEAD, Drc1-Sld2, Helicase_C, zf-CCHC |
| Cluster5002 | 2 | DUF1115, RWD |
| Cluster5010 | 2 | OFCC1 |
| Cluster5011 | 2 | Ribosomal_L44 |
| Cluster5018 | 3 | - |
| Cluster5020 | 3 | Pkinase |
| Cluster5050 | 2 | PH |
| Cluster5070 | 2 | JCAD |
| Cluster5179 | 2 | CDC73_C, CDC73_N |
| Cluster5180 | 2 | FAM194 |
| Cluster5187 | 2 | adh_short, adh_short_C2 |
| Cluster5199 | 2 | zf-B_box |
| Cluster5210 | 2 | TPH |
| Cluster5238 | 2 | F-box, F-box-like, FBA |
| Cluster5252 | 3 | tRNA-synt_1b, tRNA_bind |
| Cluster5299 | 2 | Leuk-A4-hydro_C, Peptidase_M1 |
| Cluster5311 | 2 | DS |
| Cluster5317 | 2 | - |
| Cluster5346 | 2 | Forkhead |
| Cluster5347 | 2 | Arf |
| Cluster5350 | 2 | Phospho_p8 |
| Cluster5391 | 2 | Ig_2, Ig_3, TIR |
| Cluster5396 | 2 | Thioredoxin_6, Thioredoxin_8 |
| Cluster5432 | 3 | KASH_CCD |
| Cluster5435 | 2 | Homeobox, zf-C2H2, zf-met |
| Cluster5445 | 3 | DALR_1, tRNA-synt_1d |
| Cluster5458 | 2 | InaF-motif |
| Cluster5474 | 3 | OCD_Mu_crystall |
| Cluster5481 | 2 | GRIM-19, YjeF_N |
| Cluster5482 | 3 | CTF_NFI, MH1, NfI_DNAbd_pre-N |
| Cluster5483 | 2 | Ank_2, Ank_4, Ank_5 |
| Cluster5495 | 2 | zf-3CxxC |
| Cluster5497 | 2 | HEAT_2, PC_rep |
| Cluster5500 | 3 | 2OG-FeII_Oxy, DIOX_N |
| Cluster5516 | 2 | IRS, PH |
| Cluster5529 | 2 | Glyco_transf_22 |
| Cluster5551 | 2 | - |
| Cluster5558 | 2 | C1_1, SH3_1, SH3_2, SH3_9, STAC2_u1 |
| Cluster5560 | 2 | SH3_1, Ysc84 |
| Cluster5574 | 3 | TMEM238 |
| Cluster5583 | 2 | RBD-FIP |
| Cluster5600 | 2 | C2, EF-hand_4, INTAP, PH_13, RhoGEF, SH3_1, SH3_9 |
| Cluster5601 | 3 | - |
| Cluster5622 | 2 | CH, DUF3585, LIM |
| Cluster5657 | 2 | Acyltransferase |
| Cluster5712 | 2 | TPH |
| Cluster5736 | 2 | DNA_pol_A_exo1, Mut7-C |
| Cluster5754 | 2 | - |
| Cluster5807 | 2 | LCAT |
| Cluster5854 | 3 | WD40 |
| Cluster5863 | 2 | Misat_Tub_SegII, Tubulin_3 |
| Cluster5880 | 2 | Chorein_N |
| Cluster5923 | 3 | Methyltransf_11, Methyltransf_25 |
| Cluster5932 | 2 | RRN3 |
| Cluster5953 | 2 | ARF7EP_C, THAP |
| Cluster5967 | 2 | adh_short |
| Cluster5979 | 3 | MAD |
| Cluster5988 | 2 | OST-HTH, TUDOR |
| Cluster5993 | 2 | AKAP2_C, Paralemmin |
| Cluster6025 | 2 | I-set, Ig_3 |
| Cluster6035 | 3 | B3_4, B5 |
| Cluster6085 | 2 | TPR_16, TPR_8 |
| Cluster6087 | 3 | PP2C |
| Cluster6137 | 2 | HSF_DNA-bind, Vert_HS_TF |
| Cluster6149 | 2 | 7tm_1 |
| Cluster6169 | 2 | MOR2-PAG1_C, MOR2-PAG1_N, MOR2-PAG1_mid |
| Cluster6228 | 2 | Ank, Ank_2, Ank_4, MORN, zf-MYND |
| Cluster6230 | 2 | L27_2, PDZ |
| Cluster6322 | 2 | Myb_DNA-bind_5 |
| Cluster6348 | 2 | PHD |
| Cluster6370 | 3 | Guanylate_kin, LRR_4, LRR_6, LRR_9 |
| Cluster6417 | 2 | SH3_1 |
| Cluster6471 | 2 | Xin |
| Cluster6495 | 2 | C8, F5_F8_type_C, Ldl_recept_a, Pacifastin_I, TIL, TSP_1, VWC, VWD |
| Cluster6524 | 2 | DUF3384, Rap_GAP, Tuberin |
| Cluster6533 | 2 | Methyltr_RsmB-F |
| Cluster6537 | 2 | Connexin, Connexin50 |
| Cluster6632 | 6 | - |
| Cluster6719 | 2 | 40S_S4_C, KOW, RS4NT, Ribosomal_S4e, S4 |
| Cluster6733 | 2 | PTE |
| Cluster6734 | 2 | WD40 |
| Cluster6868 | 2 | Band_7, Band_7_C |
| Cluster6869 | 2 | NAD_binding_8, Prenylcys_lyase |
| Cluster6968 | 2 | - |
| Cluster7117 | 2 | GCS |
| Cluster7124 | 2 | - |
| Cluster7130 | 2 | Acyltransf_C, Acyltransferase |
| Cluster7143 | 2 | - |
| Cluster7150 | 2 | DUF4589 |
| Cluster7190 | 2 | Esterase |
| Cluster7198 | 2 | HORMA |
| Cluster7219 | 2 | C2, DUF3498, RasGAP |
| Cluster7248 | 2 | Abhydrolase_1, Hydrolase_4 |
| Cluster7337 | 2 | - |
| Cluster7352 | 2 | I-set, fn3, ig |
| Cluster7426 | 2 | HNH_3, zf-MYND |
| Cluster7465 | 2 | Mtf2_C, PHD |
| Cluster7539 | 2 | V-set |
| Cluster7552 | 2 | UCH, zf-UBP |
| Cluster7732 | 2 | Ribosomal_L19 |
| Cluster8095 | 2 | I-set, IQ, Ig_2, Ig_3, PH, Pkinase, RhoGEF, fn3, ig |
| Cluster8192 | 2 | - |
| Cluster8218 | 2 | OCC1 |
| Cluster8692 | 2 | zf-DBF |
| Cluster9241 | 2 | EF-hand_1, EF-hand_5, EF-hand_7 |
| Cluster9481 | 2 | MBD, zf-CXXC |
| Cluster9851 | 2 | Lipase, PLAT |
| Cluster9940 | 2 | 7tm_2, HRM |
| Cluster9959 | 2 | Ig_3, TIR |
| Cluster9971 | 2 | PLA2G12 |
| Cluster10151 | 2 | I-set, Spectrin |
| Cluster10400 | 2 | TC1 |
| Cluster10670 | 2 | Acyl-CoA_dh_1, Acyl-CoA_dh_M |
| Cluster10888 | 2 | Cys_knot, IGFBP, TSP_1 |
| Cluster11536 | 2 | C2 |
| Cluster11537 | 2 | IF-2B |
| Cluster12195 | 2 | Laminin_EGF, Laminin_N |
| Cluster12556 | 2 | M20_dimer, Peptidase_M20 |
| Cluster12557 | 2 | TPR_19, WD40 |
| Cluster13061 | 2 | BetaGal_dom4_5, Glyco_hydro_35 |
| Cluster13282 | 2 | - |
| Cluster13522 | 2 | 7tm_1 |
| Cluster14075 | 2 | ATAD4 |
| Cluster14100 | 2 | DUF4731 |
| Cluster14200 | 2 | - |
| Cluster14427 | 2 | IQ, LRR_4, LRR_9 |
| Cluster14534 | 2 | Motilin_assoc |
| Cluster14856 | 3 | Cadherin, Cadherin_2, Cadherin_C_2 |
| Cluster15112 | 2 | DUF1126, EF-hand_7 |
| Cluster15619 | 2 | RVT_1 |
| Cluster15847 | 6 | LLGL, Lgl_C, Synaptobrevin, WD40 |
| Cluster16142 | 3 | EGF_CA, FXa_inhibition, Ldl_recept_a, Ldl_recept_b, cEGF |
| Cluster16668 | 2 | IQ_SEC7_PH, Sec7 |
| Cluster16684 | 2 | NYD-SP12_N |
| Cluster16985 | 2 | Pkinase |
| Cluster16989 | 2 | WD40 |
| Cluster17247 | 3 | RVT_1 |
| Cluster17720 | 2 | DUF4782, GRAM |
| Cluster17762 | 2 | BRCT, PADR1, PARP, PARP_reg, WGR, zf-PARP |
| **contracted gene family** | | |
| Cluster2 | 7 | 7tm_3, ANF_receptor, NCD3G |
| Cluster5 | 7 | Crystall |
| Cluster6 | 22 | Lectin_C, Trypsin |
| Cluster7 | 12 | Ig_2, SRCR, ig |
| Cluster10 | 10 | C1-set, MHC_I |
| Cluster14 | 11 | Lectin_C |
| Cluster27 | 4 | I-set, Ig_2, Ig_3, V-set |
| Cluster32 | 3 | Myosin_N, Myosin_head, Myosin_tail_1 |
| Cluster34 | 5 | C2-set_2, PRY, SPRY, V-set |
| Cluster36 | 5 | V-set |
| Cluster44 | 7 | PLAC8 |
| Cluster55 | 5 | Globin |
| Cluster57 | 7 | Ion_trans, Na_trans_assoc |
| Cluster62 | 6 | CD20 |
| Cluster65 | 4 | LRR_6, PRY, SPRY |
| Cluster69 | 0 | Cadherin, Cadherin_2, Cadherin_C_2 |
| Cluster71 | 5 | Cation_ATPase, Cation_ATPase_C, Cation_ATPase_N, E1-E2_ATPase, Hydrolase |
| Cluster86 | 2 | Exo_endo_phos |
| Cluster87 | 5 | PDEase_I |
| Cluster90 | 1 | p450 |
| Cluster93 | 3 | CaMKII_AD, Pkinase |
| Cluster97 | 3 | C1-set, MHC_II_beta |
| Cluster98 | 2 | Zona_pellucida |
| Cluster105 | 3 | I-set, Ig_3, V-set |
| Cluster109 | 5 | COLFI, Collagen, Laminin_G_3 |
| Cluster119 | 4 | WD40 |
| Cluster122 | 5 | 7tm_2, GAIN, GPS, Gal_Lectin, HRM, Latrophilin, OLF |
| Cluster132 | 3 | C1-set, MHC_II_alpha |
| Cluster133 | 7 | 7tm_1 |
| Cluster134 | 2 | Astacin |
| Cluster146 | 5 | GTP_EFTU, GTP_EFTU_D2, GTP_EFTU_D3 |
| Cluster152 | 5 | PH, Sec7 |
| Cluster154 | 0 | RVP, Retrotrans_gag, zf-CCHC |
| Cluster162 | 2 | AMP-binding, AMP-binding_C |
| Cluster166 | 1 | TNFR_c6 |
| Cluster172 | 4 | PSI, Plexin_cytopl, Sema, TIG |
| Cluster174 | 5 | Sushi |
| Cluster180 | 1 | Het-C, VWA_2 |
| Cluster190 | 2 | Mesothelin |
| Cluster191 | 4 | Bromodomain, HSR, PHD, SAND |
| Cluster192 | 4 | Fox-1_C, RRM_1 |
| Cluster197 | 4 | ArgoL1, ArgoL2, ArgoMid, ArgoN, PAZ, Piwi |
| Cluster206 | 1 | EF-hand_1 |
| Cluster209 | 0 | PRY, SPRY, zf-B_box, zf-C3HC4, zf-C3HC4_4, zf-RING_UBOX |
| Cluster223 | 1 | ADH_N, ADH_zinc_N |
| Cluster224 | 0 | ART |
| Cluster225 | 3 | V-set |
| Cluster239 | 3 | Pkinase |
| Cluster248 | 2 | IQ, Myosin_N, Myosin_head, Myosin_tail_1 |
| Cluster250 | 2 | C2-set_2, I-set, Ig_3, V-set, ig |
| Cluster252 | 2 | Ras |
| Cluster255 | 2 | TAFA |
| Cluster272 | 1 | RVT_1, SCAN, rve, zf-CCHC, zf-H2C2 |
| Cluster273 | 2 | PARP, WWE |
| Cluster274 | 2 | Lectin_C |
| Cluster275 | 1 | Apolipoprotein |
| Cluster276 | 3 | Lectin_C |
| Cluster284 | 2 | V-set |
| Cluster285 | 2 | I-set, fn3 |
| Cluster293 | 3 | Ins145_P3_rec, Ion_trans, MIR, RIH_assoc, RYDR_ITPR |
| Cluster298 | 3 | Homeobox, TF_Otx |
| Cluster299 | 3 | Pkinase |
| Cluster300 | 3 | CUT, Homeobox |
| Cluster307 | 4 | VOMI |
| Cluster317 | 3 | Pribosyltran |
| Cluster326 | 2 | FG-GAP, Integrin_alpha, Integrin_alpha2, VWA |
| Cluster327 | 1 | TEA |
| Cluster333 | 3 | DUF1899, Trimer_CC, WD40, WD40_4 |
| Cluster340 | 3 | 7tm_1 |
| Cluster349 | 2 | IP_trans |
| Cluster367 | 3 | Ca_chan_IQ, GPHH, Ion_trans |
| Cluster369 | 3 | PP2C, PP2C_C |
| Cluster371 | 3 | A2M, A2M_N, A2M_N_2, A2M_comp, A2M_recep, Thiol-ester_cl |
| Cluster387 | 3 | Neur_chan_LBD, Neur_chan_memb |
| Cluster400 | 3 | EF-hand_1, EF-hand_6, EF-hand_7, EF-hand_8 |
| Cluster403 | 2 | - |
| Cluster407 | 3 | UQ_con |
| Cluster411 | 3 | IRK |
| Cluster412 | 3 | Homeobox, TRAM_LAG1_CLN8 |
| Cluster427 | 2 | EGF, EGF_CA, FXa_inhibition, Ldl_recept_a, Ldl_recept_b, cEGF |
| Cluster439 | 2 | Sarcoglycan_1 |
| Cluster440 | 2 | MFS_1 |
| Cluster442 | 2 | CBX7_C, Chromo |
| Cluster445 | 4 | Tetraspannin |
| Cluster447 | 2 | Hormone_recep, zf-C4 |
| Cluster451 | 1 | Yippee-Mis18 |
| Cluster454 | 2 | Homeobox_KN, Meis_PKNOX_N |
| Cluster456 | 1 | Sugar_tr |
| Cluster472 | 2 | Med26, TFIIS_C, TFIIS_M |
| Cluster476 | 1 | Bax1-I |
| Cluster497 | 2 | Pkinase |
| Cluster502 | 2 | Rad60-SLD |
| Cluster506 | 2 | UCH |
| Cluster522 | 2 | MH1, MH2 |
| Cluster534 | 1 | ERAP1_C, Peptidase_M1 |
| Cluster536 | 2 | RRM_1 |
| Cluster538 | 2 | A1_Propeptide, Asp |
| Cluster544 | 2 | Homeobox, OAR |
| Cluster546 | 2 | Cadherin_3, Calx-beta |
| Cluster557 | 2 | BTB_2, DUF3504 |
| Cluster566 | 1 | Cofilin_ADF |
| Cluster603 | 2 | Pkinase |
| Cluster615 | 1 | WD40 |
| Cluster646 | 2 | Sema, ig |
| Cluster648 | 1 | Laminin_EGF, Laminin_N |
| Cluster649 | 2 | ANF_receptor, Lig_chan, Lig_chan-Glu_bd |
| Cluster650 | 2 | Septin |
| Cluster651 | 2 | Na_Pi_cotrans |
| Cluster654 | 2 | Gtr1_RagA |
| Cluster657 | 1 | Cyclin_C, Cyclin_N |
| Cluster659 | 2 | FERM_M, IQ, MYO10_CC, MyTH4, Myosin_head, PH, RA |
| Cluster671 | 1 | Cystatin |
| Cluster680 | 1 | SNAP-25 |
| Cluster706 | 2 | HlyIII |
| Cluster715 | 2 | C2, FerA, FerB, FerI, Ferlin_C |
| Cluster716 | 1 | Aminotran_1_2 |
| Cluster738 | 1 | Kelch_2, Kelch_3, Kelch_4, Kelch_5 |
| Cluster739 | 2 | KH_1, Quaking_NLS, STAR_dimer |
| Cluster743 | 1 | bZIP_Maf |
| Cluster745 | 1 | Pyrophosphatase |
| Cluster751 | 1 | DIL, IQ, Myosin_head |
| Cluster761 | 2 | Clat_adaptor_s |
| Cluster762 | 2 | Arrestin_C, Arrestin_N |
| Cluster768 | 2 | Gly_acyl_tr_C, Gly_acyl_tr_N |
| Cluster780 | 2 | HATPase_c, HSP90 |
| Cluster785 | 2 | Glyco_hydro_15 |
| Cluster793 | 1 | SNF |
| Cluster797 | 2 | I-set, Ig_2, ig |
| Cluster830 | 2 | Pkinase, Pkinase_C |
| Cluster832 | 2 | Collagen, VWA |
| Cluster844 | 0 | Trypsin |
| Cluster855 | 2 | CBS, Voltage_CLC |
| Cluster877 | 2 | AAA, CH |
| Cluster901 | 2 | His_Phos_1 |
| Cluster905 | 1 | C1-set, C2-set_2, I-set, V-set |
| Cluster930 | 2 | Actin |
| Cluster946 | 1 | ER_lumen_recept |
| Cluster957 | 1 | C2, FYVE_2 |
| Cluster961 | 1 | PAP2 |
| Cluster997 | 1 | HD, SAM_1, SAM_2 |
| Cluster1012 | 1 | Rib_hydrolayse |
| Cluster1021 | 2 | Ins134_P3_kin |
| Cluster1027 | 1 | Sulfotransfer_1 |
| Cluster1064 | 1 | - |
| Cluster1066 | 1 | Tetraspannin |
| Cluster1071 | 1 | Pkinase |
| Cluster1091 | 1 | C2-set_2, I-set, Ig_3, fn3, ig |
| Cluster1095 | 1 | UQ_con |
| Cluster1101 | 0 | 7tm_2, GPS, Pentaxin |
| Cluster1102 | 0 | IL8 |
| Cluster1121 | 1 | Ras, SOCS_box |
| Cluster1126 | 2 | Ank_2, Ank_3, Ank_4, SOCS_box |
| Cluster1132 | 1 | RRM_1 |
| Cluster1171 | 0 | Histone, Histone_H2A_C |
| Cluster1181 | 1 | DPPIV_N, Peptidase_S9 |
| Cluster1200 | 1 | Carb_anhydrase |
| Cluster1207 | 1 | Cation_ATPase, E1-E2_ATPase, PhoLip_ATPase_C, PhoLip_ATPase_N |
| Cluster1231 | 1 | CTP_transf_3 |
| Cluster1237 | 1 | Ald_Xan_dh_C, Ald_Xan_dh_C2, CO_deh_flav_C, FAD_binding_5, Fer2, Fer2_2 |
| Cluster1238 | 1 | TBP |
| Cluster1300 | 1 | EGF_CA, FXa_inhibition, Ldl_recept_a, Ldl_recept_b, cEGF |
| Cluster1312 | 1 | TspO_MBR |
| Cluster1322 | 1 | DEP, G-gamma, RGS |
| Cluster1339 | 1 | wnt |
| Cluster1353 | 1 | CH, DUF3585, NT-C2 |
| Cluster1398 | 1 | Cadherin, Cadherin_2, Protocadherin |
| Cluster1405 | 1 | C1-set, V-set |
| Cluster1436 | 1 | eIF-5a |
| Cluster1457 | 1 | Pkinase |
| Cluster1474 | 1 | Y_phosphatase, fn3 |
| Cluster1488 | 1 | DDHD |
| Cluster1492 | 1 | Homeobox |
| Cluster1503 | 1 | Death, TNFR_c6 |
| Cluster1553 | 1 | Scramblase |
| Cluster1558 | 1 | Popeye |
| Cluster1561 | 1 | - |
| Cluster1563 | 1 | K_oxygenase, Pyr_redox_2, Pyr_redox_3 |
| Cluster1570 | 1 | 7tm_1 |
| Cluster1572 | 0 | Pkinase, RGS |
| Cluster1589 | 1 | AbLIM_anchor, LIM, VHP |
| Cluster1603 | 1 | Vps55 |
| Cluster1626 | 1 | Fringe |
| Cluster1632 | 1 | ITI_HC_C, VIT, VWA |
| Cluster1643 | 1 | Macro, Pkinase |
| Cluster1646 | 1 | HECT, RCC1 |
| Cluster1649 | 1 | Homeobox |
| Cluster1650 | 1 | RINGv |
| Cluster1652 | 2 | Ank, Ank_2, Ank_3, Ank_4 |
| Cluster1656 | 1 | EF-hand_1, EF-hand_5, EF-hand_7 |
| Cluster1678 | 1 | MA3, MIF4G, W2 |
| Cluster1693 | 1 | Guanylate_kin, L27, PDZ, SH3_2 |
| Cluster1701 | 1 | C2, FYVE_2 |
| Cluster1707 | 1 | DUF4588 |
| Cluster1708 | 1 | ParA |
| Cluster1719 | 1 | Kunitz_BPTI, Ldl_recept_a, MANEC |
| Cluster1726 | 1 | MFS_1 |
| Cluster1732 | 1 | AAA, Vps4_C |
| Cluster1741 | 0 | 7tm_2, GPS, SEA |
| Cluster1779 | 0 | MFS_2 |
| Cluster1791 | 0 | Aldo_ket_red |
| Cluster1804 | 1 | Carb_anhydrase |
| Cluster1832 | 1 | ADAM_spacer1, PLAC, Pep_M12B_propep, Reprolysin, TSP_1 |
| Cluster1834 | 1 | SNF |
| Cluster1847 | 1 | FCH, SH3_1 |
| Cluster1878 | 1 | I-set, Sema, ig |
| Cluster1888 | 1 | 7tm_3, ANF_receptor, NCD3G |
| Cluster1894 | 1 | Hormone_recep, zf-C4 |
| Cluster1896 | 1 | 7tm_2, HRM |
| Cluster1902 | 1 | - |
| Cluster1926 | 1 | DAGAT |
| Cluster1930 | 1 | Annexin |
| Cluster1937 | 1 | UCH |
| Cluster1948 | 1 | PINIT, zf-MIZ |
| Cluster1962 | 1 | DDHD, SAM_1, WWE |
| Cluster1966 | 1 | Cadherin, Cadherin_2 |
| Cluster1973 | 1 | Proteasome |
| Cluster1974 | 1 | Ank, Ank_2, Ank_3, Ank_4 |
| Cluster1987 | 1 | PfkB |
| Cluster1993 | 1 | SEA |
| Cluster2005 | 1 | EGF, Laminin_EGF, Laminin_N |
| Cluster2006 | 1 | Evr1_Alr, Thioredoxin |
| Cluster2016 | 1 | AA_permease_2, AA_permease_C |
| Cluster2036 | 1 | HLH |
| Cluster2037 | 1 | Guanylate_kin, L27, PDZ, SH3_2 |
| Cluster2052 | 1 | UBA, UQ_con |
| Cluster2060 | 1 | WH2 |
| Cluster2087 | 1 | Serpin |
| Cluster2088 | 1 | Pkinase |
| Cluster2095 | 0 | AMOP, TSP_1 |
| Cluster2097 | 1 | AbLIM_anchor, LIM, VHP |
| Cluster2105 | 1 | PH, RhoGAP |
| Cluster2108 | 1 | EF-hand_1, EF-hand_5, EF-hand_6, EF-hand_7 |
| Cluster2109 | 1 | C2 |
| Cluster2124 | 1 | RRM_1 |
| Cluster2126 | 1 | Dickkopf_N, Prokineticin |
| Cluster2131 | 1 | PPDFL |
| Cluster2133 | 1 | BMP2K_C, Pkinase |
| Cluster2134 | 1 | ADAM_spacer1, PLAC, Pep_M12B_propep, Reprolysin, TSP_1 |
| Cluster2163 | 1 | CRAL_TRIO, CRAL_TRIO_N, PRELI |
| Cluster2170 | 0 | SCF |
| Cluster2172 | 1 | PWWP, SET |
| Cluster2175 | 1 | Endonuclease_NS, Phosphodiest, Somatomedin_B |
| Cluster2179 | 1 | Ets |
| Cluster2188 | 1 | Pkinase |
| Cluster2189 | 1 | Ldh_1_C, Ldh_1_N |
| Cluster2194 | 1 | Arm, Arm_3, IBB |
| Cluster2201 | 1 | Serinc |
| Cluster2204 | 1 | p450 |
| Cluster2208 | 1 | DUF1011 |
| Cluster2211 | 1 | Drf_FH3, Drf_GBD, FH2 |
| Cluster2213 | 0 | Stathmin |
| Cluster2241 | 1 | HUN, UBN_AB |
| Cluster2271 | 1 | Glyco_transf_29 |
| Cluster2274 | 1 | Sec1 |
| Cluster2286 | 1 | TAFA |
| Cluster2287 | 1 | EF-hand_1, EF-hand_5, EF-hand_7, EF-hand_8 |
| Cluster2290 | 1 | EGF_2, EGF_CA, FXa_inhibition, Laminin_EGF, cEGF |
| Cluster2291 | 1 | Beta-lactamase |
| Cluster2300 | 1 | PA, Peptidase_A22B |
| Cluster2312 | 1 | HLH, Hairy_orange |
| Cluster2317 | 1 | C2, SMP_LBD |
| Cluster2349 | 1 | Neur_chan_LBD, Neur_chan_memb |
| Cluster2356 | 0 | F-box-like, LRR_6 |
| Cluster2392 | 1 | Plectin |
| Cluster2395 | 1 | T-box, T-box_assoc |
| Cluster2398 | 0 | TNFR_c6 |
| Cluster2449 | 1 | Yip1 |
| Cluster2451 | 1 | Aminotran_4 |
| Cluster2454 | 1 | PTB, SH3_1 |
| Cluster2462 | 1 | DUF4211 |
| Cluster2463 | 1 | Adaptin_N, Alpha_adaptinC2, Alpha_adaptin_C |
| Cluster2464 | 1 | Ion_trans, PAS_9, cNMP_binding |
| Cluster2468 | 1 | C2-set_2, Ig_2, Ig_3, V-set, ig |
| Cluster2480 | 1 | PH |
| Cluster2484 | 1 | CAP |
| Cluster2512 | 1 | Pkinase |
| Cluster2525 | 1 | Ras |
| Cluster2534 | 1 | WH1 |
| Cluster2545 | 1 | Ribosomal_S17e |
| Cluster2546 | 1 | SPATA6 |
| Cluster2559 | 1 | PAP2 |
| Cluster2562 | 1 | Ldl_recept_a, MACPF, Sushi, TSP_1 |
| Cluster2582 | 1 | Lipocalin |
| Cluster2587 | 1 | Got1 |
| Cluster2594 | 1 | CAP |
| Cluster2603 | 1 | SCAMP |
| Cluster2631 | 1 | Stathmin |
| Cluster2632 | 1 | Endothelin |
| Cluster2651 | 1 | Sprouty, WH1 |
| Cluster2673 | 1 | Peptidase_C54 |
| Cluster2688 | 1 | zf-C2H2 |
| Cluster2693 | 1 | HDAC4_Gln, Hist_deacetyl |
| Cluster2702 | 1 | L_HMGIC_fpl |
| Cluster2711 | 1 | zf-C2H2 |
| Cluster2713 | 1 | Iso_dh |
| Cluster2718 | 1 | Abhydrolase_1 |
| Cluster2722 | 1 | IF4E |
| Cluster2725 | 1 | PSI |
| Cluster2727 | 1 | C2-set_2, I-set, Ig_2, Ig_3, ig |
| Cluster2736 | 1 | RHD_DNA_bind, RHD_dimer |
| Cluster2743 | 1 | C2, EF-hand_10, EF-hand_like, PH, PI-PLC-X, PI-PLC-Y |
| Cluster2754 | 1 | LRRNT, LRR_8 |
| Cluster2766 | 1 | Lysyl_oxidase, SRCR |
| Cluster2769 | 1 | Transglut_core |
| Cluster2781 | 1 | Consortin_C |
| Cluster2799 | 1 | RRM_1, RRM_5 |
| Cluster2801 | 1 | GDPD |
| Cluster2803 | 1 | Hemopexin, Somatomedin_B |
| Cluster2805 | 0 | Cofilin_ADF |
| Cluster2811 | 1 | Abi_HHR |
| Cluster2816 | 0 | EF-hand_1, EF-hand_7, EF-hand_8 |
| Cluster2820 | 1 | I-set, Ig_3, fn3 |
| Cluster2822 | 1 | 7tm_1 |
| Cluster2828 | 0 | ATP-synt_ab, ATP-synt_ab_N, ATP-synt_ab_Xtn |
| Cluster2832 | 1 | CAP, LCCL |
| Cluster2834 | 1 | LIM |
| Cluster2836 | 1 | Lipase_GDSL |
| Cluster2847 | 0 | Arf |
| Cluster2860 | 0 | START |
| Cluster2869 | 1 | ADP_ribosyl_GH |
| Cluster2879 | 1 | TMEM189_B_dmain |
| Cluster2886 | 0 | EF-hand_1, EF-hand_5, EF-hand_7, EF-hand_8 |
| Cluster2908 | 1 | DUF3534, Lyase_aromatic |
| Cluster2910 | 1 | Beta-TrCP_D, F-box-like, WD40 |
| Cluster2915 | 1 | Disintegrin, Pep_M12B_propep, Reprolysin_2, Reprolysin_5 |
| Cluster2924 | 1 | PDGF, VEGF_C |
| Cluster2928 | 0 | Y_phosphatase |
| Cluster2929 | 1 | PIP5K, UIM, VWA_2 |
| Cluster2934 | 1 | EamA |
| Cluster2937 | 1 | Pkinase_Tyr |
| Cluster2951 | 1 | COLFI, Collagen, VWC |
| Cluster2969 | 1 | Patatin, cNMP_binding |
| Cluster2977 | 1 | G6PD_C, G6PD_N |
| Cluster2978 | 1 | PP2C |
| Cluster2982 | 1 | Interfer-bind, fn3 |
| Cluster2986 | 1 | EXS, SPX |
| Cluster2988 | 1 | CARMIL_C, LRR_6 |
| Cluster3000 | 1 | RRM_1, zf-CCHC |
| Cluster3001 | 1 | DUF4517 |
| Cluster3005 | 1 | VASP_tetra, WH1 |
| Cluster3026 | 1 | A_deaminase |
| Cluster3040 | 1 | Cadherin |
| Cluster3051 | 1 | Activin_recp, Pkinase, Pkinase_Tyr |
| Cluster3052 | 1 | AlaDh_PNT_C, AlaDh_PNT_N, PNTB, PNTB_4TM |
| Cluster3061 | 1 | T-box |
| Cluster3065 | 1 | dCMP_cyt_deam_1 |
| Cluster3068 | 0 | CUB, Zona_pellucida |
| Cluster3081 | 1 | DUF3377, Hemopexin, PG_binding_1, Peptidase_M10 |
| Cluster3084 | 0 | PH, PLDc, PLDc_2, PX |
| Cluster3095 | 0 | ANF_receptor, Lig_chan, Lig_chan-Glu_bd |
| Cluster3111 | 0 | Lys |
| Cluster3125 | 1 | EGF_CA, FXa_inhibition, Matrilin_ccoil, VWA |
| Cluster3126 | 1 | HIF-1, PAS, PAS_3 |
| Cluster3132 | 1 | Fascin |
| Cluster3148 | 1 | MFS_1 |
| Cluster3174 | 1 | ELM2, Myb_DNA-binding |
| Cluster3185 | 1 | DHR-2, DOCK-C2, DOCK_N, SH3_2 |
| Cluster3188 | 1 | C8, Cys_knot, Mucin2_WxxW, VWD |
| Cluster3212 | 1 | IRF-2BP1_2, zf-C3HC4 |
| Cluster3229 | 1 | adh_short |
| Cluster3282 | 1 | CP2 |
| Cluster3320 | 0 | Cadherin, Cadherin_2, Cadherin_C_2 |
| Cluster3324 | 1 | adh_short, adh_short_C2 |
| Cluster3328 | 1 | Bax1-I |
| Cluster3335 | 1 | DBINO, HAND, Helicase_C, SLIDE, SNF2_N |
| Cluster3343 | 0 | Peptidase_C97 |
| Cluster3345 | 1 | DUF1241 |
| Cluster3387 | 1 | MATH, zf-C3HC4, zf-C3HC4_2, zf-TRAF |
| Cluster3388 | 1 | SpoU_methylase, SpoU_sub_bind |
| Cluster3397 | 0 | Neur_chan_LBD, Neur_chan_memb |
| Cluster3404 | 1 | 3HCDH, 3HCDH_N, ECH_1 |
| Cluster3416 | 1 | Proteasome, Proteasome_A_N |
| Cluster3426 | 1 | C1_1, SH3_1, SH3_9, STAC2_u1 |
| Cluster3453 | 1 | Pkinase |
| Cluster3458 | 1 | IMD, WH2 |
| Cluster3460 | 0 | 7tm_1 |
| Cluster3470 | 1 | AAA |
| Cluster3482 | 1 | Tsg |
| Cluster3516 | 1 | EGF_2, I-set, Ig_Tie2_1, Pkinase_Tyr, fn3 |
| Cluster3538 | 1 | Guanylate_kin, MAGUK_N_PEST, PDZ, PDZ_assoc, SH3_1, SH3_2 |
| Cluster3544 | 0 | Zona_pellucida |
| Cluster3590 | 0 | I-set, V-set |
| Cluster3596 | 0 | ADAM_CR, Disintegrin, Pep_M12B_propep, Reprolysin |
| Cluster3608 | 1 | DUF1899, Pam16, WD40, WD40_4 |
| Cluster3622 | 2 | 7tm_2, GPS |
| Cluster3646 | 0 | Ribosomal_L19e |
| Cluster3658 | 1 | CRIC_ras_sig, DUF1170, PDZ, PH, SAM_1 |
| Cluster3661 | 0 | 7tm_1 |
| Cluster3665 | 1 | 7tm_1, LRR_8, Ldl_recept_a |
| Cluster3675 | 1 | C1_1, C2, Pkinase, Pkinase_C |
| Cluster3679 | 0 | PCI |
| Cluster3681 | 1 | EGF_3, EGF_CA, TSP_1, TSP_3, TSP_C, VWC |
| Cluster3684 | 0 | FAM196 |
| Cluster3708 | 1 | Cadherin |
| Cluster3734 | 1 | zf-C2H2, zf-C2H2_11, zf-C2H2_assoc2 |
| Cluster3748 | 1 | COMP, EGF_CA, TSP_3, TSP_C |
| Cluster3765 | 0 | CBS |
| Cluster3768 | 1 | ADAM_CR, Disintegrin, Pep_M12B_propep, Reprolysin |
| Cluster3788 | 1 | Hormone_recep, Nuc_recep-AF1, zf-C4 |
| Cluster3800 | 1 | Homeobox, Hox9_act |
| Cluster3818 | 1 | Ank_4 |
| Cluster3823 | 0 | NRN1 |
| Cluster3857 | 0 | Arrestin_C, Arrestin_N |
| Cluster3860 | 1 | Methyltransf_3 |
| Cluster3875 | 0 | ABC2_membrane_3, ABC_tran |
| Cluster3969 | 1 | CDP-OH_P_transf |
| Cluster3997 | 1 | Cys_knot, IGFBP, TSP_1, VWC |
| Cluster3998 | 0 | 7tm_1 |
| Cluster4002 | 0 | Troponin |
| Cluster4029 | 1 | Pacs-1 |
| Cluster4032 | 1 | Pkinase, Pkinase_Tyr |
| Cluster4064 | 1 | 7tm_2, HRM |
| Cluster4078 | 1 | AA_permease_2 |
| Cluster4091 | 0 | CTC1 |
| Cluster4118 | 0 | PAN_1, PAN_4, Trypsin |
| Cluster4139 | 0 | zf-C3HC4_3 |
| Cluster4145 | 1 | CaM-KIIN |
| Cluster4175 | 1 | C1_1, PH, RII_binding_1, RhoGEF |
| Cluster4199 | 0 | Choline_transpo |
| Cluster4252 | 0 | FOLN, Kazal_2 |
| Cluster4258 | 0 | Prothymosin |
| Cluster4281 | 0 | Sushi |
| Cluster4300 | 0 | GFO_IDH_MocA, GFO_IDH_MocA_C |
| Cluster4309 | 0 | Activin_recp, Pkinase, Pkinase_Tyr, TGF_beta_GS |
| Cluster4352 | 0 | BRCT, BRCT_2, PARP, VIT, VWA, VWA_3 |
| Cluster4364 | 0 | C1_1, CAMSAP_CH, CH, PH, PH_10, RhoGEF, SH2, SH3_1, SH3_2, SH3_9 |
| Cluster4432 | 0 | Aminotran_1_2 |
| Cluster4449 | 1 | HSBP1 |
| Cluster4455 | 1 | CHGN |
| Cluster4456 | 1 | CCDC74_C, CCDC92 |
| Cluster4465 | 0 | YAF2_RYBP, zf-RanBP |
| Cluster4500 | 0 | RRM_1 |
| Cluster4513 | 0 | Arf |
| Cluster4532 | 0 | AZUL, HECT |
| Cluster4571 | 1 | Pkinase |
| Cluster4591 | 0 | Cohesin_HEAT, Nipped-B_C |
| Cluster4607 | 0 | HLH, Hairy_orange |
| Cluster4653 | 0 | Ets |
| Cluster4657 | 0 | Amidase_2 |
| Cluster4697 | 0 | C2-set_2, V-set |
| Cluster4699 | 0 | V-SNARE_C |
| Cluster4716 | 0 | Ephrin |
| Cluster4735 | 0 | DDA1 |
| Cluster4820 | 0 | I-set, LRR_8, fn3 |
| Cluster4839 | 0 | IF4E |
| Cluster4840 | 0 | TSC22 |
| Cluster4841 | 1 | ADK |
| Cluster4887 | 0 | EphA2_TM, Ephrin_lbd, Ephrin_rec_like, Pkinase_Tyr, SAM_1, fn3 |
| Cluster4892 | 0 | Shisa |
| Cluster5001 | 0 | AA_permease, SLC12 |
| Cluster5094 | 0 | Hormone_recep, zf-C4 |
| Cluster5097 | 0 | DUF1899, WD40, WD40_4 |
| Cluster5107 | 0 | Alpha_L_fucos, Fucosidase_C |
| Cluster5126 | 0 | Lactamase_B_3, Rieske |
| Cluster5219 | 0 | BCL_N |
| Cluster5275 | 1 | C2-set_2, V-set |
| Cluster5285 | 0 | LTD |
| Cluster5320 | 0 | - |
| Cluster5329 | 0 | IRK |
| Cluster5443 | 0 | PH, Pkinase, Pkinase_C |
| Cluster5470 | 0 | EGF, VWA, hEGF |
| Cluster5514 | 0 | FERM_M, IQ, MyTH4, Myosin_head, SH3_2 |
| Cluster5561 | 0 | TB2_DP1_HVA22 |
| Cluster5576 | 0 | DUF758 |
| Cluster5613 | 0 | P34-Arc |
| Cluster5615 | 0 | PRELI |
| Cluster5749 | 0 | Frag1 |
| Cluster5765 | 0 | Fib_alpha, Fibrinogen_C |
| Cluster5770 | 0 | IPK |
| Cluster5809 | 0 | Atg8 |
| Cluster5831 | 0 | I-set, Ig_3, LRR_8 |
| Cluster5857 | 0 | Strabismus |
| Cluster5861 | 0 | DUF3808, TPR_6, TPR_8 |
| Cluster5906 | 0 | MMR_HSR1, YchF-GTPase_C |
| Cluster5992 | 1 | AAA_16, Guanylate_cyc |
| Cluster5999 | 0 | Laminin_B, Laminin_EGF, Laminin_G_1, Laminin_G_2, Laminin_I, Laminin_II, Laminin_N |
| Cluster6062 | 0 | WD40 |
| Cluster6143 | 0 | - |
| Cluster6170 | 0 | Ras |
| Cluster6186 | 0 | Josephin |
| Cluster6195 | 0 | PRY, SPRY |
| Cluster6237 | 0 | ABC_membrane, ABC_tran |
| Cluster6243 | 0 | DUF1180 |
| Cluster6294 | 0 | Laminin_G_1 |
| Cluster6309 | 0 | - |
| Cluster6501 | 0 | RRM_1 |
| Cluster6510 | 0 | PID, SH2 |
| Cluster6669 | 0 | NAC |
| Cluster6677 | 0 | Fz, Ldl_recept_a, SRCR_2, Trypsin |
| Cluster6692 | 0 | V-set |
| Cluster6728 | 0 | 7tm_1 |
| Cluster6887 | 0 | HIT |
| Cluster6925 | 0 | Armet |
| Cluster7181 | 0 | Kazal_2, Ldl_recept_a, SRCR, Trypsin |
| Cluster7213 | 0 | Kringle, PAN_1, Trypsin |
| Cluster7226 | 0 | USP8_interact, zf-C3HC4_2 |
| Cluster7399 | 0 | Peptidase_S28 |
| Cluster7416 | 0 | ATP-synt_G |
| Cluster7493 | 0 | Iwr1 |
| Cluster7494 | 0 | Ank_2, DUF3454, EGF, EGF_CA, NOD, NODP, Notch, hEGF |
| Cluster7495 | 0 | ArfGap, PH |
| Cluster7586 | 0 | LUC7 |
| Cluster7595 | 0 | MOZ_SAS, Tudor-knot |
| Cluster7622 | 0 | F5_F8_type_C, Pkinase_Tyr |
| Cluster7825 | 0 | PX |
| Cluster7859 | 0 | Aida_C2, Aida_N |
| Cluster7947 | 0 | EGF_CA, FXa_inhibition, Ldl_recept_a, Ldl_recept_b, cEGF |
| Cluster7950 | 0 | START |
| Cluster7956 | 0 | Cystatin |
| Cluster7970 | 0 | Ribosomal_L37 |
| Cluster7988 | 0 | Oxidored_q6 |
| Cluster8101 | 0 | Dpy-30 |
| Cluster8186 | 0 | DNA_pol_A_exo1, HRDC, PMC2NT |
| Cluster8199 | 0 | Serpin, V-set |
| Cluster8263 | 0 | Proteasome, Proteasome_A_N |
| Cluster8295 | 0 | MFS_1, Sugar_tr |
| Cluster8299 | 0 | CD34_antigen |
| Cluster8343 | 0 | WW |
| Cluster8355 | 0 | RCC1, RCC1_2 |
| Cluster8520 | 0 | C1-set, V-set, ig |
| Cluster8544 | 0 | DUF4604 |
| Cluster8715 | 0 | Aminotran_1_2 |
| Cluster8766 | 0 | - |
| Cluster8806 | 0 | BIR, CARD, zf-C3HC4_3 |
| Cluster8813 | 0 | Cyclin_C, Cyclin_N |
| Cluster8854 | 0 | Neur_chan_LBD, Neur_chan_memb |
| Cluster8867 | 0 | Cyclin_N |
| Cluster8972 | 0 | - |
| Cluster8975 | 0 | TFIID-18kDa |
| Cluster9170 | 0 | - |
| Cluster9233 | 0 | 4F5 |
| Cluster9376 | 0 | DAO |
| Cluster9403 | 0 | Annexin |
| Cluster9483 | 0 | Bin3 |
| Cluster9488 | 0 | Ribosom_S12_S23 |
| Cluster9560 | 0 | - |
| Cluster9621 | 0 | CBFD_NFYB_HMF |
| Cluster9634 | 0 | Lipocalin |
| Cluster9750 | 0 | Cystatin, Inhibitor_I29, Peptidase_C1 |
| Cluster9759 | 0 | Cor1 |
| Cluster9807 | 0 | Vps26 |
| Cluster9853 | 0 | - |
| Cluster9905 | 0 | adh_short |
| Cluster9986 | 0 | BAR, RhoGEF, SH3_2, SH3_9 |
| Cluster10001 | 0 | ADAM_spacer1, Pep_M12B_propep, Reprolysin, TSP_1 |
| Cluster10062 | 0 | Lamp |
| Cluster10118 | 0 | Ank_2, Ank_4, Ank_5 |
| Cluster10156 | 0 | Guanylate_cyc_2 |
| Cluster10176 | 0 | RRM_1, tRNA_U5-meth_tr |
| Cluster10179 | 0 | CUB, Kringle, WSC |
| Cluster10190 | 0 | FHA, KIF1B, Kinesin |
| Cluster10195 | 0 | MORN |
| Cluster10267 | 0 | UPF0086 |
| Cluster10378 | 0 | - |
| Cluster10420 | 0 | F-box, F-box-like, SPRY |
| Cluster10459 | 0 | BTB, Kelch_1, Kelch_3, Kelch_4 |
| Cluster10475 | 0 | - |
| Cluster10518 | 0 | FCH, Pkinase_Tyr, SH2 |
| Cluster10572 | 0 | DSPc |
| Cluster10689 | 0 | RAD51_interact |
| Cluster10690 | 0 | WD40 |
| Cluster10765 | 0 | DUF2367 |
| Cluster10783 | 0 | PIH1 |
| Cluster10789 | 0 | DEAD_2, Helicase_C_2 |
| Cluster10809 | 0 | Cu-oxidase, Cu-oxidase_2, Cu-oxidase_3, F5_F8_type_C |
| Cluster10834 | 0 | ITAM, Ig_3, V-set, ig |
| Cluster10872 | 0 | GPS, PKD, PKD_channel, PLAT, REJ, WSC |
| Cluster10884 | 0 | TPD52 |
| Cluster10898 | 0 | TRM |
| Cluster10904 | 0 | zf-C2H2 |
| Cluster11008 | 0 | adh_short, adh_short_C2 |
| Cluster11080 | 0 | zf-Tim10_DDP |
| Cluster11081 | 0 | Use1 |
| Cluster11094 | 0 | DUF1741 |
| Cluster11099 | 0 | Cyt-b5 |
| Cluster11154 | 0 | WD40 |
| Cluster11189 | 0 | hSac2 |
| Cluster11191 | 0 | tRNA-synt_1c, tRNA-synt_1c_C, tRNA_synt_1c_R1, tRNA_synt_1c_R2 |
| Cluster11209 | 0 | C1_1, Pkinase_Tyr, RBD |
| Cluster11250 | 0 | PTN_MK_C, PTN_MK_N |
| Cluster11257 | 0 | I-set, fn3 |
| Cluster11264 | 0 | ACT, Biopterin_H |
| Cluster11304 | 0 | - |
| Cluster11315 | 0 | EF-hand_1, EF-hand_5, EF-hand_6, EF-hand_7, FAD_binding_8, Ferric_reduct, NAD_binding_6 |
| Cluster11318 | 0 | - |
| Cluster11376 | 0 | Homeobox |
| Cluster11410 | 0 | ALG3 |
| Cluster11411 | 0 | Ribonuc_P_40 |
| Cluster11414 | 0 | DFRP_C, RWD |
| Cluster11415 | 0 | Peptidase_C78, zf-C2H2 |
| Cluster11423 | 0 | DSPc |
| Cluster11445 | 0 | Tropomodulin |
| Cluster11451 | 0 | DC_STAMP |
| Cluster11457 | 0 | CS, Cyt-b5, FAD_binding_6, NAD_binding_1 |
| Cluster11490 | 0 | PDZ, PH |
| Cluster11495 | 0 | APG12 |
| Cluster11522 | 0 | F-box-like |
| Cluster11554 | 0 | Pkinase |
| Cluster11569 | 0 | DUF775 |
| Cluster11575 | 0 | Ldh_1_C, Ldh_1_N |
| Cluster11648 | 0 | Homeobox_KN |
| Cluster11659 | 0 | TatD_DNase |
| Cluster11671 | 0 | EpoR_lig-bind, fn3 |
| Cluster11687 | 0 | FGE-sulfatase |
| Cluster11699 | 0 | WD40 |
| Cluster11709 | 0 | Pkinase, RGS |
| Cluster11743 | 0 | WD40 |
| Cluster11748 | 0 | Aminotran_3 |
| Cluster11777 | 0 | STAS, Sulfate_transp |
| Cluster11778 | 0 | BIR |
| Cluster11807 | 0 | CCM2_C |
| Cluster11817 | 0 | Glycos_transf_2 |
| Cluster11883 | 0 | B56 |
| Cluster11944 | 0 | ANTH |
| Cluster11965 | 0 | - |
| Cluster11968 | 0 | 7tm_1 |
| Cluster11972 | 0 | Sema |
| Cluster12005 | 0 | Tropomodulin |
| Cluster12044 | 0 | Lipase |
| Cluster12075 | 0 | CFC |
| Cluster12083 | 0 | Cadherin, Cadherin_2, Cadherin_C_2, Cadherin_tail |
| Cluster12106 | 0 | BAR, PX, Vps5 |
| Cluster12115 | 0 | Med11 |
| Cluster12149 | 0 | Guanylate_cyc, HNOB, HNOBA |
| Cluster12152 | 0 | RhoGEF |
| Cluster12192 | 0 | DUF3402, N1221 |
| Cluster12199 | 0 | F_actin_cap_B |
| Cluster12222 | 0 | TPR_1, TPR_16, TPR_2, TPR_8 |
| Cluster12267 | 0 | Ribosomal_L5, Ribosomal_L5_C |
| Cluster12281 | 0 | Evr1_Alr |
| Cluster12304 | 0 | Prp19, U-box, WD40 |
| Cluster12320 | 0 | RNA_pol_L_2 |
| Cluster12344 | 0 | PTS_2-RNA |
| Cluster12367 | 0 | Nexin_C, PXA |
| Cluster12434 | 0 | I-set, Ig_2, Ig_3, Pkinase_Tyr, fn3, ig |
| Cluster12452 | 0 | Gal-bind_lectin |
| Cluster12453 | 0 | DEP, PH |
| Cluster12454 | 0 | EGF_CA |
| Cluster12457 | 0 | DUF4061 |
| Cluster12464 | 0 | Runt, RunxI |
| Cluster12511 | 0 | DED |
| Cluster12512 | 0 | Ig_3, V-set |
| Cluster12517 | 0 | RAMP |
| Cluster12519 | 0 | SSF |
| Cluster12524 | 0 | zf-DBF |
| Cluster12526 | 0 | Trypsin |
| Cluster12533 | 0 | JMY, WH2, WHAMM-JMY_N |
| Cluster12545 | 0 | Creatinase_N, Creatinase_N_2, Peptidase_M24, Peptidase_M24_C |
| Cluster12555 | 0 | Cmc1 |
| Cluster12565 | 0 | Ets, SAM_PNT |
| Cluster12577 | 0 | adh_short |
| Cluster12603 | 0 | LRR_8, VWC |
| Cluster12612 | 0 | Collagen |
| Cluster12622 | 0 | Peptidase_M17 |
| Cluster12626 | 0 | ApoO |
| Cluster12634 | 0 | C2 |
| Cluster12639 | 0 | Pkinase, cNMP_binding |
| Cluster12641 | 0 | - |
| Cluster12666 | 0 | dsrm |
| Cluster12691 | 0 | ABC2_membrane_3, ABC_tran |
| Cluster12704 | 0 | NAD_binding_1 |
| Cluster12709 | 0 | PB1, PX, SH3_1, SH3_2, SH3_9 |
| Cluster12787 | 0 | SNF |
| Cluster12788 | 0 | Ank_2, Ank_4, GPCR_chapero_1, UIM |
| Cluster12800 | 0 | Collagen, VWA |
| Cluster12801 | 0 | GDI |
| Cluster12823 | 0 | MFS_1, Sugar_tr |
| Cluster12832 | 0 | Mic1 |
| Cluster12839 | 0 | Tmemb_18A |
| Cluster12849 | 0 | Mnd1 |
| Cluster12852 | 0 | EZH2_WD-Binding, SET |
| Cluster12858 | 0 | AAA, DNA_pol3_delta2, Rep_fac_C |
| Cluster12869 | 0 | Kringle |
| Cluster12871 | 0 | adh_short, adh_short_C2 |
| Cluster12877 | 0 | HIT |
| Cluster12892 | 0 | Ras |
| Cluster12893 | 0 | BRK, Chromo, Helicase_C, SNF2_N |
| Cluster12894 | 0 | CRAL_TRIO, CRAL_TRIO_N |
| Cluster12898 | 0 | T_cell_tran_alt |
| Cluster12918 | 0 | PAC4 |
| Cluster12951 | 0 | BetaGal_dom4_5, Glyco_hydro_35 |
| Cluster12961 | 0 | LSM |
| Cluster13077 | 0 | TRAM1, TRAM_LAG1_CLN8 |
| Cluster13079 | 0 | DHHC |
| Cluster13080 | 0 | AP3D1, Adaptin_N |
| Cluster13118 | 0 | DUF108, NAD_binding_3 |
| Cluster13154 | 0 | FA, FERM_C, FERM_M, FERM_N, PDZ, Y_phosphatase |
| Cluster13175 | 0 | - |
| Cluster13201 | 0 | DUF1232, zf-C3HC4, zf-C3HC4_3, zf-RING_UBOX |
| Cluster13250 | 0 | IL8 |
| Cluster13281 | 0 | - |
| Cluster13287 | 0 | bZIP_1 |
| Cluster13314 | 0 | IL17R_fnIII_D1, IL17R_fnIII_D2, SEFIR |
| Cluster13319 | 0 | - |
| Cluster13320 | 0 | Calx-beta, EGF_2, Integrin_B_tail, Integrin_beta, PSI_integrin, fn3 |
| Cluster13328 | 0 | MRP-S33 |
| Cluster13339 | 0 | WWE |
| Cluster13343 | 0 | I-set, Sema |
| Cluster13394 | 0 | Dynamin_N, EF-hand_4, EHD_N |
| Cluster13395 | 0 | LRR_1, LRR_4, LRR_6, LRR_8 |
| Cluster13400 | 0 | LRR_4 |
| Cluster13402 | 0 | Ephrin_rec_like |
| Cluster13427 | 0 | Peptidase_M14, Propep_M14, ShK |
| Cluster13444 | 0 | DUF4460, DUF4461 |
| Cluster13451 | 0 | DCX |
| Cluster13469 | 0 | - |
| Cluster13473 | 0 | Ephrin |
| Cluster13479 | 0 | - |
| Cluster13505 | 0 | LURAP, zf-ANAPC11 |
| Cluster13565 | 0 | FKBP_C |
| Cluster13610 | 0 | LRR_4, LRR_6, LRR_8, LRR_9 |
| Cluster13611 | 0 | - |
| Cluster13615 | 0 | 7tm_3, ANF_receptor, NCD3G |
| Cluster13624 | 0 | FYTT |
| Cluster13639 | 0 | Aminotran_1_2 |
| Cluster13660 | 0 | MAJIN |
| Cluster13711 | 0 | BTK, C2, PH, RasGAP |
| Cluster13728 | 0 | IQ |
| Cluster13753 | 0 | Homeobox |
| Cluster13775 | 0 | zf-C2H2 |
| Cluster13779 | 0 | PH |
| Cluster13787 | 0 | DUF842 |
| Cluster13805 | 0 | MH1, MH2 |
| Cluster13815 | 0 | LURAP |
| Cluster13876 | 0 | DUF2464 |
| Cluster13898 | 0 | PAC3 |
| Cluster13922 | 0 | S1, SHS2_Rpb7-N |
| Cluster13924 | 0 | PLDc_2, PLDc_3 |
| Cluster13937 | 0 | Glyco_tran_28_C |
| Cluster13938 | 0 | CH, PH, RhoGEF, RhoGEF67_u1, RhoGEF67_u2, SH3_1, SH3_2, SH3_9, betaPIX_CC |
| Cluster13950 | 0 | Mab-21 |
| Cluster13969 | 0 | Fibrinogen_C |
| Cluster13976 | 0 | p25-alpha |
| Cluster13979 | 0 | CAP_GLY, DUF3694, FHA, KIF1B, Kinesin, Kinesin_assoc |
| Cluster13984 | 0 | SNF |
| Cluster13991 | 0 | NDUF_B6 |
| Cluster14021 | 0 | Nop25 |
| Cluster14076 | 0 | Homeobox |
| Cluster14083 | 0 | - |
| Cluster14094 | 0 | DUF498 |
| Cluster14124 | 0 | 7tm_1 |
| Cluster14150 | 0 | TMEM220 |
| Cluster14158 | 0 | A2M, A2M_N, A2M_N_2, A2M_comp, A2M_recep, ANATO, NTR, Thiol-ester_cl |
| Cluster14175 | 0 | BSMAP |
| Cluster14186 | 0 | 3Beta_HSD |
| Cluster14203 | 0 | CDP-OH_P_transf |
| Cluster14296 | 0 | DUF4550 |
| Cluster14298 | 0 | FKBP_C |
| Cluster14299 | 0 | Il2rg |
| Cluster14300 | 0 | CUB, Sushi |
| Cluster14301 | 0 | Pkinase |
| Cluster14302 | 0 | ADAM_spacer1, Pep_M12B_propep, Reprolysin, TSP_1 |
| Cluster14348 | 0 | BTB_2, Ion_trans |
| Cluster14361 | 0 | HCR |
| Cluster14362 | 0 | Laminin_EGF, Laminin_G_2, Laminin_I, Laminin_II |
| Cluster14369 | 0 | SAM_1, SAM_2 |
| Cluster14400 | 0 | - |
| Cluster14430 | 0 | - |
| Cluster14431 | 0 | Ribosomal_L6e, Ribosomal_L6e_N |
| Cluster14434 | 0 | PH |
| Cluster14440 | 0 | Insulin |
| Cluster14454 | 0 | BACK, BTB, Kelch_1 |
| Cluster14498 | 0 | Synaphin |
| Cluster14575 | 0 | BTK, PH, Pkinase_Tyr, SH2, SH3_1 |
| Cluster14630 | 0 | 7tm_1 |
| Cluster14745 | 0 | MFS_1 |
| Cluster14774 | 0 | I-set, Ig_2, Ig_3, ig |
| Cluster14905 | 0 | - |
| Cluster14906 | 0 | TMEM206 |
| Cluster14907 | 0 | Kelch_4 |
| Cluster14908 | 0 | Kazal_2, SPARC_Ca_bdg, Thyroglob_assoc, Thyroglobulin_1 |
| Cluster14938 | 0 | Pkinase |
| Cluster14967 | 0 | - |
| Cluster15021 | 0 | DUF3149, EGF |
| Cluster15024 | 0 | An_peroxidase, EGF_CA, FXa_inhibition, Sushi |
| Cluster15033 | 0 | - |
| Cluster15070 | 0 | - |
| Cluster15096 | 0 | Caveolin |
| Cluster15122 | 0 | ISK_Channel |
| Cluster15179 | 0 | TSNAXIP1_N |
| Cluster15190 | 0 | C2 |
| Cluster15224 | 0 | FAM47 |
| Cluster15231 | 0 | CD225 |
| Cluster15325 | 0 | DP, E2F_TDP |
| Cluster15336 | 0 | Ribosomal_S14 |
| Cluster15397 | 0 | ig |
| Cluster15428 | 0 | Kinesin |
| Cluster15461 | 0 | - |
| Cluster15492 | 0 | Ank, Ank_2, Ank_4, Ank_5 |
| Cluster15519 | 0 | TGF_beta |
| Cluster15520 | 0 | - |
| Cluster15712 | 0 | XTBD |
| Cluster15718 | 0 | KIP1, Myosin_tail_1 |
| Cluster15883 | 0 | C1_1, PI3K_P85_iSH2, RhoGAP, SAM_2, SH2 |
| Cluster15884 | 0 | C1_1, C2, DUF1041, Membr_traf_MHD |
| Cluster15885 | 0 | F420_oxidored |
| Cluster15947 | 0 | Homeobox |
| Cluster16052 | 0 | COE1_DBD |
| Cluster16150 | 0 | 7tm_3, ANF_receptor, NCD3G |
| Cluster16154 | 0 | Guanylate_kin, MAGUK_N_PEST, PDZ, PDZ_assoc, SH3_1, SH3_2 |
| Cluster16241 | 0 | ELM2, zf-C2H2 |
| Cluster16250 | 0 | - |
| Cluster16334 | 0 | Ank_2, Ank_4 |
| Cluster16374 | 0 | Collagen, Kunitz_BPTI, VWA |
| Cluster16382 | 0 | - |
| Cluster16444 | 0 | EGF, Laminin_G_2, Syndecan |
| Cluster16601 | 0 | PDEase_I, PDEase_I_N |
| Cluster16893 | 0 | EphA2_TM, Ephrin_lbd, Ephrin_rec_like, Pkinase_Tyr, SAM_1, SAM_2, fn3 |
| Cluster16909 | 0 | LRRNT, LRR_8 |
| Cluster16998 | 0 | Calc_CGRP_IAPP |
| Cluster17004 | 0 | - |
| Cluster17006 | 0 | Trypsin |
| Cluster17183 | 0 | Collagen, Kunitz_BPTI, VWA, fn3 |
| Cluster17321 | 0 | IQ_SEC7_PH, Sec7 |
| Cluster17328 | 0 | UQ_con |
| Cluster17330 | 0 | EGF, hEGF |
| Cluster17332 | 0 | zf-C3HC4, zf-C3HC4_2, zf-C3HC4_3 |
| Cluster17334 | 0 | - |
| Cluster17349 | 0 | zf-met |
| Cluster17794 | 0 | PSI, Plexin_cytopl, Sema, TIG |
| Cluster17796 | 0 | BTB_2, Ion_trans |
| Cluster17810 | 0 | NHL, zf-B_box, zf-C3HC4, zf-RING_UBOX |
| Cluster17815 | 0 | CLCA, VWA, VWA_2 |
| Cluster18419 | 0 | EF-hand_2, EF-hand_3, ZZ |
| Cluster18423 | 0 | LLGL, Lgl_C, Synaptobrevin, WD40 |
| Cluster18427 | 0 | - |
| Cluster18434 | 0 | - |
| Cluster18438 | 0 | - |
| Cluster18456 | 0 | - |
| Cluster18460 | 0 | - |
| Cluster18469 | 0 | - |
| Cluster18471 | 0 | - |
| Cluster18475 | 0 | - |
| Cluster19837 | 0 | - |
| Cluster19849 | 0 | - |
| Cluster19875 | 0 | - |
| Cluster19902 | 0 | - |
| Cluster19928 | 0 | - |
| Cluster19940 | 0 | - |
| Cluster19942 | 0 | - |
| Cluster19943 | 0 | - |
| Cluster19948 | 0 | - |
| Cluster19957 | 0 | - |
| Cluster19960 | 0 | - |
| Cluster19963 | 0 | - |
| Cluster19974 | 0 | - |
| Cluster19980 | 0 | - |
| Cluster19993 | 0 | - |
| Cluster20002 | 0 | - |
| Cluster20008 | 0 | - |
| Cluster20020 | 0 | - |

Supplementary Table S10 Genes annotation in the sex-linked QTL region of *Clarias fuscus*.

| Gene ID | NR Annotation |
| --- | --- |
| gene-Cfus03903 | ankyrin-3-like |
| gene-Cfus03904 | ankyrin-3-like |
| gene-Cfus03905 | uncharacterized protein LOC109067360 |
| gene-Cfus03906 | unnamed protein product |
| gene-Cfus03907 | alpha-(1,6)-fucosyltransferase-like, partial |
| gene-Cfus03908 | gephyrin isoform X9 |
| gene-Cfus03909 | MAGUK p55 subfamily member 5 |
| gene-Cfus03910 | intersectin-2-like isoform X1 |
| gene-Cfus03911 | general transcription factor II-I repeat domain-containing protein 2-like |
| gene-Cfus03912 | phosphatidate phosphatase LPIN1 isoform X1 |
| gene-Cfus03913 | protein GREB1 |
| gene-Cfus03914 | -- |
| gene-Cfus03915 | -- |
| gene-Cfus03916 | -- |
| gene-Cfus03917 | spermatogenesis-associated protein 17 isoform X1 |
| gene-Cfus03918 | -- |
| gene-Cfus03919 | uncharacterized protein LOC108415618, partial |
| gene-Cfus03920 | -- |
| gene-Cfus03921 | -- |
| gene-Cfus03922 | -- |
| gene-Cfus03923 | estrogen-related receptor gamma isoform X5 |
| gene-Cfus03924 | usherin |
| gene-Cfus03925 | BTB/POZ domain-containing protein KCTD3 |
| gene-Cfus03926 | receptor-type tyrosine-protein phosphatase epsilon-like |
| gene-Cfus03927 | uncharacterized protein C14orf28 homolog isoform X1 |
| gene-Cfus03928 | kelch-like protein 28 |
| gene-Cfus03929 | TOG array regulator of axonemal microtubules protein 1 |
| gene-Cfus03930 | Fanconi anemia group M protein |
| gene-Cfus03931 | mitochondrial basic amino acids transporter isoform X2 |
| gene-Cfus03932 | solute carrier family 25 member 47 isoform X2 |
| gene-Cfus03933 | tryptophan--tRNA ligase, cytoplasmic |
| gene-Cfus03934 | WD repeat-containing protein 25 |
| gene-Cfus03935 | brain-enriched guanylate kinase-associated protein isoform X1 |
| gene-Cfus03936 | brain-enriched guanylate kinase-associated protein-like |
| gene-Cfus03937 | protein delta homolog 1 isoform X1 |
| gene-Cfus03938 | cathepsin B-like, partial |
| gene-Cfus03939 | N-lysine methyltransferase SMYD2-A, partial |
| gene-Cfus03940 | prospero homeobox protein 1 isoform X1 |
| gene-Cfus03941 | -- |
| gene-Cfus03942 | proopiomelanocortin isoform X1 |
| gene-Cfus03943 | dnaJ homolog subfamily C member 27 |
| gene-Cfus03944 | xanthine dehydrogenase/oxidase, partial |
| gene-Cfus03945 | adenosine receptor A2a-like |
| gene-Cfus03946 | Golgi resident protein GCP60-like |
| gene-Cfus03947 | Golgi resident protein GCP60-like |
| gene-Cfus03948 | epithelial cell-transforming sequence 2 oncogene-like isoform X4 |
| gene-Cfus03949 | coiled-coil domain-containing protein 28A |
| gene-Cfus03950 | synaptosomal-associated protein 23-like isoform X1, partial |
| gene-Cfus03951 | ras and Rab interactor 3-like |
| gene-Cfus03952 | tuberoinfundibular peptide of 39 residues |
| gene-Cfus03953 | transmembrane protein 121-like |
| gene-Cfus03954 | uncharacterized protein C14orf80 homolog |
| gene-Cfus03955 | cyclin-dependent kinase 1, partial |
| gene-Cfus03956 | hypothetical protein PHYPO_G00019370 |
| gene-Cfus03957 | survival of motor neuron-related-splicing factor 30 |
| gene-Cfus03958 | cell cycle control protein 50B-like |
| gene-Cfus03959 | lysine-specific histone demethylase 1A isoform X1 |
| gene-Cfus03960 | leucine zipper protein 1 isoform X1 |
| gene-Cfus03961 | -- |
| gene-Cfus03962 | platelet-activating factor receptor |
| gene-Cfus03963 | Z-DNA binding protein kinase, partial |
| gene-Cfus03964 | uncharacterized protein sll0335-like, partial |
| gene-Cfus03965 | platelet-activating factor receptor |
| gene-Cfus03966 | parkin coregulated gene protein |
| gene-Cfus03967 | protein quaking-A isoform X2 |
| gene-Cfus03968 | centrosomal protein, partial |
| gene-Cfus03969 | gap junction beta-3 protein-like |
| gene-Cfus03970 | gap junction beta-3 protein-like |
| gene-Cfus03971 | zinc finger MYM-type protein 4-like isoform X3 |
| gene-Cfus03972 | UPF0568 protein C14orf166 homolog |
| gene-Cfus03973 | nidogen-2 isoform X1, partial |
| gene-Cfus03974 | prostaglandin E2 receptor EP2 subtype-like |
| gene-Cfus03975 | thioredoxin domain-containing protein 16 isoform X1 |
| gene-Cfus03976 | integral membrane protein GPR137C isoform X1 |
| gene-Cfus03977 | ERO1-like protein alpha |
| gene-Cfus03978 | protein Smaug 1 isoform X1, partial |
| gene-Cfus03979 | GTP cyclohydrolase 1 |
| gene-Cfus03980 | WD repeat and HMG-box DNA-binding protein 1 isoform X2 |
| gene-Cfus03981 | glucosamine 6-phosphate N-acetyltransferase |
| gene-Cfus03982 | proto-oncogene tyrosine-protein kinase ROS isoform X1 |
| gene-Cfus03983 | cardiac phospholamban |
| gene-Cfus03984 | protein FAM184A-like isoform X1 |
| gene-Cfus03985 | synaptotagmin-like protein 2 isoform X2 |
| gene-Cfus03986 | gap junction alpha-1 protein-like |
| gene-Cfus03987 | fatty acid-binding protein, brain |
| gene-Cfus03988 | titin-like isoform X2 |
| gene-Cfus03989 | tyrosine-protein kinase fyna |
| gene-Cfus03990 | Myristoylated alanine-rich C-kinase substrate |
| gene-Cfus03991 | collagen alpha-1(X) chain-like |
| gene-Cfus03992 | transforming growth factor-beta receptor-associated protein 1-like isoform X1 |
| gene-Cfus03993 | four and a half LIM domains protein 2-like |
| gene-Cfus03994 | alpha-(1,3)-fucosyltransferase 9 isoform X2 |
| gene-Cfus03995 | glycoprotein endo-alpha-1,2-mannosidase |
| gene-Cfus03996 | gap junction alpha-10 protein-like |
| gene-Cfus03997 | ras-related GTP-binding protein D-like isoform X2 |
| gene-Cfus03998 | gamma-aminobutyric acid receptor subunit rho-2-like isoform X1 |
| gene-Cfus03999 | nesprin-3-like isoform X1, partial |
| gene-Cfus04000 | calmin isoform X3 |
| gene-Cfus04001 | endoribonuclease Dicer |
| gene-Cfus04002 | homeobox protein goosecoid |
| gene-Cfus04003 | uncharacterized protein LOC105940582 |
| gene-Cfus04004 | leucine-rich repeat transmembrane protein FLRT2 |
| gene-Cfus04005 | -- |
| gene-Cfus04006 | ankyrin repeat and EF-hand domain-containing protein 1-like isoform X2 |
| gene-Cfus04007 | adenylate kinase 7-like isoform X2 |
| gene-Cfus04008 | galactocerebrosidase |
| gene-Cfus04009 | adenylate kinase 7-like |
| gene-Cfus04010 | adenylate kinase 7-like isoform X2 |
| gene-Cfus04011 | protein phosphatase 1 regulatory subunit 37-like isoform X2 |
| gene-Cfus04012 | protein phosphatase 1 regulatory subunit 37-like isoform X2 |
| gene-Cfus04013 | serine/threonine-protein kinase VRK1 |
| gene-Cfus04014 | -- |
| gene-Cfus04015 | B-cell lymphoma/leukemia 11B-like isoform X1 |
| gene-Cfus04016 | B-cell lymphoma/leukemia 11B-like isoform X2 |
| gene-Cfus04017 | actin-histidine N-methyltransferase |
| gene-Cfus04018 | coiled-coil domain-containing protein 85C-A |
| gene-Cfus04019 | HHIP-like protein 1 |
| gene-Cfus04020 | serine palmitoyltransferase 2, partial |
| gene-Cfus04021 | nucleic acid dioxygenase ALKBH1 |
| gene-Cfus04022 | SRA stem-loop-interacting RNA-binding protein, mitochondrial |
| gene-Cfus04023 | uncharacterized aarF domain-containing protein kinase 1 isoform X1 |
| gene-Cfus04024 | neurexin 3 |
| gene-Cfus04025 | type II iodothyronine deiodinase |
| gene-Cfus04026 | stonin-2 isoform X3 |
| gene-Cfus04027 | protein NRDE2 isoform X1, partial |
| gene-Cfus04028 | EF-hand calcium-binding domain-containing protein 2, partial |
| gene-Cfus04029 | kinesin-like protein KIF26B |
| gene-Cfus04030 | histone-lysine N-methyltransferase SMYD3, partial |
| gene-Cfus04031 | dimethyladenosine transferase 2, mitochondrial |
| gene-Cfus04032 | consortin isoform X2 |
| gene-Cfus04033 | protein ELYS |
| gene-Cfus04034 | ketohexokinase isoform X1 |
| gene-Cfus04035 | EMILIN-1-like isoform X1 |
| gene-Cfus04036 | GPN-loop GTPase 1, partial |
| gene-Cfus04037 | stathmin-4 |
| gene-Cfus04038 | interleukin-17F-like |
| gene-Cfus04039 | leucine-rich repeat-containing protein 57 |
| gene-Cfus04040 | breast cancer metastasis-suppressor 1-like protein-A |
| gene-Cfus04041 | ral GTPase-activating protein subunit alpha-1 isoform X4 |
| gene-Cfus04042 | insulinoma-associated protein 2 |
| gene-Cfus04043 | NF-kappa-B inhibitor alpha |
| gene-Cfus04044 | proteasome subunit alpha type-6 isoform X1 |
| gene-Cfus04045 | mitochondrial ribonuclease P protein 3 isoform X1 |
| gene-Cfus04046 | serine/threonine-protein phosphatase 2A regulatory subunit B'' subunit gamma |
| gene-Cfus04047 | protein FAM177A1 |
| gene-Cfus04048 | F-box only protein 33 |
| gene-Cfus04049 | cTAGE family member 5 isoform X1 |
| gene-Cfus04050 | gem-associated protein 2 |
| gene-Cfus04051 | pinin |
| gene-Cfus04052 | leucine-rich repeat and fibronectin type-III domain-containing protein 5-like |
| gene-Cfus04053 | piggyBac transposable element-derived protein 3-like |
| gene-Cfus04054 | myosin heavy chain, striated muscle-like isoform X5 |
| gene-Cfus04055 | JNK1/MAPK8-associated membrane protein |
| gene-Cfus04056 | probable G-protein coupled receptor 135 |
| gene-Cfus04057 | disheveled-associated activator of morphogenesis 1-like |
| gene-Cfus04058 | serine dehydratase-like isoform X1 |
| gene-Cfus04059 | apoptosis-stimulating of p53 protein 1-like isoform X3 |
| gene-Cfus04060 | protein tyrosine phosphatase type IVA 2a |
| gene-Cfus04061 | dimethyladenosine transferase 1, mitochondrial |
| gene-Cfus04062 | T-lymphoma invasion and metastasis-inducing protein 2 isoform X2 |
| gene-Cfus04063 | SR-related and CTD-associated factor 8 isoform X1 |
| gene-Cfus04064 | ensconsin-like |
| gene-Cfus04065 | ensconsin-like |
| gene-Cfus04066 | hypothetical protein PHYPO_G00020220 |
| gene-Cfus04067 | zinc finger protein, partial |
| gene-Cfus04068 | prepronociceptin-like |
| gene-Cfus04069 | spindle assembly checkpoint kinase-like |
| gene-Cfus04070 | kinesin-like protein KIF13B isoform X2 |
| gene-Cfus04071 | homeobox-containing protein 1-like isoform X6 |
| gene-Cfus04072 | potassium channel subfamily K member 13 |
| gene-Cfus04073 | tyrosyl-DNA phosphodiesterase 1 |
| gene-Cfus04074 | EF-hand calcium-binding domain-containing protein 11 isoform X2 |
| gene-Cfus04075 | forkhead box protein N3 isoform X2 |
| gene-Cfus04076 | stonin-2 isoform X3 |
| gene-Cfus04077 | tetratricopeptide repeat protein 7B |
| gene-Cfus04078 | ribosomal protein 63, mitochondrial |
| gene-Cfus04079 | protein FAM149B1 isoform X1 |
| gene-Cfus04080 | protein NDNF-like |
| gene-Cfus04081 | cytoplasmic dynein 1 heavy chain 1 isoform X1 |
| gene-Cfus04082 | WD repeat-containing protein 20, partial |
| gene-Cfus04083 | MAPK/MAK/MRK overlapping kinase isoform X1, partial |
| gene-Cfus04084 | nuclear export mediator factor NEMF isoform X1 |
| gene-Cfus04085 | protein kintoun, partial |
| gene-Cfus04086 | DNA polymerase epsilon subunit 2 isoform X1 |
| gene-Cfus04087 | uncharacterized protein LOC108270035 |
| gene-Cfus04088 | mis18-binding protein 1 |
| gene-Cfus04089 | HEAT repeat-containing protein 5A isoform X5 |
| gene-Cfus04090 | E3 ubiquitin-protein ligase HECTD1 isoform X2 |
| gene-Cfus04091 | AP-4 complex subunit sigma-1 |
| gene-Cfus04092 | striatin-3 isoform X1 |
| gene-Cfus04093 | cochlin |
| gene-Cfus04094 | sec1 family domain-containing protein 1 isoform X2 |
| gene-Cfus04095 | G2/M phase-specific E3 ubiquitin-protein ligase-like, partial |
| gene-Cfus04096 | -- |
| gene-Cfus04097 | serine/threonine-protein kinase D1 isoform X1 |
| gene-Cfus04098 | serine/threonine-protein kinase D1 isoform X3 |
| gene-Cfus04099 | forkhead box protein G1 |
| gene-Cfus04100 | spectrin beta chain, non-erythrocytic 5 isoform X1 |
| gene-Cfus04101 | REST corepressor 1 |
| gene-Cfus04102 | ankyrin repeat domain-containing protein 9 |
| gene-Cfus04103 | tectonin beta-propeller repeat-containing protein 2 |
| gene-Cfus04104 | bcl-2-modifying factor |
| gene-Cfus04105 | -- |
| gene-Cfus04106 | cysteine-rich motor neuron 1 protein-like |
| gene-Cfus04107 | eIF-2-alpha kinase GCN2 isoform X2 |
| gene-Cfus04108 | nuclear pore complex protein Nup133 |
| gene-Cfus04109 | rho GTPase-activating protein 11A isoform X1 |
| gene-Cfus04110 | gremlin-1-like |
| gene-Cfus04111 | formin-1-like isoform X1 |
| gene-Cfus04112 | ryanodine receptor 3 isoform X18 |
| gene-Cfus04113 | muscarinic acetylcholine receptor M5-like |
| gene-Cfus04114 | fibrinogen-like protein 1-like protein |
| gene-Cfus04115 | acid phosphatase type 7 isoform X1 |
| gene-Cfus04116 | thrombospondin-1-like |
| gene-Cfus04117 | fibrous sheath-interacting protein 1 isoform X1 |
| gene-Cfus04118 | cytochrome P450 1B1-like |
| gene-Cfus04119 | GTP cyclohydrolase 1 feedback regulatory protein isoform X1 |
| gene-Cfus04120 | dnaJ homolog subfamily C member 17 |
| gene-Cfus04121 | cdc42 effector protein 2-like |
| gene-Cfus04122 | dnaJ homolog subfamily C member 17 isoform X2 |
| gene-Cfus04123 | inverted formin-2-like |
| gene-Cfus04124 | adenylosuccinate synthetase isozyme 1 |
| gene-Cfus04125 | zinc finger and BTB domain-containing protein 42 |
| gene-Cfus04126 | unnamed protein product |
| gene-Cfus04127 | RAC-alpha serine/threonine-protein kinase-like isoform X2 |
| gene-Cfus04128 | centrosomal protein of 170 kDa protein B isoform X3 |
| gene-Cfus04129 | signal recognition particle 14 kDa protein |
| gene-Cfus04130 | x globin |
| gene-Cfus04131 | pleckstrin homology domain-containing family G member 3-like isoform X1 |
| gene-Cfus04132 | kunitz-type protease inhibitor 1 isoform X2 |
| gene-Cfus04133 | protein phosphatase 1 regulatory subunit 14D |
| gene-Cfus04134 | abscission/NoCut checkpoint regulator isoform X1 |
| gene-Cfus04135 | mitotic checkpoint serine/threonine-protein kinase BUB1 beta |
| gene-Cfus04136 | serine/threonine-protein kinase PAK 6-like |
| gene-Cfus04137 | ankyrin repeat domain-containing protein 63 |
| gene-Cfus04138 | 1-phosphatidylinositol 4,5-bisphosphate phosphodiesterase beta-2 |
| gene-Cfus04139 | hypothetical protein AMELA_G00122600 |
| gene-Cfus04140 | coiled-coil domain-containing protein 9B isoform X2 |
| gene-Cfus04141 | C-1-tetrahydrofolate synthase, cytoplasmic-like |
| gene-Cfus04142 | sushi domain-containing protein 6-like |
| gene-Cfus04143 | sodium/bile acid cotransporter-like |
| gene-Cfus04144 | protein max-like isoform X8 |
| gene-Cfus04145 | ras-related protein Rab-15-like isoform X2 |
| gene-Cfus04146 | glutathione peroxidase 2 |
| gene-Cfus04147 | signal-induced proliferation-associated 1-like protein 1 isoform X1 |
| gene-Cfus04148 | zinc finger protein DPF3-like isoform X1 |
| gene-Cfus04149 | RAS guanyl-releasing protein 1-like |
| gene-Cfus04150 | RAS guanyl-releasing protein 1-like, partial |
| gene-Cfus04151 | 14-3-3 protein beta/alpha-like |
| gene-Cfus04152 | grainyhead-like protein 1 homolog isoform X2 |
| gene-Cfus04153 | Krueppel-like factor 11 |
| gene-Cfus04154 | putative pre-mRNA-splicing factor ATP-dependent RNA helicase DHX32 |
| gene-Cfus04155 | fibronectin type 3 and ankyrin repeat domains protein 1 |
| gene-Cfus04156 | disintegrin and metalloproteinase domain-containing protein 12-like isoform X2 |
| gene-Cfus04157 | centrosomal protein C10orf90 homolog |
| gene-Cfus04158 | protein-lysine N-methyltransferase METTL10 isoform X1 |
| gene-Cfus04159 | BRISC complex subunit Abro1 isoform X1 |
| gene-Cfus04160 | rho GTPase-activating protein 5 |
| gene-Cfus04161 | iron-sulfur protein NUBPL |
| gene-Cfus04162 | probable D-tyrosyl-tRNA(Tyr) deacylase 2 isoform X2 |
| gene-Cfus04163 | hypothetical protein PHYPO_G00017720 |
| gene-Cfus04164 | protein O-mannosyl-transferase 2 isoform X1, partial |
| gene-Cfus04165 | protein lin-52 homolog |
| gene-Cfus04166 | visual system homeobox 2 isoform X2 |
| gene-Cfus04167 | zinc finger protein, partial |
| gene-Cfus04168 | cyclin-dependent kinase 2-interacting protein isoform X2 |
| gene-Cfus04169 | uncharacterized protein LOC107722728 |
| gene-Cfus04170 | ADP-ribose glycohydrolase ARH3 |
| gene-Cfus04171 | HAUS augmin-like complex subunit 2 |
| gene-Cfus04172 | HEAT repeat-containing protein 4 isoform X2 |
| gene-Cfus04173 | transmembrane protein 151B-like |
| gene-Cfus04174 | RNA polymerase II-associated protein 1 |
| gene-Cfus04175 | leukocyte tyrosine kinase receptor isoform X2 |
| gene-Cfus04176 | inositol-trisphosphate 3-kinase A isoform X1 |
| gene-Cfus04177 | isovaleryl-CoA dehydrogenase, mitochondrial |
| gene-Cfus04178 | bromo adjacent homology domain-containing 1 protein |
| gene-Cfus04179 | prolactin-releasing peptide receptor-like |
| gene-Cfus04180 | uncharacterized protein C15orf57 homolog |
| gene-Cfus04181 | RNA pseudouridylate synthase domain-containing protein 2 |
| gene-Cfus04182 | protein dispatched homolog 2 isoform X2 |
| gene-Cfus04183 | extracellular tyrosine-protein kinase PKDCC-like |
| gene-Cfus04184 | echinoderm microtubule-associated protein-like 1 isoform X4 |
| gene-Cfus04185 | echinoderm microtubule-associated protein-like 1 isoform X4 |
| gene-Cfus04186 | ena/VASP-like protein isoform X1 |
| gene-Cfus04187 | sphingolipid delta(4)-desaturase/C4-monooxygenase DES2 |
| gene-Cfus04188 | transcriptional repressor protein YY1a |
| gene-Cfus04189 | tripeptidyl-peptidase 1 |
| gene-Cfus04190 | growth/differentiation factor 6-A-like |
| gene-Cfus04191 | apolipoprotein, partial |
| gene-Cfus04192 | uncharacterized protein C1orf115 homolog |
| gene-Cfus04193 | SH3 domain-containing YSC84-like protein 1, partial |
| gene-Cfus04194 | SH3 domain-containing YSC84-like protein 1 |
| gene-Cfus04195 | NHS-like protein 1 isoform X3 |
| gene-Cfus04196 | NHS-like protein 1 isoform X1 |
| gene-Cfus04197 | DNA-binding protein inhibitor ID-2a |
| gene-Cfus04198 | kinase D-interacting substrate of 220 kDa B isoform X2 |
| gene-Cfus04199 | membrane-bound O-acyltransferase domain-containing protein 2-like |
| gene-Cfus04200 | arf-GAP with SH3 domain, ANK repeat and PH domain-containing protein 2 isoform X5 |
| gene-Cfus04201 | Integrin beta-1-binding protein 1 |
| gene-Cfus04202 | isoamyl acetate-hydrolyzing esterase 1 homolog |
| gene-Cfus04203 | disintegrin and metalloproteinase domain-containing protein 17-like isoform X1 |
| gene-Cfus04204 | E3 ubiquitin-protein ligase RNF144A |
| gene-Cfus04205 | radical S-adenosyl methionine domain-containing protein 2 |
| gene-Cfus04206 | UMP-CMP kinase 2, mitochondrial, partial |
| gene-Cfus04207 | -- |
| gene-Cfus04208 | transcription factor SOX-11-like |
| gene-Cfus04209 | collectin-11 isoform X2 |
| gene-Cfus04210 | interphotoreceptor matrix proteoglycan 1-like |
| gene-Cfus04211 | unconventional myosin-VI |
| gene-Cfus04212 | unconventional myosin-VI, partial |
| gene-Cfus04213 | hypothetical protein AMELA_G00123560 |
| gene-Cfus04214 | DNA (cytosine-5)-methyltransferase 3A-like isoform X1 |
| gene-Cfus04215 | putative Polycomb group protein ASXL2 isoform X2 |
| gene-Cfus04216 | kinesin-like protein KIF3B |
| gene-Cfus04217 | zinc transporter 1-like |
| gene-Cfus04218 | trifunctional enzyme subunit alpha, mitochondrial |
| gene-Cfus04219 | ras-related protein Rab-10 |
| gene-Cfus04220 | transmembrane protein 62 |
| gene-Cfus04221 | prostaglandin reductase 2 isoform X1 |
| gene-Cfus04222 | ELM2 and SANT domain-containing protein 1 |
| gene-Cfus04223 | cysteine-rich protein 1 |
| gene-Cfus04224 | metastasis-associated protein MTA1 isoform X1, partial |
| gene-Cfus04225 | transmembrane protein 229b |
| gene-Cfus04226 | zinc finger protein 36, C3H1 type-like 1 |
| gene-Cfus04227 | pleckstrin homology domain-containing family H member 1-like isoform X2 |
| gene-Cfus04228 | mitochondrial 2-oxodicarboxylate carrier isoform X1 |
| gene-Cfus04229 | paired box protein Pax-9 |
| gene-Cfus04230 | homeobox protein Nkx-2.8 |
| gene-Cfus04231 | MAP3K12-binding inhibitory protein 1-like, partial |
| gene-Cfus04232 | ribosomal protein S6 kinase alpha-5 |
| gene-Cfus04233 | codanin-1 |
| gene-Cfus04234 | stAR-related lipid transfer protein 9 isoform X2 |
| gene-Cfus04235 | spectrin beta chain, erythrocytic-like isoform X1 |
| gene-Cfus04236 | protein Daple-like isoform X1 |
| gene-Cfus04237 | ovarian cancer G-protein coupled receptor 1 |
| gene-Cfus04238 | sodium/potassium/calcium exchanger 4-like isoform X1 |
| gene-Cfus04239 | cysteine-rich motor neuron 1 protein isoform X2 |
| gene-Cfus04240 | Cysteine-rich motor neuron 1 protein, partial |
| gene-Cfus04241 | cysteine-rich motor neuron 1 protein-like |
| gene-Cfus04242 | proton-coupled folate transporter-like |
| gene-Cfus04243 | -- |
| gene-Cfus04244 | bifunctional glutamate/proline--tRNA ligase isoform X2 |
| gene-Cfus04245 | uncharacterized protein LOC108269466 |
| gene-Cfus04246 | probable G-protein coupled receptor 132 |
| gene-Cfus04247 | activator of 90 kDa heat shock protein ATPase homolog 1 |
| gene-Cfus04248 | putative JmjC domain-containing histone demethylation protein 2C isoform X2, partial |
| gene-Cfus04249 | early growth response protein 2b-like |
| gene-Cfus04250 | rhotekin-2 isoform X1 |
| gene-Cfus04251 | cilia- and flagella-associated protein 99 isoform X1 |
| gene-Cfus04252 | lateral signaling target protein 2 isoform X1 |
| gene-Cfus04253 | cytosolic 5'-nucleotidase 1A-like |
| gene-Cfus04254 | 39S ribosomal protein L35, mitochondrial-like |
| gene-Cfus04255 | charged multivesicular body protein 3 |
| gene-Cfus04256 | heat shock 70 kDa protein 4L |
| gene-Cfus04257 | heat shock 70 kDa protein 4L |
| gene-Cfus04258 | echinoderm microtubule-associated protein-like 5 isoform X2 |
| gene-Cfus04259 | iron-sulfur cluster assembly 2 homolog, mitochondrial isoform X1 |
| gene-Cfus04260 | epididymal secretory protein E1 isoform X2 |
| gene-Cfus04261 | GSK3-beta interaction protein |
| gene-Cfus04262 | autophagy-related protein 2 B isoform X1, partial |
| gene-Cfus04263 | mutS protein homolog 4 isoform X3 |
| gene-Cfus04264 | kinectin isoform X1 |
| gene-Cfus04265 | hypothetical protein AMELA_G00124170 |
| gene-Cfus04266 | transmembrane protein 260 isoform X1 |
| gene-Cfus04267 | homeobox protein OTX2 |
| gene-Cfus04268 | exocyst complex component 5 isoform X1 |
| gene-Cfus04269 | AP-5 complex subunit mu-1 |
| gene-Cfus04270 | N-alpha-acetyltransferase 30-like |
| gene-Cfus04271 | uncharacterized protein LOC113538703 |
| gene-Cfus04272 | solute carrier family 35 member F4 isoform X1 |
| gene-Cfus04273 | placenta growth factor |
| gene-Cfus04274 | palmitoyltransferase ZDHHC22 isoform X2 |
| gene-Cfus04275 | calcium permeable stress-gated cation channel 1 isoform X1 |
| gene-Cfus04276 | neuroglobin |
| gene-Cfus04277 | protein FAM, partial |
| gene-Cfus04278 | ubiquinone biosynthesis monooxygenase COQ6, mitochondrial |
| gene-Cfus04279 | ectonucleoside triphosphate diphosphohydrolase 5 |
| gene-Cfus04280 | basal body-orientation factor 1 |
| gene-Cfus04281 | methylmalonate-semialdehyde dehydrogenase [acylating], mitochondrial |
| gene-Cfus04282 | maleylacetoacetate isomerase isoform X5 |
| gene-Cfus04283 | protein TMED8 isoform X1 |
| gene-Cfus04284 | tau-tubulin kinase 2 isoform X2 |
| gene-Cfus04285 | THAP domain-containing protein 6-like |
| gene-Cfus04286 | calpain-3-like isoform X2 |
| gene-Cfus04287 | zinc finger protein 106 |
| gene-Cfus04288 | neudesin |
| gene-Cfus04289 | T-cell activation Rho GTPase-activating protein-like |
| gene-Cfus04290 | ezrin-like |
| gene-Cfus04291 | potassium channel subfamily K member 3-like |
| gene-Cfus04292 | mitochondrial mosc domain-containing protein 1 |
| gene-Cfus04293 | sushi domain-containing protein 4-like |
| gene-Cfus04294 | fibrillin-1-like |
| gene-Cfus04295 | fibrillin-1-like |
| gene-Cfus04296 | complement component C1q receptor |
| gene-Cfus04297 | calpain-1 catalytic subunit-like |
| gene-Cfus04298 | connector enhancer of kinase suppressor of ras 3-like isoform X3 |
| gene-Cfus04299 | connector enhancer of kinase suppressor of ras 3-like isoform X1, partial |
| gene-Cfus04300 | glutaminyl-peptide cyclotransferase isoform X1 |
| gene-Cfus04301 | adenylyltransferase and sulfurtransferase MOCS3 |
| gene-Cfus04302 | serine/threonine-protein kinase D3 isoform X2 |
| gene-Cfus04303 | hepatocyte nuclear factor 3-beta |
| gene-Cfus04304 | paired box protein Pax-1 |
| gene-Cfus04305 | homeobox protein Nkx-2.2 |
| gene-Cfus04306 | thyroid transcription factor 1-like |
| gene-Cfus04307 | centrosomal protein kizuna isoform X3 |
| gene-Cfus04308 | ral GTPase-activating protein subunit alpha-2 isoform X3 |
| gene-Cfus04309 | insulinoma-associated protein 1 |
| gene-Cfus04310 | collagen alpha-1(XII) chain-like isoform X4, partial |
| gene-Cfus04311 | cytochrome c oxidase subunit 7A2, mitochondrial isoform X2 |
| gene-Cfus04312 | cell cycle control protein 50A-like |
| gene-Cfus04313 | cell cycle control protein 50A-like |
| gene-Cfus04314 | filamin-A-interacting protein 1-like isoform X3 |
| gene-Cfus04315 | transmembrane protein 87A-like |
| gene-Cfus04316 | vam6/Vps39-like protein isoform X3 |
| gene-Cfus04317 | polypeptide N-acetylgalactosaminyltransferase 16 isoform X1 |
| gene-Cfus04318 | protein numb homolog isoform X1 |
| gene-Cfus04319 | exonuclease 3'-5' domain-containing protein 2 isoform X1, partial |
| gene-Cfus04320 | glioma tumor suppressor candidate region gene 1 protein-like |
| gene-Cfus04321 | enhancer of rudimentary homolog |
| gene-Cfus04322 | E3 ubiquitin-protein ligase UBR1 isoform X1 |
| gene-Cfus04323 | photoreceptor outer segment membrane glycoprotein 2-like |
| gene-Cfus04324 | transmembrane protein 179 |
| gene-Cfus04325 | uncharacterized protein LOC108270437 isoform X3 |
| gene-Cfus04326 | kinesin-like protein KIF26A isoform X3 |
| gene-Cfus04327 | kinesin-like protein KIF26A isoform X1 |
| gene-Cfus04328 | 60 kDa lysophospholipase isoform X1 |
| gene-Cfus04329 | ADP-ribosylation factor 6 isoform X1 |
| gene-Cfus04330 | protein-lysine methyltransferase METTL21D |
| gene-Cfus04331 | erythroid differentiation-related factor 1 |
| gene-Cfus04332 | tonsoku-like protein, partial |
| gene-Cfus04333 | NK-lysin type 3 precursor |
| gene-Cfus04334 | 26S protease regulatory subunit 4 |
| gene-Cfus04335 | ubiquitin thioesterase zranb1-B isoform X2 |
| gene-Cfus04336 | A-kinase anchor protein 6 |
| gene-Cfus04337 | neuronal PAS domain-containing protein 3 isoform X1 |
| gene-Cfus04338 | neuronal PAS domain-containing protein 3 [Thamnophis sirtalis] |
| gene-Cfus04339 | neuronal PAS domain-containing protein 3 isoform X1 |
| gene-Cfus04340 | hypothetical protein cypCar_00008760 |
| gene-Cfus04341 | serine palmitoyltransferase small subunit A |
| gene-Cfus04342 | E2F-associated phosphoprotein |
| gene-Cfus04343 | sorting nexin-6 |
| gene-Cfus04344 | bromodomain adjacent to zinc finger domain protein 1A isoform X5 |
| gene-Cfus04345 | signal recognition particle 54 kDa protein |
| gene-Cfus04346 | protein transport protein Sec23A isoform X2 |
| gene-Cfus04347 | C-type lectin domain family 14 member A |
| gene-Cfus04348 | somatostatin receptor type 1 |
| gene-Cfus04349 | hepatocyte nuclear factor 3-alpha |
| gene-Cfus04350 | mirror-image polydactyly gene 1 protein isoform X1 |
| gene-Cfus04351 | tyrosine-protein kinase receptor TYRO3 isoform X2 |
| gene-Cfus04352 | MAX gene-associated protein-like isoform X1, partial |
| gene-Cfus04353 | mitogen-activated protein kinase-binding protein 1-like isoform X3 |
| gene-Cfus04354 | bifunctional peptidase and (3S)-lysyl hydroxylase JMJD7 isoform X2 |
| gene-Cfus04355 | beclin 1-associated autophagy-related key regulator |
| gene-Cfus04356 | F-box only protein 34 |
| gene-Cfus04357 | galectin-3 |
| gene-Cfus04358 | dapper homolog 1 |
| gene-Cfus04359 | protein TALPID3 isoform X4 |
| gene-Cfus04360 | hypothetical protein PHYPO_G00015480 |
| gene-Cfus04361 | AT-rich interactive domain-containing protein 4A-like, partial |
| gene-Cfus04362 | ADP-dependent glucokinase |
| gene-Cfus04363 | C-terminal-binding protein 2 |
| gene-Cfus04364 | frizzled-3b isoform X1 |
| gene-Cfus04365 | serine/threonine-protein kinase MRCK alpha isoform X7 |
| gene-Cfus04366 | left-right determination factor 1-like |
| gene-Cfus04367 | jouberin isoform X3 |
| gene-Cfus04368 | MFS-type transporter SLC18B1 isoform X1, partial |
| gene-Cfus04369 | tubby-related protein 4 isoform X1 |
| gene-Cfus04370 | myc target protein 1 homolog |
| gene-Cfus04371 | nesprin-1 isoform X4, partial |
| gene-Cfus04372 | coiled-coil domain-containing protein, partial |
| gene-Cfus04373 | required for meiotic nuclear division protein 1 homolog |
| gene-Cfus04374 | A-kinase anchor protein 12-like |
| gene-Cfus04375 | syntaxin-11 |
| gene-Cfus04376 | utrophin isoform X1 |
| gene-Cfus04377 | F-box only protein 30a |
| gene-Cfus04378 | pancreatic secretory granule membrane major glycoprotein GP2-like |
| gene-Cfus04379 | E3 ubiquitin-protein ligase SHPRH, partial |
| gene-Cfus04380 | metabotropic glutamate receptor 1 isoform X1 |
| gene-Cfus04381 | syntaxin-binding protein 5 isoform X5 |
| gene-Cfus04382 | SAM and SH3 domain-containing protein 1-like isoform X1 |
| gene-Cfus04383 | D-aspartate oxidase |
| gene-Cfus04384 | zinc finger and BTB domain-containing protein 24 isoform X2 |
| gene-Cfus04385 | forkhead box protein O3-like |
| gene-Cfus04386 | lactation elevated protein 1-like isoform X2 |
| gene-Cfus04387 | visinin-like protein 1 |
| gene-Cfus04388 | structural maintenance of chromosomes protein 6 |
| gene-Cfus04389 | sodium-dependent multivitamin transporter-like |
| gene-Cfus04390 | quinone oxidoreductase PIG3 |
| gene-Cfus04391 | dnaJ homolog subfamily C member 5-like |
| gene-Cfus04392 | atypical kinase ADCK3, mitochondrial-like isoform X1 |
| gene-Cfus04393 | gap junction epsilon-1 protein-like |
| gene-Cfus04394 | beta-taxilin-like |
| gene-Cfus04395 | dual specificity protein phosphatase 23 |
| gene-Cfus04396 | transcriptional-regulating factor 1-like isoform X1 |
| gene-Cfus04397 | protein-tyrosine kinase 2-beta-like isoform X1 |
| gene-Cfus04398 | neuronal acetylcholine receptor subunit alpha-2-like |
| gene-Cfus04399 | bifunctional epoxide hydrolase 2, partial |
| gene-Cfus04400 | phospholipase A and acyltransferase 2 |
| gene-Cfus04401 | zinc finger protein 513-like |
| gene-Cfus04402 | tyrosine-protein kinase SRK3-like isoform X1 |
| gene-Cfus04403 | retinitis pigmentosa 1-like 1 protein |
| gene-Cfus04404 | C8orf74-like protein, partial |
| gene-Cfus04405 | XK-related protein 6 |
| gene-Cfus04406 | protein FAM167A-like |
| gene-Cfus04407 | bis(5'-adenosyl)-triphosphatase enpp4 |
| gene-Cfus04408 | chloride intracellular channel protein 5 isoform X1 |
| gene-Cfus04409 | runt-related transcription factor 2 isoform X1 |
| gene-Cfus04410 | transcription initiation protein SPT3 homolog |
| gene-Cfus04411 | cell division cycle 5-like protein isoform X1 |
| gene-Cfus04412 | kelch-like protein 29 |
| gene-Cfus04413 | tribbles homolog 2-like |
| gene-Cfus04414 | DNA helicase MCM8, partial |
| gene-Cfus04415 | cardiolipin synthase (CMP-forming) |
| gene-Cfus04416 | protein DVR-1 |
| gene-Cfus04417 | hypothetical protein AMELA_G00126010 |
| gene-Cfus04418 | hydroxyacid oxidase 1 |
| gene-Cfus04419 | thioredoxin-related transmembrane protein 4 isoform X1 |
| gene-Cfus04420 | 1-phosphatidylinositol 4,5-bisphosphate phosphodiesterase beta-1 isoform X2 |
| gene-Cfus04421 | 1-phosphatidylinositol 4,5-bisphosphate phosphodiesterase beta-4-like isoform X1, partial |
| gene-Cfus04422 | gamma-2-syntrophin isoform X2 |
| gene-Cfus04423 | transmembrane protein 151B-like |
| gene-Cfus04424 | B2 bradykinin receptor-like |
| gene-Cfus04425 | B2 bradykinin receptor-like |
| gene-Cfus04426 | potassium channel subfamily K member 10 isoform X2, partial |
| gene-Cfus04427 | tyrosine-protein phosphatase non-receptor type 21 isoform X2, partial |
| gene-Cfus04428 | tyrosine-protein phosphatase non-receptor type 21 isoform X1 |
| gene-Cfus04429 | akirin-2 |
| gene-Cfus04430 | zinc finger protein 292 |
| gene-Cfus04431 | RecName: Full=Glycoprotein hormones alpha chain; AltName: Full=GTH-alpha; AltName: Full=Gonadotropin alpha chain; Flags: Precursor |
| gene-Cfus04432 | heterogeneous nuclear ribonucleoprotein Q isoform X2 |
| gene-Cfus04433 | sorting nexin-14 isoform X7 |
| gene-Cfus04434 | otoferlin-like isoform X2 |
| gene-Cfus04435 | hypothetical protein PHYPO_G00014570 |
| gene-Cfus04436 | phospholipase DDHD1-like isoform X2 |
| gene-Cfus04437 | fermitin family homolog 2 isoform X4 |
| gene-Cfus04438 | syntaxin-binding protein 6 |
| gene-Cfus04439 | syntaxin-binding protein 6 isoform X1 |
| gene-Cfus04440 | gap junction delta-2 protein |
| gene-Cfus04441 | intron-binding protein aquarius isoform X4 |
| gene-Cfus04442 | diphthine--ammonia ligase |
| gene-Cfus04443 | -- |
| gene-Cfus04444 | uncharacterized protein C15orf41 homolog isoform X1 |
| gene-Cfus04445 | homeobox protein Meis2 isoform X9 |
| gene-Cfus04446 | -- |
| gene-Cfus04447 | sprouty-related, EVH1 domain-containing protein 1 |
| gene-Cfus04448 | protein FAM98B isoform X3 |
| gene-Cfus04449 | gamma-aminobutyric acid receptor subunit rho-1-like |
| gene-Cfus04450 | ATP-dependent RNA helicase DDX24 |
| gene-Cfus04451 | ubiquitin thioesterase OTUB2 |
| gene-Cfus04452 | coiled-coil domain-containing protein 42 homolog isoform X1 |
| gene-Cfus04453 | ankyrin repeat and SOCS box protein 2b isoform X1 |
| gene-Cfus04454 | protein FAM181A |
| gene-Cfus04455 | proline-rich membrane anchor 1-like |
| gene-Cfus04456 | protein unc-79 homolog isoform X2 |
| gene-Cfus04457 | hypothetical protein PHYPO_G00014350 |
| gene-Cfus04458 | BTB/POZ domain-containing protein 7 |
| gene-Cfus04459 | putative E3 ubiquitin-protein ligase UBR7, partial |
| gene-Cfus04460 | tetratricopeptide repeat protein 8 isoform X1 |
| gene-Cfus04461 | kinetochore protein NDC80, partial |
| gene-Cfus04462 | serine/threonine-protein kinase Nek9 |
| gene-Cfus04463 | acylphosphatase-1 |
| gene-Cfus04464 | golgin subfamily A member 6-like protein 1 |
| gene-Cfus04465 | acylphosphatase-1 |
| gene-Cfus04466 | proto-oncogene c-Fos-like |
| gene-Cfus04467 | jun dimerization protein 2 isoform X2 |
| gene-Cfus04468 | tubulin polyglutamylase TTLL5 isoform X2 |
| gene-Cfus04469 | transforming growth factor beta-3 |
| gene-Cfus04470 | intraflagellar transport protein 43 homolog |
| gene-Cfus04471 | hypothetical protein PHYPO_G00014170 |
| gene-Cfus04472 | steroid hormone receptor ERR2 isoform X2 |
| gene-Cfus04473 | steroid hormone receptor ERR2-like [Notothenia coriiceps] |
| gene-Cfus04474 | prospero homeobox protein 2 isoform X1 |
| gene-Cfus04475 | dihydrolipoyllysine-residue succinyltransferase component of 2-oxoglutarate dehydrogenase complex, mitochondrial |
| gene-Cfus04476 | ribosomal protein S6 kinase-like 1 isoform X1 |
| gene-Cfus04477 | olfactomedin-4-like |
| gene-Cfus04478 | uncharacterized protein LOC101734515 |
| gene-Cfus04479 | olfactomedin-4-like |
| gene-Cfus04480 | leucine-rich repeat-containing protein 74A |
| gene-Cfus04481 | protein angel homolog 1 |
| gene-Cfus04482 | tubulinyl-Tyr carboxypeptidase 1 |
| gene-Cfus04483 | apoptosis-resistant E3 ubiquitin protein ligase 1 isoform X5 |
| gene-Cfus04484 | latent-transforming growth factor beta-binding protein 2 |
| gene-Cfus04485 | HCLS1-binding protein 3 |
| gene-Cfus04486 | Rho-related GTP-binding protein RhoB |
| gene-Cfus04487 | unnamed protein product |
| gene-Cfus04488 | b(0,+)-type amino acid transporter 1-like |
| gene-Cfus04489 | b(0,+)-type amino acid transporter 1-like isoform X2 |
| gene-Cfus04490 | syndecan-1 isoform X2, partial |
| gene-Cfus04491 | hippocalcin-like protein 1 |
| gene-Cfus04492 | ornithine decarboxylase, partial |
| gene-Cfus04493 | ornithine decarboxylase |
| gene-Cfus04494 | zinc finger protein 850-like isoform X2 |
| gene-Cfus04495 | potassium voltage-gated channel subfamily H member 1 isoform X2 |
| gene-Cfus04496 | potassium voltage-gated channel subfamily H member 1a isoform X6 |
| gene-Cfus04497 | protein-cysteine N-palmitoyltransferase HHAT isoform X1 |
| gene-Cfus04498 | acetyl-coenzyme A synthetase 2-like, mitochondrial, partial |
| gene-Cfus04499 | hypothetical protein PHYPO_G00011550 |
| gene-Cfus04500 | solute carrier family 22 member 7-like, partial |
| gene-Cfus04501 | hypothetical protein F2P81_004927 [Scophthalmus maximus] |
| gene-Cfus04502 | nephrocystin-1 |
| gene-Cfus04503 | myelin and lymphocyte protein-like |
| gene-Cfus04504 | myelin and lymphocyte protein-like |
| gene-Cfus04505 | 28S ribosomal protein S5, mitochondrial |
| gene-Cfus04506 | sodium/calcium exchanger 1-like isoform X1 |
| gene-Cfus04507 | ectodysplasin-A receptor-associated adapter protein |
| gene-Cfus04508 | neuroblast differentiation-associated protein AHNAK-like |
| gene-Cfus04509 | exonuclease 1-like, partial |
| gene-Cfus04510 | protein YIPF4 isoform X1 |
| gene-Cfus04511 | baculoviral IAP repeat-containing protein 6 |
| gene-Cfus04512 | tetratricopeptide repeat protein 27 |
| gene-Cfus04513 | latent-transforming growth factor beta-binding protein 1 |
| gene-Cfus04514 | Ras guanyl-releasing protein 3 |
| gene-Cfus04515 | protein FAM98A |
| gene-Cfus04516 | zinc finger protein, partial |
| gene-Cfus04517 | TBC1 domain family member 12-like, partial |
| gene-Cfus04518 | D(1)-like dopamine receptor |
| gene-Cfus04519 | fibroblast growth factor-binding protein 2-like |
| gene-Cfus04520 | RecName: Full=Protein phosphatase 1 regulatory subunit 3C |
| gene-Cfus04521 | polycomb group RING finger protein 5-A |
| gene-Cfus04522 | ankyrin repeat domain-containing protein 1-like |
| gene-Cfus04523 | hypothetical protein PHYPO_G00011830 |
| gene-Cfus04524 | 5-hydroxytryptamine receptor 7-like |
| gene-Cfus04525 | pantothenate kinase 1-like isoform X3 |
| gene-Cfus04526 | tumor necrosis factor receptor superfamily member 6 |
| gene-Cfus04527 | phosphatidylinositol 3,4,5-trisphosphate 3-phosphatase and dual-specificity protein phosphatase PTEN-like |
| gene-Cfus04528 | multiple inositol polyphosphate phosphatase 1-like |
| gene-Cfus04529 | phosphatidylcholine:ceramide cholinephosphotransferase 1-like, partial |
| gene-Cfus04530 | homeobox protein HMX2-like |
| gene-Cfus04531 | homeobox protein HMX3-like |
| gene-Cfus04532 | zinc finger protein Pegasus-like |
| gene-Cfus04533 | -- |
| gene-Cfus04534 | serine protease HTRA1A-like |
| gene-Cfus04535 | wings apart-like protein homolog |
| gene-Cfus04536 | piggyBac transposable element-derived protein 4-like |
| gene-Cfus04537 | glutamate receptor ionotropic, delta-1-like |
| gene-Cfus04538 | glutamate receptor ionotropic, delta-1-like |
| gene-Cfus04539 | phospholipase B1, membrane-associated-like |
| gene-Cfus04540 | Serine/threonine-protein phosphatase PP1-beta catalytic subunit |
| gene-Cfus04541 | rab3 GTPase-activating protein non-catalytic subunit isoform X6 |
| gene-Cfus04542 | isoleucine--tRNA ligase, mitochondrial isoform X2 |
| gene-Cfus04543 | -- |
| gene-Cfus04544 | hypothetical protein AMELA_G00129090 |
| gene-Cfus04545 | glutamyl-tRNA(Gln) amidotransferase subunit A, mitochondrial |
| gene-Cfus04546 | reticulon-4-interacting protein 1, mitochondrial |
| gene-Cfus04547 | absent in melanoma 1 protein-like isoform X2, partial |
| gene-Cfus04548 | uncharacterized protein LOC106526271 |
| gene-Cfus04549 | absent in melanoma 1 protein isoform X1 |
| gene-Cfus04550 | chloride intracellular channel protein 4 isoform X1 |
| gene-Cfus04551 | serine/arginine repetitive matrix protein 1-like isoform X1, partial |
| gene-Cfus04552 | myotubularin-related protein 9-like |
| gene-Cfus04553 | lectin BRA-3-like isoform X1 |
| gene-Cfus04554 | lectin BRA-3-like |
| gene-Cfus04555 | hypothetical protein AMELA_G00128960 |
| gene-Cfus04556 | tyrosine-protein kinase Lck-like |
| gene-Cfus04557 | transmembrane protein 54-like |
| gene-Cfus04558 | zona pellucida protein C |
| gene-Cfus04559 | Polyadenylate-binding protein 4 |
| gene-Cfus04560 | hypothetical protein PHYPO_G00012200 |
| gene-Cfus04561 | rho guanine nucleotide exchange factor 10 isoform X1 |
| gene-Cfus04562 | protein CLN8 |
| gene-Cfus04563 | disks large-associated protein 2 isoform X1 |
| gene-Cfus04564 | disks large-associated protein 2 |
| gene-Cfus04565 | hypothetical protein cypCar_00027925 |
| gene-Cfus04566 | glutamate-rich protein 1-like, partial |
| gene-Cfus04567 | UDP-glucose 4-epimerase isoform X1 |
| gene-Cfus04568 | zinc finger and BTB domain-containing protein 8A |
| gene-Cfus04569 | hydroxymethylglutaryl-CoA lyase, mitochondrial |
| gene-Cfus04570 | gap junction beta-3 protein-like |
| gene-Cfus04571 | gap junction beta-4 protein-like |
| gene-Cfus04572 | WD repeat-containing protein 43 |
| gene-Cfus04573 | tRNA (adenine(58)-N(1))-methyltransferase, mitochondrial |
| gene-Cfus04574 | speedy protein A isoform X2 |
| gene-Cfus04575 | hypothetical protein cypCar_00000531 |
| gene-Cfus04576 | SH3 and cysteine-rich domain-containing protein |
| gene-Cfus04577 | prosaposin-like |
| gene-Cfus04578 | prosaposin-like |
| gene-Cfus04579 | prosaposin-like |
| gene-Cfus04580 | prosaposin-like |
| gene-Cfus04581 | hypothetical protein PHYPO_G00012410 |
| gene-Cfus04582 | mitochondrial fission regulator 1-like |
| gene-Cfus04583 | oxidation resistance protein 1-like |
| gene-Cfus04584 | selenoprotein N |
| gene-Cfus04585 | selenoprotein N |
| gene-Cfus04586 | zinc finger protein 593 isoform X1 |
| gene-Cfus04587 | rho guanine nucleotide exchange factor 33 isoform X1 |
| gene-Cfus04588 | mitogen-activated protein kinase kinase kinase kinase 3 isoform X1 |
| gene-Cfus04589 | mitogen-activated protein kinase kinase kinase kinase 3 isoform X2, partial |
| gene-Cfus04590 | hypothetical protein PHYPO_G00012530 |
| gene-Cfus04591 | hypothetical protein cypCar_00011041 |
| gene-Cfus04592 | prolyl 4-hydroxylase subunit alpha-1-like isoform X1, partial |
| gene-Cfus04593 | alpha-actinin-2 |
| gene-Cfus04594 | ryanodine receptor 2-like |
| gene-Cfus04595 | ryanodine receptor 2-like |
| gene-Cfus04596 | hypothetical protein cypCar_00027925 |
| gene-Cfus04597 | muscarinic acetylcholine receptor M1-like |
| gene-Cfus04598 | 52 kDa repressor of the inhibitor of the protein kinase |
| gene-Cfus04599 | gremlin-2-like |
| gene-Cfus04600 | hypothetical protein PHYPO_G00012600 |
| gene-Cfus04601 | receptor expression-enhancing protein 3a |
| gene-Cfus04602 | cytochrome P450 26B1-like |
| gene-Cfus04603 | interferon-induced very large GTPase 1-like isoform X2 |
| gene-Cfus04604 | up-regulator of cell proliferation-like |
| gene-Cfus04605 | interferon-induced very large GTPase 1-like isoform X2 |
| gene-Cfus04606 | up-regulator of cell proliferation-like |
| gene-Cfus04607 | interferon-induced very large GTPase 1-like isoform X2 |
| gene-Cfus04608 | up-regulator of cell proliferation-like |
| gene-Cfus04609 | up-regulator of cell proliferation-like |
| gene-Cfus04610 | up-regulator of cell proliferation-like |
| gene-Cfus04611 | CAAX prenyl protease 1 homolog |
| gene-Cfus04612 | inner centromere protein-like isoform X1 |
| gene-Cfus04613 | hypothetical protein PHYPO_G00012690 |
| gene-Cfus04614 | 5-hydroxytryptamine receptor 1D-like |
| gene-Cfus04615 | coiled-coil domain-containing protein 6-like |
| gene-Cfus04616 | protein FAM13A-like |
| gene-Cfus04617 | phytanoyl-CoA hydroxylase-interacting protein-like isoform X1 |
| gene-Cfus04618 | protein bicaudal C homolog 1-B isoform X1 |
| gene-Cfus04619 | paramyosin-like isoform X2 |
| gene-Cfus04620 | ubiquitin-conjugating enzyme E2 D4-like |
| gene-Cfus04621 | oocyte zinc finger protein XlCOF6-like |
| gene-Cfus04622 | visual system homeobox 1 |
| gene-Cfus04623 | ectonucleoside triphosphate diphosphohydrolase 6 isoform X3 |
| gene-Cfus04624 | barrier-to-autointegration factor-like protein |
| gene-Cfus04625 | glycogen phosphorylase, brain form |
| gene-Cfus04626 | uncharacterized protein LOC108270429 isoform X2 |
| gene-Cfus04627 | uncharacterized protein LOC108270429 isoform X1 |
| gene-Cfus04628 | uncharacterized protein LOC108270429 isoform X1 |
| gene-Cfus04629 | uncharacterized protein LOC108270429 isoform X1 |
| gene-Cfus04630 | uncharacterized protein LOC108270429 isoform X1 |
| gene-Cfus04631 | uncharacterized protein LOC108270429 isoform X1 |
| gene-Cfus04632 | aftiphilin-like isoform X2 |
| gene-Cfus04633 | E3 ubiquitin-protein ligase pellino homolog 1-like |
| gene-Cfus04634 | UTP--glucose-1-phosphate uridylyltransferase isoform X1 |
| gene-Cfus04635 | malate dehydrogenase, cytoplasmic-like |
| gene-Cfus04636 | WD repeat-containing and planar cell polarity effector protein fritz |
| gene-Cfus04637 | hypothetical protein PHYPO_G00012910 |
| gene-Cfus04638 | EH domain-binding protein 1 |
| gene-Cfus04639 | N-acetyllactosaminide beta-1,3-N-acetylglucosaminyltransferase 2-like |
| gene-Cfus04640 | kinesin heavy chain-like isoform X2 |
| gene-Cfus04641 | exportin-1 |
| gene-Cfus04642 | exportin-1 isoform X1 |
| gene-Cfus04643 | peroxisome biogenesis factor 13 |
| gene-Cfus04644 | PDZ domain-containing protein 8 |
| gene-Cfus04645 | synaptic vesicular amine transporter |
| gene-Cfus04646 | potassium channel subfamily K member 18 |
| gene-Cfus04647 | cytosolic phospholipase A2 zeta-like, partial |
| gene-Cfus04648 | cytosolic phospholipase A2 zeta-like |
| gene-Cfus04649 | cytosolic phospholipase A2 zeta-like |
| gene-Cfus04650 | cytosolic phospholipase A2 zeta-like |
| gene-Cfus04651 | hypothetical protein cypCar_00006257 |
| gene-Cfus04652 | ventral anterior homeobox 1 |
| gene-Cfus04653 | V-type proton ATPase subunit B, brain isoform |
| gene-Cfus04654 | shootin-1 |
| gene-Cfus04655 | enolase 4 isoform X2 |
| gene-Cfus04656 | heat shock 70 kDa protein 12A isoform X1 |
| gene-Cfus04657 | -- |
| gene-Cfus04658 | monoacylglycerol lipase ABHD12-like |
| gene-Cfus04659 | monoacylglycerol lipase ABHD12-like isoform X2 |
| gene-Cfus04660 | zinc finger protein Rlf |
| gene-Cfus04661 | phospholipase B1, membrane-associated-like |
| gene-Cfus04662 | la-related protein 1B-like isoform X1 |
| gene-Cfus04663 | membrane-associated progesterone receptor component 2 |
| gene-Cfus04664 | acetylserotonin O-methyltransferase-like |
| gene-Cfus04665 | protein Jade-1 isoform X2 |
| gene-Cfus04666 | noncompact myelin-associated protein |
| gene-Cfus04667 | calcipressin-3-like isoform X1 |
| gene-Cfus04668 | NIPA-like protein 3 |
| gene-Cfus04669 | grainyhead-like protein 3 homolog isoform X1 |
| gene-Cfus04670 | claudin 23b |
| gene-Cfus04671 | connector enhancer of kinase suppressor of ras 1 |
| gene-Cfus04672 | ribosomal protein S6 kinase alpha-1 isoform X2 |
| gene-Cfus04673 | TMF-regulated nuclear protein 1 |
| gene-Cfus04674 | arf-GAP with SH3 domain, ANK repeat and PH domain-containing protein 3-like |
| gene-Cfus04675 | transcription factor E2F2 isoform X1 |
| gene-Cfus04676 | DNA-binding protein inhibitor ID-3-A |
| gene-Cfus04677 | peptide methionine sulfoxide reductase MsrA |
| gene-Cfus04678 | YTH domain-containing family protein 2 |
| gene-Cfus04679 | transcription cofactor vestigial-like protein 2 |
| gene-Cfus04680 | uncharacterized protein LOC107697653 |
| gene-Cfus04681 | cathepsin L precursor |
| gene-Cfus04682 | cathepsin L precursor |
| gene-Cfus04683 | solute carrier family 2, facilitated glucose transporter member 1-like |
| gene-Cfus04684 | -- |
| gene-Cfus04685 | SPARC-related modular calcium-binding protein 1 isoform X1 |
| gene-Cfus04686 | SPARC-related modular calcium-binding protein 1 |
| gene-Cfus04687 | coiled-coil domain-containing protein 177 |

**Figure legends**

Supplementary Figure S1 Distribution of (A) gene lengths, (B) coding sequence (CDS) lengths, (C) exon lengths, (D) intron lengths, (E) exon numbers, (F) intron numbers, (G) gene GC content and (H) CDS GC content in *Clarias fuscus* and other teleosts.

Supplementary Figure S2 Gene family comparison between *Clarias fuscus* and other fish species.


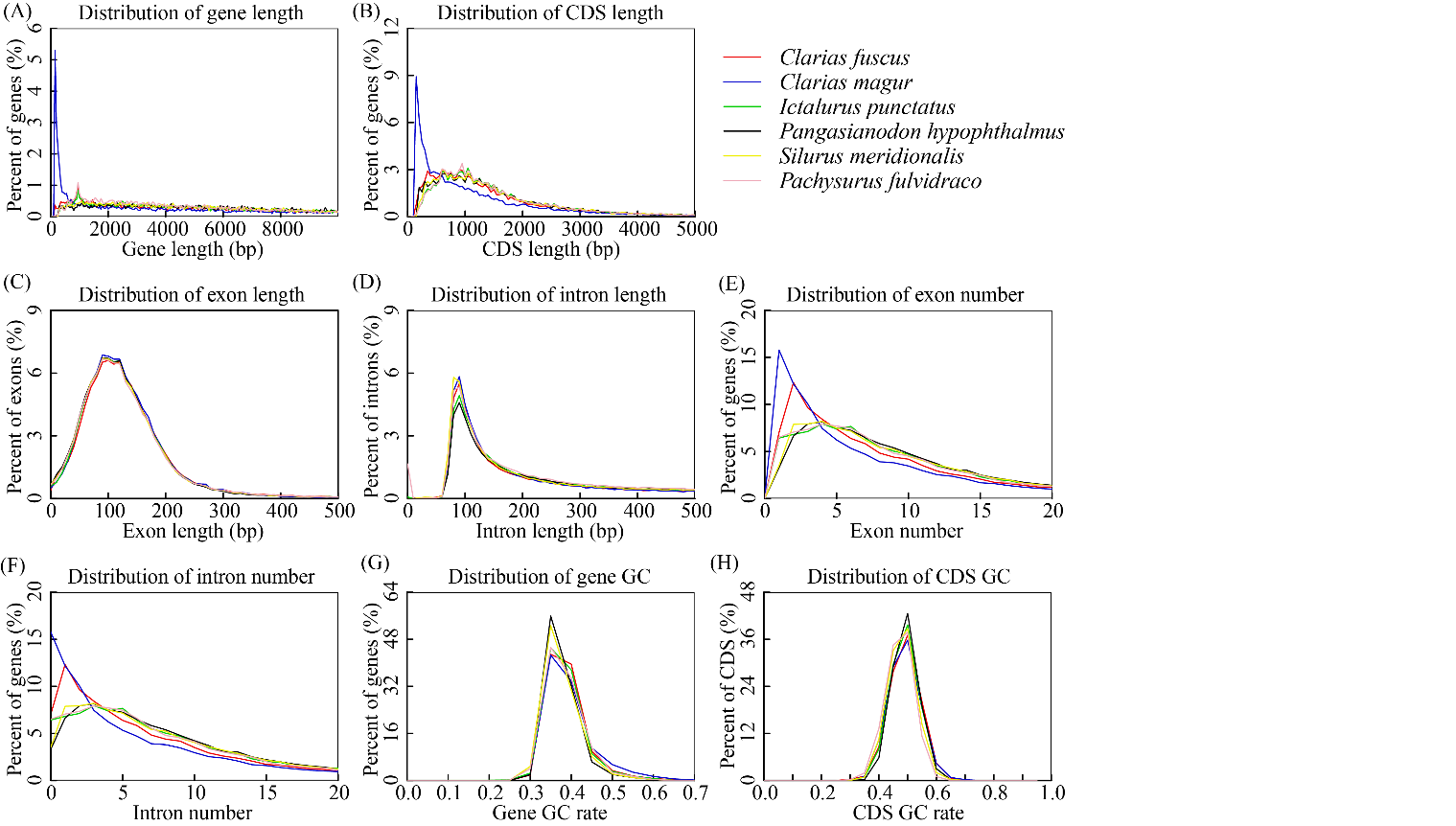


Supplementary Figure S1 Distribution of (A) gene lengths, (B) coding sequence (CDS) lengths, (C) exon lengths, (D) intron lengths, (E) exon numbers, (F) intron numbers, (G) gene GC content and (H) CDS GC content in *Clarias fuscus* and other teleosts.


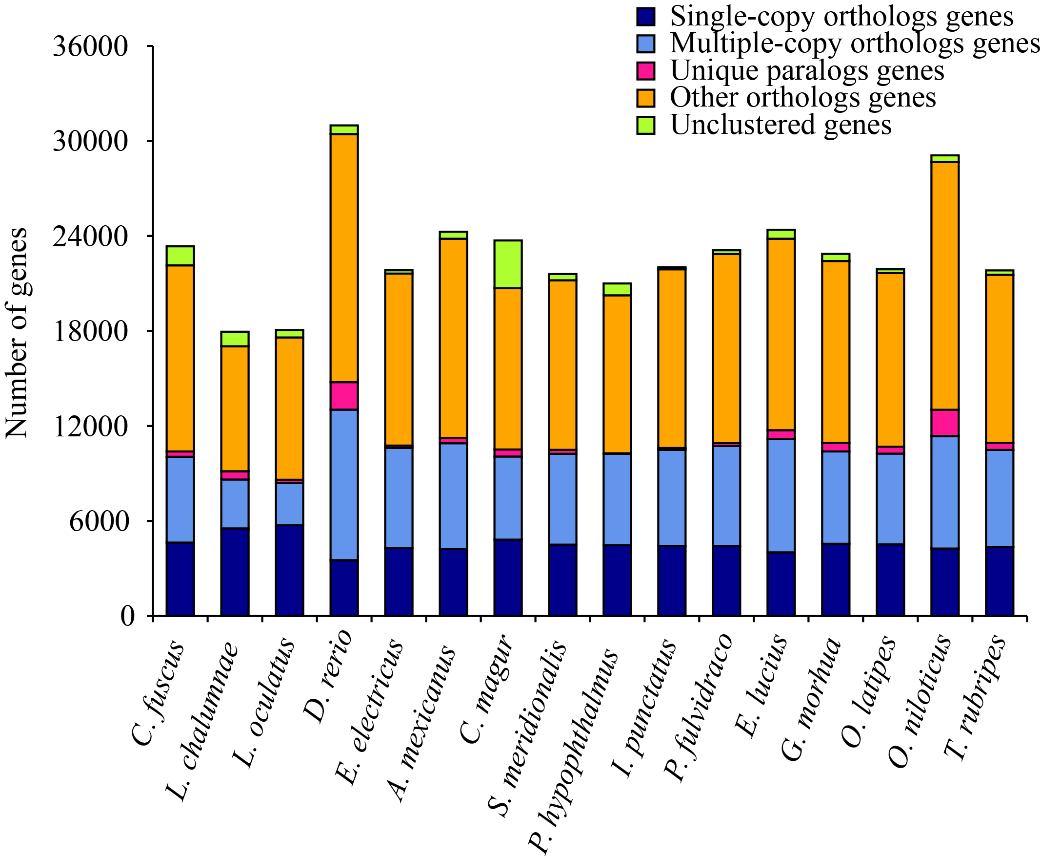


Supplementary Figure S2 Gene family comparison between *Clarias fuscus* and other fish species.
